# Factors associated with plasmid antibiotic resistance gene carriage revealed using large-scale multivariable analysis

**DOI:** 10.1038/s41598-023-29530-y

**Published:** 2023-02-13

**Authors:** Alex Orlek, Muna F. Anjum, Alison E. Mather, Nicole Stoesser, A. Sarah Walker

**Affiliations:** 1 HCAI, Fungal, AMR, AMU & Sepsis Division, UK Health Security Agency, London, UK; 2 Nuffield Department of Medicine, University of Oxford, Oxford, UK; 3 NIHR Health Protection Research Unit in Healthcare Associated Infections and Antimicrobial Resistance, University of Oxford, Oxford, UK; 4 Department of Bacteriology, Animal and Plant Health Agency, Weybridge, Addlestone, UK; 5 Quadram Institute Bioscience, Norwich, UK; 6 University of East Anglia, Norwich, UK; 7 NIHR Oxford Biomedical Research Centre (BRC), University of Oxford, Oxford, UK

**Keywords:** Antimicrobial resistance, Genetic databases, Statistical methods, Bacterial genetics, Epidemiology

## Abstract

Plasmids are major vectors of bacterial antibiotic resistance, but understanding of factors associated with plasmid antibiotic resistance gene (ARG) carriage is limited. We curated > 14,000 publicly available plasmid genomes and associated metadata. Duplicate and replicate plasmids were excluded; where possible, sample metadata was validated externally (Bac*Dive* database). Using Generalised Additive Models (GAMs) we assessed the influence of 12 biotic/abiotic factors (e.g. plasmid genetic factors, isolation source, collection date) on ARG carriage, modelled as a binary outcome. Separate GAMs were built for 10 major ARG types. Multivariable analysis indicated that plasmid ARG carriage patterns across time (collection years), isolation sources (human/livestock) and host bacterial taxa were consistent with antibiotic selection pressure as a driver of plasmid-mediated antibiotic resistance. Only 0.42% livestock plasmids carried carbapenem resistance (compared with 12% human plasmids); conversely, tetracycline resistance was enriched in livestock vs human plasmids, reflecting known prescribing practices. Interpreting results using a timeline of ARG type acquisition (determined by literature review) yielded additional novel insights. More recently acquired ARG types (e.g. colistin and carbapenem) showed increases in plasmid carriage during the date range analysed (1994–2019), potentially reflecting recent onset of selection pressure; they also co-occurred less commonly with ARGs of other types, and virulence genes. Overall, this suggests that following acquisition, plasmid ARGs tend to accumulate under antibiotic selection pressure and co-associate with other adaptive genes (other ARG types, virulence genes), potentially re-enforcing plasmid ARG carriage through co-selection.

## Introduction

Plasmids are extrachromosomal DNA elements common among bacteria. Plasmids are major vectors of antibiotic resistance^1^, but the biotic or abiotic factors associated with plasmid carriage of antibiotic resistance genes (ARGs) remain unclear. Plausible factors include those determining acquisition, maintenance, and spread of plasmid-mediated resistance^2^. ARG acquisition from co-resident plasmids or the host chromosome occurs through transposition, integron-associated gene cassette integration, and homologous recombination between multi-copy elements^3^. Analyses of the genetic contexts of plasmid ARGs support the role of transposons and integrons in plasmid ARG acquisition, and spread among co-resident plasmids^4,5^. Additionally, horizontal plasmid transfer (e.g. conjugation) brings together different plasmids, increasing opportunities for plasmid ARG exchange.

Antibiotic resistance plasmid success among bacterial populations depends on vertical and horizontal transmission^2^. All plasmids control self-replication to maintain a stable copy number within host bacterial cells, enabling vertical inheritance. Plasmids can also transmit horizontally (between host cells), enabling resistance plasmid dissemination across strains and species^6^. Conjugation is a key horizontal transfer mechanism that is plasmid-encoded. Conjugative plasmids encode complete conjugative systems; mobilisable plasmids lack genes required for mating-pore formation but can conjugate if functions are provided *in trans* by a co-resident plasmid; non-mobilisable plasmids are unable to conjugate^7,8^. Antibiotic selection pressure influences vertical/horizontal pathways of resistance plasmid dissemination. Under antibiotic selection pressure, resistance plasmids may benefit bacterial hosts and disseminate vertically through clonal expansion. Without selection pressure, antibiotic resistance plasmids might impose fitness costs. However, compensatory mutations may alleviate fitness costs. Although mutations are generally deleterious^9^, they can also provide plasmids with the plasticity needed to adapt to shifting selection pressures, including variable antibiotic selection pressures^10,11^, and new host strains^12^; plasmid transposable elements have been implicated in genome rearrangement and gene deletion/inactivation events, which were linked to compensatory adaptive evolution^13–15^. Alternatively, conjugative transfer or toxin-antitoxin systems may enable plasmids to persist as genetic parasites, despite fitness costs^16,17^. Finally, co-selection of linked adaptive genes (e.g. ‘co-resistance’ between different ARGs) can promote persistence of antibiotic resistance plasmids under multiple selective conditions^18,19^.

The genetic/evolutionary mechanisms of plasmid-mediated resistance have generally been studied in vitro^16^ and through small/local-scale observational genomic studies^18^, with limited large-scale real-world analyses considering the independent effects of different factors. Quantitative multivariable analyses investigating associations between plasmid ARG carriage and biotic/abiotic factors may afford novel insights^20–25^. The National Center for Biotechnology Information (NCBI) Nucleotide and BioSample databases are rich sources of sequencing data and linked metadata, with which to investigate such associations^26^, but should be used cautiously due to data quality issues including redundancy, sampling bias, and low-quality metadata^27^. In one study of NCBI plasmids, Pal et al. analysed ~ 4500 NCBI plasmids and found a positive co-association between biocide/metal resistance genes and ARGs^24^. More recently, Che et al. analysed ~ 14,000 NCBI plasmids and found that conjugative plasmids and insertion sequences were positively associated with ARG presence^25^. However, in both studies a relatively small number of (plasmid genetic) factors were analysed quantitatively, with limited control of confounding factors. For example, while Pal et al. used multivariable analysis to investigate biocide/metal resistance genes, controlling for plasmid size, other factors (e.g. host strain taxonomy, sample isolation source) were analysed descriptively.

Here, we curated a dataset of NCBI plasmids and metadata, then fitted Generalised Additive Models (GAMs) to investigate factors associated with plasmid ARG carriage, across 10 major ARG types. GAMs model continuous variables using flexible smooth functions, helping to uncover complex non-linear patterns. We included a wide range of both biotic (e.g. plasmid genetic) and abiotic factors (e.g. isolation source) in our multivariable models (selected based on a priori biological knowledge), with the aim of gaining broad insights into the associated factors, while controlling for confounding bias. To help gain overarching insights, we ordered ARG types into a timeline of first recorded plasmid-mediated resistance, based on literature review (e.g. colistin being most recently acquired). Interpreting results according to this timeline suggested a plausible trajectory of plasmid-mediated resistance: following acquisition, plasmid ARGs accumulate under selection pressure; over time, they tend to co-associate with other adaptive genes (ARGs of other types, virulence genes).

## Results

### A curated dataset of complete plasmids and associated sample metadata

After retrieval from NCBI and initial automated curation (including deduplication of identical plasmids), there were 16,270 plasmids. Manual examination of a subset of identical plasmids suggested that plausible examples of independently isolated plasmids with 100% identical sequences are encountered, but very rarely—even among identical plasmids with differing metadata, only 4 such plausible examples were identified—justifying automated deduplication of identical plasmids (Supplementary Results 2.1). Of the 16,270 plasmids from the initial automated curation, 11,848 (73%) were linked to a BioSample accession (5195 BioSample accessions) (Supplementary Fig. 6). Note that multiple plasmids may be linked to the same BioSample accession (“sample” for brevity), reflecting plasmid biology (isolates can contain multiple plasmids). While all downstream analysis was conducted at the plasmid-level, sample metadata was used to inform plasmid curation steps. For example, laboratory/commercial samples were identified from metadata, and plasmids linked to these samples were excluded (189 plasmids linked to 76 samples) (Supplementary Fig. 6). As well as informing plasmid curation, sample metadata was itself curated. Of the 5195 samples in the initially retrieved/curated dataset (see above), 3604 (69%) were assigned a geocoded coordinate and 1207 (23%) had a valid *lat_lon* coordinate. Among 1201 samples with both coordinates available, there were 43 discrepancies (internal consistency rate = 96%) (Supplementary Data 1). Bac*Dive* metadata was available for 236/655 identified culture collection samples. Following Bac*Dive*-guided curation, metadata additions and/or corrections were made for 116/236 (49%) samples. 29 corrections were in the collection date field, of which nine could be attributed to species taxonomic authority date (i.e. taxonomic description date) being given instead of genuine collection date. Wider examination of collection dates (all pre-1950 dates) revealed four additional discrepancies attributable to inter-changing taxonomic authority/collection date (Supplementary Data 1b, c).

After all curation steps, there were 14,143 plasmids (Supplementary Fig. 6, Supplementary Data 1), of which 3939 contained ≥ 1 ARG, and 3639 contained ≥ 1 of the major 10 ARG types included in statistical analysis. For the 3639 major ARG-encoding plasmids, a Microreact visualisation shows the global spatiotemporal distribution, by isolation source (https://microreact.org/project/ncbi-plasmid-antibiotic-resistance). Table 1 shows the distribution of explanatory variables for plasmids encoding/not encoding major ARGs.

**Table 1:** Plasmid characteristics for plasmids encoding/not encoding any of the 10 major ARG types.

Explanatory variables	Measures	
	≥ 1 major ARG type (n = 3639)	No major ARG type (n = 10,504)
log_10_ Plasmid size*	4.85 [4.59, 5.11]	4.60 [3.95, 4.99]
Insertion sequence density (frequency per 10 kb)*	0.40 [0.23, 0.63]	0.04 [0.00, 0.35]
Collection date (years since initial collection year [1994])*	21.00 [18.00, 23.00]	18.00 [14.00, 21.00]
Integron present	1232 (33.9)	9 (0.1)
Biocide/metal resistance gene present	1486 (40.8)	572 (5.4)
Virulence gene present	226 (6.2)	933 (8.9)
Conjugative system		
Non-mobilisable	847 (23.3)	5533 (52.7)
Mobilisable	823 (22.6)	3110 (29.6)
Conjugative	1969 (54.1)	1861 (17.7)
Replicon carriage^†^		
Untyped	637 (17.5)	7593 (72.3)
Single-replicon	1995 (54.8)	2226 (21.2)
Multi-replicon	1007 (27.7)	685 (6.5)
Host taxonomy		
Enterobacteriaceae	2558 (70.3)	2072 (19.7)
Proteobacteria (non-Enterobacteriaceae)	593 (16.3)	3767 (35.9)
Firmicutes	391 (10.7)	2543 (24.2)
Other	97 (2.7)	2122 (20.2)
Geographic location^††^		
High-income not elsewhere classified	257 (7.1)	1430 (13.6)
Middle-income not elsewhere classified	183 (5.0)	772 (7.3)
European Union (EU) and United Kingdom	172 (4.7)	1070 (10.2)
China	286 (7.9)	962 (9.2)
United States	351 (9.6)	1140 (10.9)
Other	2390 (65.7)	5130 (48.8)
Isolation source		
Human	1024 (28.1)	1930 (18.4)
Livestock	152 (4.2)	556 (5.3)
Other	2463 (67.7)	8018 (76.3)

Note: Showing median [IQR] for continuous variables and n (%) for categorical variables. The number of other antibiotic resistance gene (ARG) types is not shown as a continuous variable since it was determined per ARG type model rather than for the overall dataset. The number of other ARG types was winsorised at the modal 95th percentile which corresponded to four other ARG types (the 95th percentile for the colistin model was five other ARG types, and the 95th percentile for all other models was four other ARG types). “Other” factor levels include plasmids assigned to a rare category or not assigned a category (if BioSample metadata was missing; or for isolation source, non-human/livestock metadata was left un-curated and assignment to specific categories was not attempted). For host taxonomy, taxonomic metadata was present for all plasmids, but sometimes only “uncultured bacterium” (n = 261); for geographic location, metadata was missing for 7284 plasmids; for isolation source, metadata was missing for 4317 plasmids.

*Continuous variables were winsorised (limiting extreme values to threshold values) at the 5th/95th percentiles on both tails (plasmid size, 2.9–311.7 kb); right tail (insertion sequence density, 1.05 per 10 kb); left tail (collection date, 1994).

^†^For replicon carriage, the factor level reflects the number of unique replicon types detected (e.g. IncFIB, IncFIC type is categorised multi-replicon whereas IncFIC, IncFIC is categorised single-replicon).

^††^For geographic location, the middle-income not elsewhere classified (n.e.c.) category represents World Bank lower-middle income and upper-middle income countries combined, and not elsewhere classified. China is an upper-middle income category; the United Kingdom and United States are high-income countries, and European Union countries are all high-income except for Romania (upper-middle income) (income categorisations based on World Bank 2018 income groupings).

### A timeline of plasmid ARG acquisition determined by literature review

Literature review of the major ARG types indicated that plasmid-mediated ARG acquisition occurred most recently for colistin (article date 2016), quinolone (1998), carbapenem (1991), and ESBL (1983) resistance. Earlier plasmid-mediated resistance was recorded for trimethoprim (1972) and macrolide (1963) resistance. The earliest article was for aminoglycoside, sulphonamide, tetracycline and phenicol (multi-)resistance (1956) (Table 2). This order is concordant with the order of initial collection dates recorded for each ARG type, among plasmids in our study; the initial collection dates helped resolve the order of aminoglycoside/sulphonamide (1965 collection date), tetracycline (1969), and phenicol (1971) (Table 2). The inferred timeline of plasmid-mediated ARG acquisition (and hence, the likely timeline of selection pressure onset) appeared to influence some of the ARG carriage patterns revealed by GAM modelling and exploratory ARG type co-occurrence analysis, as described below.

**Table 2:** First reported plasmid-mediated resistance for 10 major ARG types, ordered by date of first published article/first collection date.

ARG type	First article published	First isolate collected	Reference(s)	Notes on articles	First NCBI collection date	First NCBI accession id
Aminoglycoside	1956	1955	Kitamoto et al., 1956^60^ Watanabe, 1963^61^ Watanabe, 1967^62^	Kitamoto et al., 1956 is a Japanese language article. Watanabe, 1963 is the first report reviewing findings on transferable aminoglycoside sulphonamide, tetracycline, phenicol multi-resistance in English	1965	NZ_CP009357.1
Sulphonamide					1965	NZ_CP009357.1
Tetracycline					1969	NZ_CP007650.1
Phenicol					1971	NZ_CP005999.1
Macrolide	1963	Unknown	Mitsuhashi et al., 1963^63^ Novick and Richmond, 1965^64^ Novick et al., 1979^65^	Mitsuhashi et al., 1963 describe initial investigations into “strain 258”. Extrachromosomal inheritance of erythromycin (macrolide) resistance in “strain 258” is reported by Novick and Richmond, 1965. Novick et al., 1979 presents a genetic map of plasmid pI258 showing *repA* (plasmid replication locus) and *ermB* (erythromycin resistance gene)	1986	NZ_CP028340.1
Trimethoprim	1972	1971	Fleming et al., 1972^66^	Fleming et al., 1972 demonstrates transferable trimethoprim resistance	1987	NZ_CP034238.1
ESBL	1983	Unknown	Knothe et al., 1983^67^ Bush, 2018^68^	See Table 1 in Bush, 2018	1994	NZ_CP014698.2
Carbapenem	1991	1988	Watanabe et al., 1991^69^ Bush, 2018^68^	See Table 1 in Bush, 2018	1997	KY984047.1
Quinolone	1998	1994	Martínez-Martínez et al., 1998^70^	Prior reports were unconfirmed (see Courvalin, 1990^71^)	1998	NZ_CP012139.1
Colistin	2016	2011	Liu et al., 2016^72^	Liu et al., 2016 is the first report of plasmid-mediated colistin (*mcr-1*) resistance. Retrospective studies have indicated sporadic presence of transferable colistin resistance as early as the 1980s^73,74^	2001	NZ_AP018799.1

The table summarises results of a literature review, conducted to retrieve the first article supporting plasmid-mediated resistance, for each of the 10 major antibiotic resistance gene (ARG) types analysed in our study. First recorded collection dates among the 14,143 NCBI plasmids analysed in our study (and corresponding NCBI accession ids) are also given. ARG types (table rows) are ordered by date of the first published article (“First article published” column), from earliest (top) to latest (bottom). This ordering is concordant with the order of first collection dates recorded for plasmids in our study; although collection dates were used to split a tie (the earliest 4 ARG types were associated with same first article publication date). The table details additional information on the published articles: if reported in the article, corresponding isolate collection dates are given (“First isolate collected” column). References to the first published article, as well as any later supporting article, are provided, along with explanatory notes.

### The earliest acquired ARG types co-occurred more frequently with other ARG types

**Figure 1:**
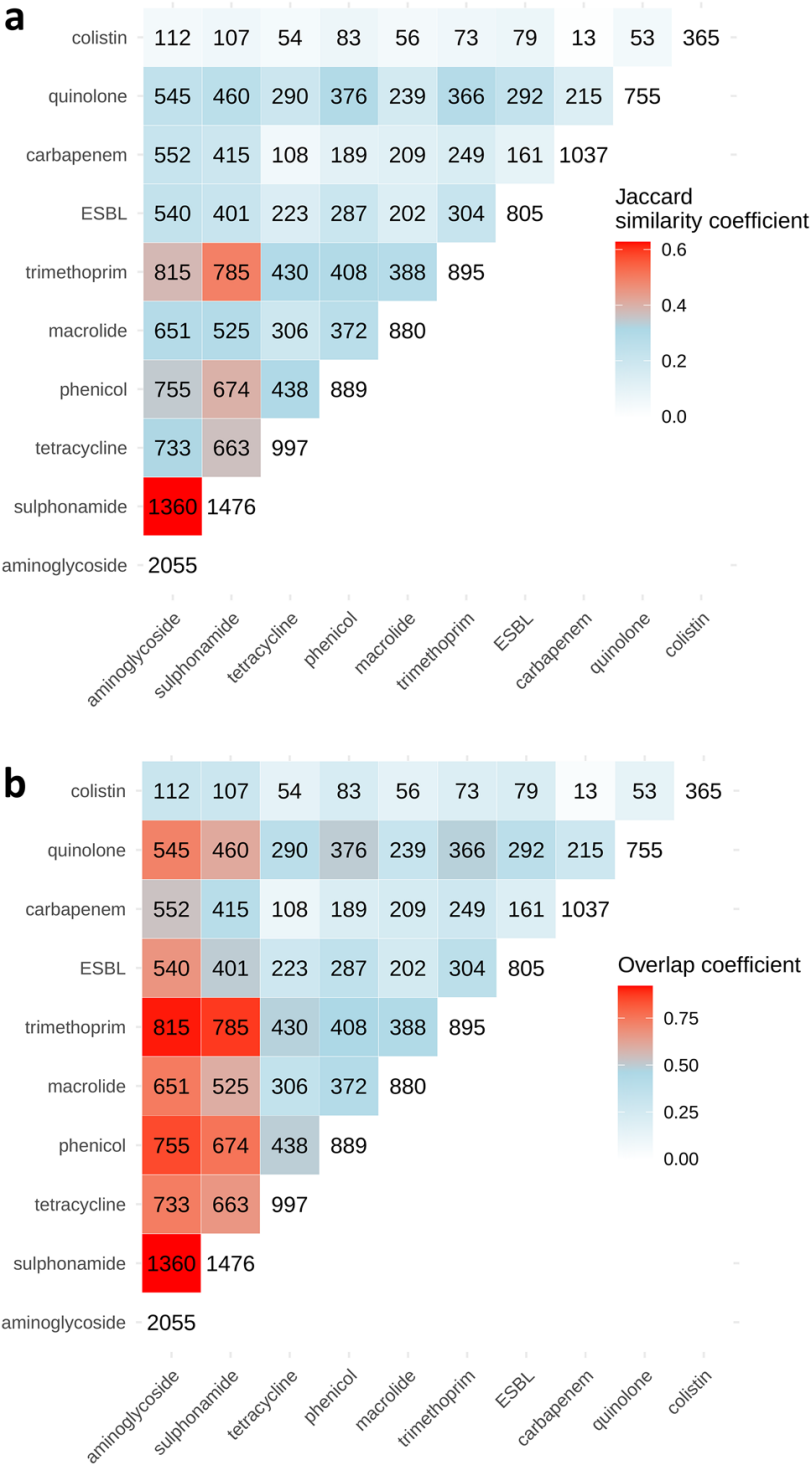
Co-occurrence of antibiotic resistance gene (ARG) types is determined from their presence/absence in the dataset of 14,143 plasmids, and visualised using heatmaps. ARG types are ordered by the inferred timeline of known plasmid-mediated resistance acquisition (see Table 2) from earliest (aminoglycoside, sulphonamide) to most recent (colistin). Counts along the diagonal indicate total plasmids carrying a given ARG type. Counts in the upper-left triangle indicate pairwise ARG type intersections i.e. the number of plasmids where a given pair of ARG types co-occur. Heatmaps are coloured by similarity metrics ((**a**) Jaccard index, (**b**) overlap coefficient) indicating the degree of co-occurrence between ARG types (red = more co-occurrence; light blue = less co-occurrence). Heatmaps were generated using custom R code available in a GitHub repository (PlasmidARGCarriage v1.0). (https://github.com/AlexOrlek/PlasmidARGCarriage/blob/v1.0/exploratory_analysis.R).

Figure 1 visualises pairwise co-occurrence between ARG types, based on ARG type presence/absence in the curated plasmid dataset. Overall, aminoglycoside and sulphonamide (the earliest acquired ARG types) were the most frequently co-occurring ARG types; they co-occurred frequently with each other (Jaccard index 0.63, overlap coefficient 0.92), and with other ARG types. By contrast, colistin (the most recently acquired ARG type) co-occurred least frequently with other ARG types; this was not simply due to the size differential (colistin being the smallest ARG type) because overlap coefficients were lower, as well as Jaccard indices (Fig. 1a,b). Similar overall co-occurrence patterns were found when restricting to 1007 plasmids with collection dates from 2016–2019 (reducing potential bias from plasmids with earlier collection dates, preceding the emergence of more recently acquired ARG types) (Supplementary Fig. 7). Further analysis of the aminoglycoside–sulphonamide co-occurrences represented in Fig. 1, at the gene-level, showed that the most frequently linked ARG pairs included *aph(3'')-Ib*–*sul2* and *aph(6)-Id*–*sul2* (Supplementary Fig. 8). The patterns of co-occurrence found in Fig. 1 are reflected in the statistical modelling analysis, described below, where co-occurrence is examined in terms of number of other co-occurring ARG types (see Fig. 3a).

### Recently acquired ARG types showed recent increases in plasmid carriage

**Figure 2:**
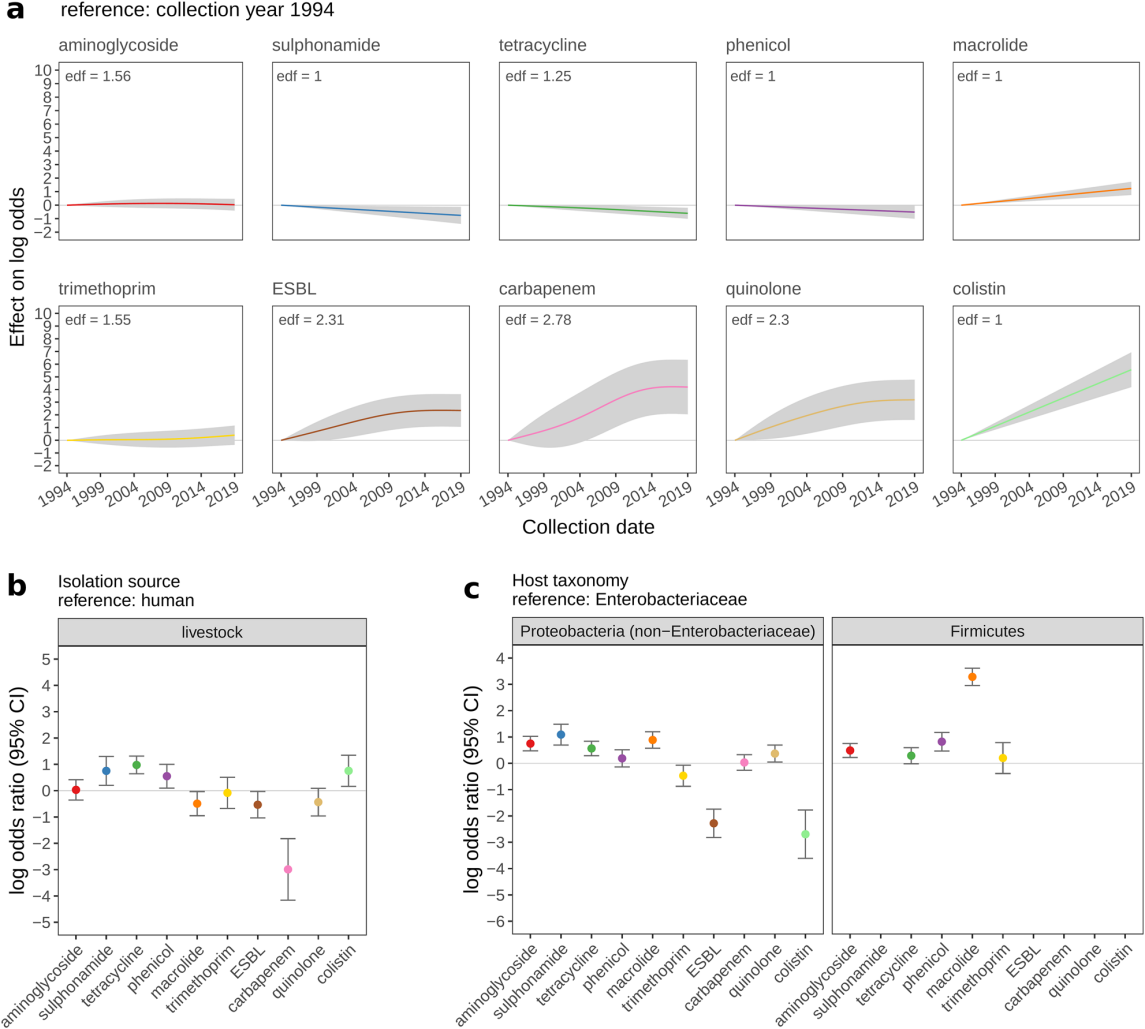
Multivariable-adjusted association between presumed proxies of antibiotic selection pressure and the log-odds of antibiotic resistance gene (ARG) carriage. The effect of (**a**) collection date; (**b**) isolation source; (**c**) host taxonomy on the log-odds of ARG carriage (y-axis), is shown across 10 ARG types. ARG types are ordered by the date plasmid-mediated resistance was first reported (see Table 2) from earliest (aminoglycoside, sulphonamide) to most recent (colistin). For categorical factors (isolation source, host taxonomy), log-odds ratios indicate the effect of a given factor level, relative to reference factor level, and error bars show 95% confidence intervals. For collection date, smooth curves indicate the predicted effect of sample collection date. An effective degrees of freedom (edf) value > 1 indicates a non-linear smooth. Shaded areas around smooths show 95% confidence intervals. No Firmicutes plasmids encoded known sulphonamide, ESBL, quinolone, or colistin resistance genes, and only 2 Firmicutes plasmids encoded known carbapenem resistance genes; therefore, corresponding odds ratios are not shown.

From GAM modelling, we found that patterns of ARG type carriage over time appeared to reflect the timeline of plasmid-mediated ARG acquisition (Fig. 2a): there were increases in ESBL, carbapenem, and quinolone carriage from 1994 (reference collection year) which plateaued in later years; colistin (acquired most recently) showed an increase from the point of acquisition (first collection date 2001—the extrapolation of the smooth line prior to this is not meaningful). For the earliest acquired types—aminoglycoside, sulphonamide, tetracycline, and phenicol—there was no evidence of multivariable-adjusted association between ARG carriage and collection year. In the unadjusted analysis, increases with collection year were weaker for earlier vs more recently acquired ARGs (Supplementary Fig. 17). Results were similar when restricting unadjusted analysis to a dataset of 6375 plasmids with non-imputed collection dates only (Supplementary Fig. 18).

### Plasmid isolation source (human/livestock) and bacterial host taxonomy showed strong signals of association with ARG carriage patterns

Regarding isolation source, as expected given prescribing practices, carbapenem resistance was rare in livestock-associated plasmids (only 0.42% [3/708] livestock-associated plasmids encoded carbapenem resistance, versus 12% [356/2954] human-associated plasmids). The multivariable model indicated tetracycline resistance was more common in livestock vs human-associated plasmids (odds-ratio [OR] = 2.65, 95% confidence interval [CI] 1.89–3.72, *p < *0.0001; Fig. 2b). Regarding host taxonomy, major ARGs were more common among Enterobacteriaceae plasmids (Table 1; Supplementary Fig. 12a); 55% Enterobacteriaceae plasmids encoded ≥ 1 major ARG, compared with 14%, and 13% plasmids respectively, from non-Enterobacteriaceae Proteobacteria and Firmicutes taxa. Among Firmicutes taxa, no plasmids encoded known sulphonamide, ESBL, quinolone, or colistin ARGs, and only two Firmicutes plasmids encoded carbapenem resistance. Among non-Enterobacteriaceae Proteobacterial taxa, unadjusted and adjusted analysis indicated fewer plasmids carried ESBL, and colistin ARGs compared with Enterobacteriaceae plasmids (Fig. 2c). Overall, there were marked differences between unadjusted/adjusted effects, suggesting strong confounding; negative unadjusted associations between ARG carriage and non-Enterobacterial plasmids were attenuated or reversed in multivariable-adjusted analysis. For example, multivariable-adjusted analysis suggested that Firmicutes plasmids were more likely to carry macrolide ARGs compared with Enterobacterial plasmids (OR = 26.7, 95% CI 19.2–37.1, *p < *0.0001) whereas unadjusted analysis indicated the reverse (OR = 0.69, 95% CI 0.59–0.82, *p < *0.0001) (Fig. 2c). Sensitivity analysis suggested the difference between unadjusted/adjusted effects may at least partly result from confounding with the effects of replicon carriage and number of other ARG types (Supplementary Results 2.7.1). Unadjusted analysis at the level of Firmicutes species showed that compared with Enterobacteriaceae plasmids, Firmicutes plasmids from *Staphylococcus aureus* and *Enterococcus faecium* were more likely to carry macrolide resistance, whereas plasmids from other Firmicutes species were less likely to carry macrolide resistance (Supplementary Fig. 24). Replicon carriage was frequent in *Staphylococcus aureus* and *Enterococcus faecium* (respectively, 94% and 86% plasmids encoded one or more replicon types) (Supplementary Table 3) and less frequent in other major Firmicutes species, which appears to explain why adjusting for factors including replicon carriage led to the reversal of unadjusted effects.

### There were no strong associations between geography and ARG carriage

There were some modest associations between geographic location (modelled as high-income not elsewhere classified [n.e.c.], middle-income n.e.c., EU & UK, China, US, other [i.e. missing or uncategorised]) and ARG type carriage. ARG carriage was more common in plasmids from ‘other’ vs high-income n.e.c. countries for aminoglycoside and carbapenem outcomes in adjusted analyses (ORs [95% CIs] 2.05 [1.46–2.88] and 2.48 [1.71–3.60], respectively, *p < *0.0001) and for most outcomes in unadjusted analysis. Further, in the unadjusted analysis, macrolide, phenicol, and sulphonamide carriage were moderately more common in China while aminoglycoside, carbapenem, and sulphonamide carriage were moderately more common in the United States (Supplementary Fig. 11).

### Earlier acquired ARG types were more commonly found on plasmids encoding virulence genes and multiple other ARG types

**Figure 3:**
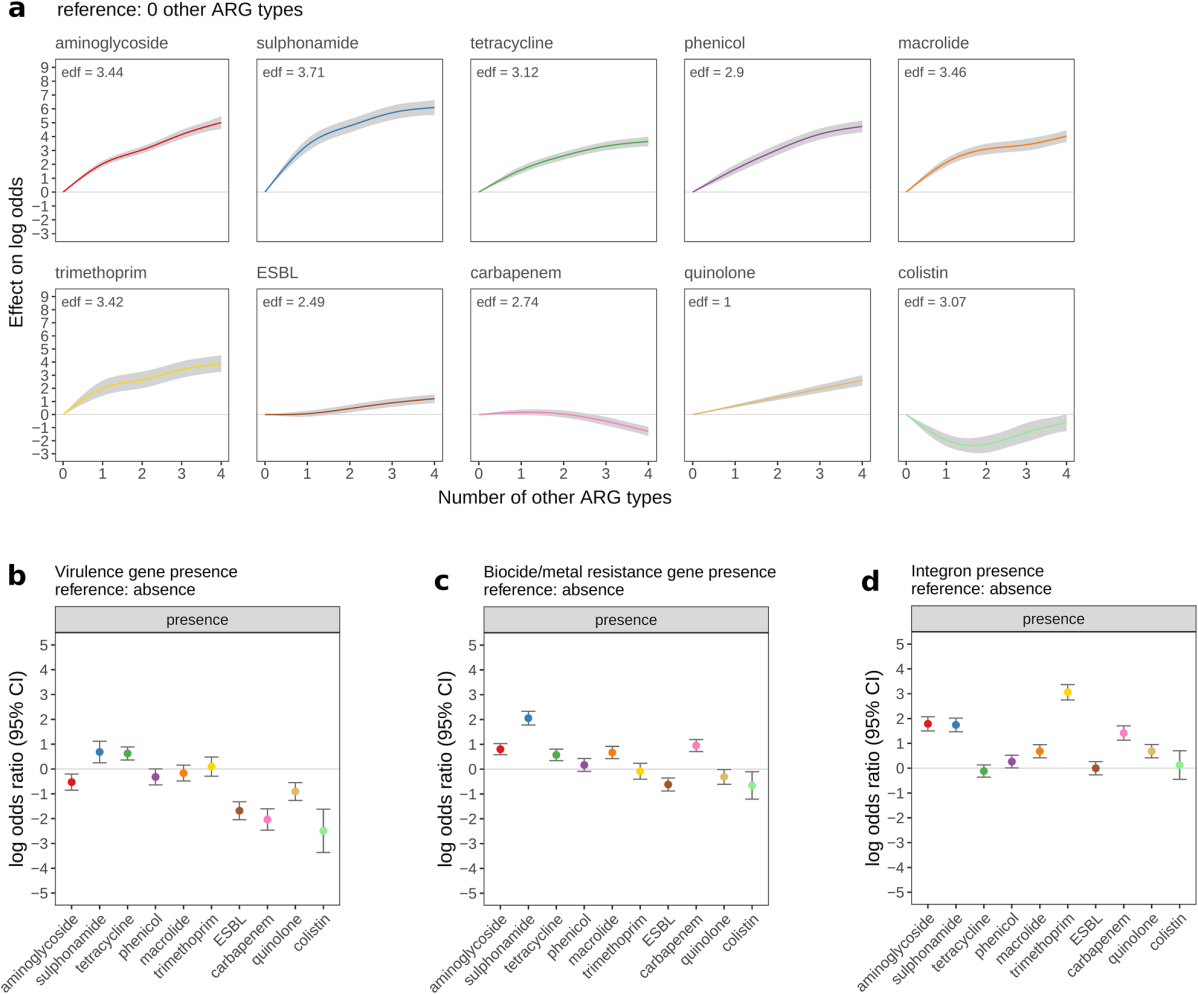
The multivariable-adjusted association between plasmid genetic traits and the log-odds of antibiotic resistance gene (ARG) carriage. The effect of (**a**) number of other ARG types; (**b**) virulence gene presence; (**c**) biocide/metal resistance gene presence; (**d**) integron presence, on the log-odds of ARG carriage (y-axis), is shown across 10 ARG types. ARG types are ordered by the date plasmid-mediated resistance was first reported (see Table 2) from earliest (aminoglycoside, sulphonamide) to most recent (colistin). For categorical factors (biocide/metal resistance gene presence, integron presence, virulence gene presence), log-odds ratios indicate the effect of a given factor level, relative to reference factor level, and error bars show 95% confidence intervals. For number of other ARG types, smooth curves indicate the predicted effect of ≥ 1 other resistance gene types (relative to 0 other ARG types). An effective degrees of freedom (edf) value > 1 indicates a non-linear smooth. Shaded areas around smooths show 95% confidence intervals.

To gain further insight into the associated factors and evolutionary genetic trajectory of plasmid-mediated ARG carriage, we investigated plasmid genetic factors. For a given ARG type outcome, ARG carriage was generally more common for plasmids carrying ≥ 1 other ARG types in both unadjusted and adjusted analyses. However, this trend appeared to be influenced by the timeline of ARG type acquisition. For earlier acquired ARG types (aminoglycoside through to trimethoprim), there were strong increases in ARG carriage for plasmids carrying ≥ 1 other ARG types. Conversely, more recently acquired ARG types, showed weaker associations between ARG carriage and number of other ARG types (Fig. 3a, Supplementary Fig. 20). Similarly for virulence gene presence, unadjusted/adjusted associations between virulence gene presence and ARG carriage appeared to be influenced by the ARG type acquisition timeline; plasmids carrying virulence genes were less likely to carry more recently acquired ARG types, especially colistin and carbapenem (Fig. 3b).

### ARG carriage was more common in plasmids with integrons, biocide/metal resistance, and replicons in unadjusted analysis, but effects were attenuated in adjusted analysis

In unadjusted analyses, ARG carriage was more common in plasmids with biocide/metal resistance and integrons (*p < *0.0001 for all outcomes). Unadjusted effects were strongly attenuated when adjusting for other factors, though ARG carriage still appeared more common in plasmids with biocide/metal resistance and integrons, for some ARG type outcomes (Fig. 3c and d; Supplementary Figs. 9, 13). Confounding between the effects of biocide/metal resistance, integron presence, and number of other ARG types (which were positively associated) is one explanation for the attenuation of unadjusted effects (Supplementary Results 2.7.2). In unadjusted/adjusted analyses, there was particularly strong positive association between integron presence and trimethoprim resistance (adjusted OR [95% CIs] 21.33 [16.67–29.05]). Especially in the unadjusted analysis, patterns of association for biocide/metal resistance and integron presence appeared to be influenced by the timeline of ARG type acquisition, similar to the trend found for number of other ARG types and virulence gene presence. Specifically, for more recently acquired ARG types, there were generally weaker positive associations between ARG carriage and presence of biocide/metal resistance and integrons (Supplementary Figs. 9, 13). There was higher ARG carriage in single-/multi-replicon vs untyped plasmids across all ARG types in unadjusted analyses. In the adjusted analysis, positive associations remained across most ARG types, but were strongly attenuated (Supplementary Fig. 15).

### Carbapenem resistance genes were enriched on conjugative plasmids and plasmids with a higher density of insertion sequences

Overall, there were positive unadjusted/adjusted associations between increasing insertion sequence density and ARG carriage up to ~ 0.3 per 10 kb; at higher insertion sequence densities, ARG carriage plateaued or diminished. In the adjusted analysis, positive associations were generally attenuated, although a strong sustained positive association was found between insertion sequence density and carbapenem resistance gene carriage (increases in odds of ~ 1.5 for every 0.1 higher insertion sequence density) (Supplementary Fig. 19. The unadjusted analysis suggested transmissible plasmids (especially, conjugative plasmids) were associated with ARG carriage across all outcomes, relative to non-mobilisable plasmids (Supplementary Fig. 10). Associations were attenuated in adjusted analyses, with only the positive association between conjugative plasmids and carbapenem resistance remaining (OR = 3.59, 95% CI 2.80–4.61, *p < *0.0001). Complex confounding interrelationships explained attenuation of unadjusted effects (rather than simple confounding with plasmid size alone) (Supplementary Results 2.7.3).

### ARG carriage showed complex non-linear associations with plasmid size

Regarding plasmid size, in unadjusted analysis, ARG carriage was generally more common among larger plasmids (as expected under a null model of larger plasmid, more genes), though the relationship was non-linear; for most ARG types, ARG carriage was less likely below 5–10 kb and peaked among ~ 100 kb plasmids. In adjusted analysis, positive associations were attenuated, but larger plasmids were still more likely to carry ESBL and carbapenem resistance. Small plasmids (< 10 kb) were more likely to carry quinolone resistance (Supplementary Fig. 21). The most frequent quinolone resistance genes among < 10 kb plasmids were *qnrD1*, *qnrS2*, and *qnrB19*; they were primarily found on Col3M, IncQ2, and Col4401 replicon type plasmids, respectively (Supplementary Table 8).

## Discussion

Using multivariable analysis of a curated collection of 14,143 plasmids, we identified key biotic and abiotic factors associated with plasmid ARG carriage, across 10 major ARG types. To facilitate interpretation, dates of first reported plasmid-mediated resistance for each ARG type were determined by literature review (most recent for colistin, carbapenem and quinolone resistance; earliest for aminoglycoside, sulphonamide, tetracycline, and phenicol resistance). Previous studies have identified associations between antibiotic selection pressure (antibiotic usage) and plasmid-mediated resistance^20,28^, but have been restricted in geographic/temporal/taxonomic scope. Here, plasmid ARG carriage patterns across time (collection years), isolation sources (human/livestock), and host bacterial taxa were consistent with antibiotic selection pressure as a driver of plasmid-mediated antibiotic resistance. Collection date data was low quality (55% dates imputed using plasmid sequence publication date), but patterns of association and their concordance with the timeline of plasmid ARG type acquisition were maintained when analysis was restricted to plasmids with known (non-imputed) collection dates only. The finding that more recently acquired ARG types showed increases in plasmid carriage during the dates range analysed (1994–2019) potentially reflects recent antibiotic selection (and/or co-selection) pressures. However, trends may be influenced by sampling bias—for example, recent interest in plasmid-mediated colistin resistance may at least partly explain the strong sustained increase in colistin resistance found in this study; with ongoing monitoring, it will be interesting to see how trends unfold (e.g. whether plasmid-mediated colistin resistance will ultimately decline given that usage is now more restricted)^29^.

Global antibiotic prescribing data indicates that tetracyclines are the most frequently used antibiotic class in livestock, but not in humans where penicillins are most frequently used^30,31^. Specifically, tetracyclines are reported to account for ~ 40% of total global livestock antibiotic usage (in tonnes, calculated from sales/import data)^31^. Normalised by estimated biomass, according to European data, tetracycline consumption in livestock and humans was 33 and 3.1 mg per kg biomass, respectively (i.e. substantially higher in livestock)^32^. In contrast to the pattern of tetracycline usage, guidelines restrict carbapenem usage to human medicine only^33–35^. Accordingly, in livestock vs human plasmids, we found tetracycline resistance to be more common whereas carbapenem resistance was less common, presumably reflecting selection pressures from differing prescribing practices. Host bacterial taxonomy can also be considered a proxy for antibiotic selection pressure—plasmids from clinically important taxa may face elevated selection pressure; in addition selection pressure is likely to vary according to differing antibiotic resistance mechanisms/activity spectra across taxa (e.g. if resistance occurs chromosomally/intrinsically, there may be reduced selection for plasmid-mediated resistance). For example, macrolides are more commonly used to treat Gram-positive infections, but less widely used for Gram-negatives due to intrinsic resistance^36^. Accordingly, in our adjusted analysis, Firmicutes plasmids from major clinically relevant species (*Staphylococcus aureus* and *Enterococcus faecium*) carried macrolide ARGs more frequently than Enterobacteriaceae plasmids. However, in less clinically relevant Firmicutes species (e.g. *Bacillus thuringiensis*) macrolide resistance was carried less frequently vs Enterobacteriaceae plasmids, presumably because of reduced selection pressure in non-clinical settings. The finding that overall, major ARGs were more commonly encoded by Enterobacteriaceae plasmids is likely to reflect generally higher antibiotic selection pressure due to the clinical importance of Enterobacteriaceae. We expected to find associations between geography and ARG carriage, given geographic variation in antibiotic prescribing practice. Notably, EU bans on antibiotics for livestock growth promotion were introduced from 1970s (tetracycline, penicillin, streptomycin) with a complete ban in 2006, whereas the US and China were slower to introduce strict controls^37^. Collignon et al. found geographic variation in antibiotic resistance prevalence can also be attributed to socioeconomic factors (e.g. lower GDP)^38^. However, there were no strong differences in ARG carriage across countries. Insufficient variation in antibiotic usage across the income categories analysed may explain this (we were unable to model low-income countries, while lower-middle and upper-middle income countries were combined). Alternatively, geographic sample collection biases (determined by research funding and interests) could mask underlying geographic patterns of ARG carriage.

Analysis of plasmid genetic factors indicated that more recently acquired ARG types (e.g. colistin) were generally less likely to be found on the same plasmid with other ARG types, or with virulence genes. In contrast, the earliest acquired ARG types (aminoglycoside and sulphonamide) were found to frequently co-occur with each other, and other ARG types. Previous studies have shown strong co-occurrence between aminoglycoside and sulphonamide resistance^39^; in our study, we were able to contextualise our findings using the inferred timeline of plasmid ARG acquisition. A plausible interpretation is that over time, plasmid-mediated ARGs tend to accumulate and spread across different plasmid genetic contexts, co-associating with other ARG types, and other adaptive genes, in the process—colistin resistance genes have had less time to co-associate than earlier acquired ARGs. Once adaptive traits become linked on a plasmid, they may become entrenched through co-selection, even if direct selection is reduced^19^. Therefore, novel forms of plasmid-mediated resistance may present an increasing threat to human/animal health. For example, according to the inferred genetic trajectory described above, colistin and carbapenem resistance may increasingly be found in combination with virulence genes in future. However, the pattern could at least in part be explained by sampling bias. For example, identification of more recently acquired ARG types may prompt more widespread screening of non-virulent/environmental strains, due to heightened clinical/veterinary interest. Besides the higher-level patterns across ARG types described above, the strong positive association between integron presence and trimethoprim carriage stood out; this fits with existing literature, reporting that key trimethoprim resistance genes—dihydrofolate reductase genes *dfrA* and *dfrB*—are commonly located on integrons^40^.

A recent study by Che et al. found positive associations between many ARG types and conjugative plasmids^25^, but they did not control for potential confounders such as plasmid size; in our adjusted analysis, most positive associations were attenuated, although positive associations between carbapenem resistance and conjugation remained. This indicates potential for an enhanced role for horizontal transmission in dissemination of carbapenem resistance. We also found positive associations between carbapenem resistance and insertion sequences, aligning with literature reports that major carbapenemase ARGs are associated with insertion sequences and transposons^41,42^ (although our analysis did not distinguish insertion sequences from composite transposons). The positive association between small plasmids (< 10 kb) and quinolone resistance was investigated at the gene-level; co-association of *qnrB19* with Col4401 plasmids was previously highlighted in a literature review^1^. ARG carriage was generally more common among plasmids carrying one or more detected replicon(s) (i.e. single- or multi-replicon plasmids) vs untyped plasmids. This may partly reflect in silico plasmid replicon detection methods, which were developed using clinical reference plasmids (which are in turn likely to be enriched with ARGs)^7^.

A strength of our study lies in elucidating multiple factors associated with ARG carriage, on a broad-scale, and interpreting results across ARG type outcomes through an inferred timeline of selection pressure onset. Use of GAM models allowed us to uncover non-linear patterns which may otherwise have been missed. Such patterns include the plateauing increases in frequency of ESBL, carbapenem, and quinolone over time, and the association between small plasmids and quinolone resistance genes. In addition, using phenotypic data to divide the beta-lactam class into clinically relevant sub-classes (ESBL, carbapenem), yielded additional insights, compared with previous studies, which have analysed the beta-lactam class as a whole rather than ESBL/carbapenem sub-classes^24,25^. Another strength was the rigorous methods for curation of plasmids (e.g. excluding duplicate/replicate plasmids) and sample metadata; for samples that could be linked to the Bac*Dive* metadata database, sample metadata was externally validated, revealing systematic errors (collection date/authority date mix-up). A key limitation of our study was the inherent bias of the dataset due to non-systematic sampling (e.g. selective culture of particular clinical pathogens and resistance phenotypes), which potentially biases inferences. Further, our results may not generalise to plasmids with unrepresented/under-sampled abiotic characteristics (e.g. non-anthropogenic isolation sources, low-income countries). We aimed to mitigate sampling bias as far as possible by delimiting independent sampling units and excluding duplicate/replicate plasmids accordingly. Another limitation of our study is that the unit of analysis was restricted to individual plasmids, yet interactions between co-resident plasmids and the chromosome may also be relevant for understanding factors promoting plasmid ARG carriage. Finally, our study took a broad statistical approach to gain generalisable insights, but in-depth investigations were lacking. For example, investigations into individual ARGs were limited since the GAM modelling was conducted using higher-level ARG type outcomes. In addition, apart from analysing macrolide resistance across Firmicutes species, categorical variable factor levels were broad (e.g. Firmicutes, Enterobacteriaceae etc. rather than species; replicon carriage rather than replicon types). The broad approach was partly due to statistical constraints (introducing too many model terms e.g. finer-resolution categories, leads to model overfitting).

Our analyses demonstrated that plasmid traits are often structured (unevenly distributed), so disentangling their individual effects through observational (non-experimental) studies is challenging. For example, biocide/metal resistance, integron presence, and multi-resistance (> 1 other ARG types encoded) were positively co-associated, and there appeared to be confounding effects between these variables, such that positive unadjusted effects on ARG carriage were attenuated in adjusted analysis. Nevertheless, in this discussion we have highlighted findings where the overall direction of effects remained the same in both unadjusted and adjusted analyses—where this is the case, we can be particularly confident of a robust biological signal.

In summary, we found robust (unadjusted and adjusted) signals of association between plasmid ARG carriage and collection date, human/livestock isolation source, bacterial host taxonomy, virulence gene carriage, and the number of other ARG types encoded. Overall, our findings suggest that following acquisition, plasmid ARGs accumulate under antibiotic selection pressure; over time they also tend to associate with other adaptive genes (virulence genes, ARGs of other types), potentially re-enforcing plasmid ARG carriage through co-selection. Other robust signals of association were the positive associations between integron presence and trimethoprim resistance, and between carbapenem resistance and conjugative plasmids/plasmids with higher density of insertions sequences. In future, global systematic genomic surveillance programmes could provide up-to-date insights into resistance plasmid epidemiology which would be more reliable (less affected by sampling bias)^43,44^. Meanwhile, bioinformatic/statistical analysis of public sequence databases remains valuable for summarising and enhancing knowledge of plasmid-mediated resistance.

## Methods

### Data sources and curation

Complete plasmid genomes and linked metadata were retrieved from NCBI Nucleotide and BioSample databases on 1^st^ May 2019, using in-house tools (https://github.com/AlexOrlek/bacterialBercow; https://github.com/AlexOrlek/getNCBImetadata). Following existing methods^7^, automated exclusion of non-plasmid/incomplete plasmid sequences was based on accession title text analysis and detection of chromosomal housekeeping genes. Identical plasmids were deduplicated; a subset were manually examined to understand redundancies. Hierarchical taxonomic metadata (phylum to species) was derived from taxids, and BioSample metadata was retrieved from canonical attribute name fields (*geo_loc_name, lat_lon, collection_date, isolation_source*) (Supplementary Methods 1.1 and 1.2.1–1.2.3). Location names (*geo_loc_name*) were geocoded using Google Place Autocomplete, accessed via the googleway R package^45^. Internal consistency between the geocoded coordinates and coordinates provided in the *lat_lon* field (where available) was assessed, and discrepancies resolved manually by referring to source literature (Supplementary Methods 1.2.2).

BioSample (“sample” for brevity) metadata was used to inform subsequent plasmid curation steps. Plasmids linked to lab/commercial samples or from samples containing putative vector plasmid(s) were excluded (Supplementary Methods 1.2.4). Plasmids from culture collection samples were deemed to warrant enhanced curation due to redundancy (as culture collection isolates are shared among different researchers and different culture collections) and metadata quality issues. Firstly, culture collection samples were identified by regex searching the designated *culture_collection* field, and other potentially relevant fields (e.g. *strain, sample_name*). Then, plasmids from culture collection samples with identical/synonymous identifiers were deduplicated; furthermore, metadata additions/corrections were implemented according to metadata in Bac*Dive*—a curated metadata database derived from primary culture collection databases^46^; additions/corrections were supported by referring to source literature (Supplementary Methods 1.2.5).

Following de-duplication, we aimed to exclude replicate plasmids (genetically similar plasmids sharing sample metadata), thereby mitigating bias from uneven sampling intensity across sequencing projects^47^. Links between similar plasmids were identified using all-vs-all mash search^48^ (mash distance < 0.1) followed by pairwise BLASTN comparisons (> 95% nucleotide identity, > 50% mean coverage breadth). Pairwise links were retained if metadata (country, collection date, species, BioSample identifier, GenBank BioProject identifier) suggested similar plasmids were non-independent sampling units (i.e. replicates). A clustered network was constructed from retained links (edge-weighted by nucleotide identity); plasmids were filtered by selecting a single representative per cluster (Supplementary Methods 1.2.6, Supplementary Fig. 1). Note that our approach will not simply select one representative per plasmid type (genomic cluster) as previous authors have done^41^; instead, by using metadata (including BioProject) to delimit replicates, genomic cluster representative plasmids should be selected per sequencing project, in order to mitigate bias, while still retaining differences in frequency between plasmid types.

### Plasmid sequence annotation

Plasmid replicons and ARGs were identified using BLASTN searches of PlasmidFinder and ResFinder databases, respectively^49,50^. Using the ResFinder phenotypes.txt file (mapping genes to resistance phenotypes), detected beta-lactam resistance genes were divided into carbapenem and extended-spectrum beta-lactamase (ESBL) resistance gene types. Antibacterial biocide/metal resistance genes and virulence genes were detected by BLASTX queries against experimentally validated proteins of the BacMet and VFDB databases, respectively^51,52^. Conjugative systems were detected using the CONJscan module of MacSyFinder. Complete conjugative systems were identified according to gene content and inter-gene distance stipulations^8^. Integrons and insertion sequences were detected using IntegronFinder and ISEScan, respectively^53,54^ (Supplementary Methods 1.3).

### Statistical analysis

#### Exploratory analysis of plasmid ARGs

The spatiotemporal epidemiology of resistance plasmids was visualised using Microreact (https://microreact.org/). All statistical analysis was performed using R (v3.6.2). The co-occurrence of ARG types was explored based on ARG type presence/absence in the curated plasmid dataset. For each pairwise ARG type combination, co-occurrence was determined using the Jaccard index and overlap coefficient similarity metrics, which provide different perspectives on set similarity (Supplementary Fig. 2). The Jaccard index measures the intersection (plasmids encoding both ARG types) divided by the union (plasmids encoding one or both ARG types); the overlap coefficient measures the intersection divided by the smaller set (i.e. unlike for Jaccard index, differences in set size are not penalised, provided the smaller set is contained within the larger set)^55,56^. Plasmids with earlier collection dates, preceding the emergence of more recently acquired ARG types, may have biased co-occurrence patterns (towards co-occurrence of earlier acquired ARG types). Therefore, we also re-analysed co-occurrence patterns after restricting to a dataset of plasmids with collection dates from 2016 to 2019 (such that all ARG types would be represented throughout the study period). From pairwise co-occurrence analysis, all ARG types were found to have distinct patterns of presence/absence across plasmids in the dataset, and we therefore proceeded in modelling each ARG type separately, as described below.

#### GAM modelling and model checking

Associations between explanatory variables and plasmid ARG carriage (binary outcome) were investigated with logistic Generalised Additive Models (GAMs) using the mgcv package^57^. Separate GAMs were built for 10 ARG types: aminoglycoside, sulphonamide, tetracycline, phenicol, macrolide, trimethoprim, ESBL, carbapenem, quinolone, colistin. These were the most frequent ARG types in the dataset; other ARG types were excluded from analysis due to low frequency (rifampicin, fosfomycin, glycopeptide, oxazolidinone, fusidic acid, and nitroimidazole ARGs were encoded by 224, 183, 44, 42, 3, and 1 plasmids, respectively). Explanatory variables were selected based on a priori biological knowledge. Integrons, biocide/metal resistance genes, and virulence genes were binary categorical (presence/absence) (Table 1). Only complete integrons (encoding an integrase and ≥ 1 *attC* site) were included as present. Conjugative system, replicon carriage, host taxonomy, geographic location, and isolation source were non-binary categorical; low frequency factor levels were collapsed based on biological plausibility. For example, the > 500 replicon type “haplotypes” were collapsed to a 3-level replicon carriage variable (untyped, single-replicon, multi-replicon) (Supplementary Methods 1.4 Supplementary Fig. 3). Plasmid size (kb), insertion sequence density (number per 10 kb), number of other ARG types, and collection date (collection year), were continuous variables. The number of other ARG types was determined per ARG type model based on the number of other encoded ARG types represented, irrespective of the number of individual ARGs. Outlier influence was reduced by “winsorising”^58^ (limiting extreme values in long-tailed distributions to threshold values) at the 5th/95th percentiles, on the left tail (collection date); right tail (insertion sequence density, number of other ARG types); both tails (plasmid size). Plasmid size was log_10_-transformed and centred on 10 kb. During modelling, collection date was expressed as years since the reference (5^th^ percentile) collection year of 1994. Missing collection dates (n = 7768, 55%) were singly imputed using accession ‘create dates’. However, there was weak correlation (Pearson’s *r* = 0.314) between accession create dates and collection dates for plasmids with non-missing collection dates (Supplementary Methods 1.4; Supplementary Figs. 4, 5). Therefore, we also re-analysed the univariable association between ARG carriage and collection date, after omitting the 55% plasmids with imputed collection dates. Multivariable GAM models, including all categorical and continuous explanatory variables, were constructed for each ARG type outcome (Supplementary Methods 1.6). Continuous variables were modelled using smooth terms, restricted to a maximum of 5 basis functions to avoid over-fitting. A point constraint was applied to smooths (effect on log-odds = 0 at x = 0), to facilitate comparison between models.

Interrelationships among explanatory variables and potential collinearity were investigated using Spearman’s correlation (continuous–continuous, continuous–binary); Cramér's V statistic (categorical–categorical); and Kruskal–Wallis eta statistic (continuous–categorical). A threshold correlation statistic of 0.7 was used to determine whether explanatory variables should be discarded/transformed prior to modelling^59^. We had initially considered modelling insertion sequences as a count, but correlation between number of insertion sequences and plasmid size exceeded the 0.7 threshold (Spearman's ρ = 0.74); after expressing as insertion sequence density per 10 kb, all association statistics between explanatory variables were < 0.7 (Supplementary Data 2a. Model checking was conducted using gam.check(). All GAM models converged. Basis dimensionality checking indicated non-random patterns in the residuals for log_10_ plasmid size smooths across all models, and for insertion sequence density smooths in 5/10 models. This was not resolved by increasing the basis dimensionality. The terms were retained, but the smooths should be interpreted cautiously. Model R^2^ values ranged from 0.26 (ESBL) to 0.77 (sulphonamide). Model outputs were generated using anova.gam() and summary.gam(), which implement Wald tests^57^, that we assessed against a Bonferroni-adjusted alpha = 0.00024 (210 comparisons: 17 parametric factor levels and 4 smooth terms per model, across 10 models). The same model was fitted to each ARG outcome, ensuring that the same potential confounders were adjusted for across models. Sensitivity analyses considered alternative models, fitted with various terms removed to investigate confounding.

#### Investigating antibiotic selection pressure using proxy variables and a timeline of plasmid ARG acquisition

Antibiotic selection pressure was a priori expected to be associated with plasmid ARG carriage (see Introduction); however, direct assessment of antibiotic selection pressure was not possible, since plasmid-level antibiotic exposure data was lacking. Instead, the influence of antibiotic selection pressure was interpreted through proxy variables—host taxonomy, isolation source, and collection date—which were selected based on literature support. Specifically, existing literature describes variation in intrinsic resistance to different ARG types across bacterial taxa, which presumably affects selection for plasmid-mediated resistance (i.e. stronger intrinsic resistance is likely to reduce selection pressure for plasmid-mediated resistance). Further, antibiotic prescribing practices in humans vs livestock differ (e.g. carbapenems are primarily reserved for clinical rather than veterinary usage)^34,35^. Finally, selection pressure for ARG types should vary by collection date, reflecting the timeline of antibiotic introductions, plasmid ARG acquisition, and hence, onset of selection pressure. A structured literature search was conducted to determine first recorded date of plasmid-mediated resistance for each antibiotic ARG type, so that results could be interpreted in relation to presumed onset of antibiotic selection pressure (Supplementary Methods 1.5).

## Supplementary Information

Supplementary Information.

## Data Availability

Curated plasmid sequences (14,143 plasmids) are available as a FASTA file (https://doi.org/10.6084/m9.figshare.19438994). Supplementary Data 1 provides the tabular plasmid dataset used for statistical analysis (including plasmid accession numbers) (https://github.com/AlexOrlek/PlasmidAMRCarriage_paper/blob/main/data/Data_S1.xlsx). Code for statistical analysis is available: https://github.com/AlexOrlek/PlasmidARGCarriage.
