# Supplementary material for: Factors associated with plasmid antibiotic resistance gene carriage revealed using large-scale multivariable analysis
Source: Sci Rep. 2023 Feb 13;13:2500. doi: 10.1038/s41598-023-29530-y (PMC9925765; doi:10.1038/s41598-023-29530-y)
Supplement: Supplementary file 1 — Supplementary Information. [file 41598_2023_29530_MOESM1_ESM.pdf]

Supplementary Material

Factors associated with plasmid antibiotic resistance gene carriage revealed using large-scale multivariable analysis

Alex Orlek DPhil[1,2,3*], Muna F. Anjum PhD[4], Alison E. Mather PhD[5,6], Nicole Stoesser DPhil[2,7], A. Sarah Walker PhD[2,3,7]

[1]HCAI, Fungal, AMR, AMU & Sepsis Division, UK Health Security Agency, London, UK

[2]Nuffield Department of Medicine, University of Oxford, Oxford, UK

[3]NIHR Health Protection Research Unit in Healthcare Associated Infections and Antimicrobial Resistance, University of Oxford, Oxford, UK.

[4]Department of Bacteriology, Animal and Plant Health Agency, Weybridge, Addlestone, UK

[5]Quadram Institute Bioscience, Norwich, UK

[6]University of East Anglia, Norwich, UK

[7]NIHR Oxford Biomedical Research Centre (BRC), University of Oxford, Oxford, UK

[*] **Correspondence**:
Alex Orlek
Alex.Orlek@ukhsa.gov.uk

Contents

1 Methods 3
1.1 Data sources 3
1.2 Data curation 3
1.2.1 Determining valid location names and latitude/longitude coordinates 3
1.2.2 Geocoding methods 4
1.2.3 Curation of collection date and host/isolation source metadata 6
1.2.4 Identification and exclusion of laboratory and commercial samples 9
1.2.5 Identification and curation of culture collection samples 10
1.2.6 Filtering putative replicate plasmids based on pairwise sequence similarity and metadata sharing 11
1.3 Plasmid sequence annotation 12
1.4 Exploratory analysis of categorical and continuous variables 12
1.5 Literature search strategy for determining dates of first recorded plasmid antibiotic resistance gene (ARG), for each ARG type 19
1.6 GAM modelling methods 19
2 Results 20
2.1 Results of manual examination of identical plasmid accessions 20
2.2 Results of data retrieval, curation, and cleaning 25
2.3 Results of statistical analysis (exploratory, unadjusted, and adjusted analysis) 27
2.4 Model checking 29
2.5 Categorical explanatory variable effect plots 29
2.6 Continuous explanatory variable effect plots 37
2.7 Investigation of confounding 42
2.7.1 Host taxonomy 42
2.7.2 Biocide/metal resistance gene presence, integron presence, number of other ARG types 45
2.7.3 Conjugative system 49
3 References 53

1 Methods

1.1 Data sources

Complete bacterial plasmids were retrieved from NCBI Nucleotide (Refseq and Genbank) on 1st May 2019 using in-house software (https://github.com/AlexOrlek/bacterialBercow) [1]. This software pipeline conducts initial curation, including automated deduplication of identical plasmids; a list of deduplicated identical plasmid accessions is recorded. Key metadata was also retrieved using the pipeline: BioSample accession ids, BioProject accession ids, submitter contact name, submitter affiliation. In addition, for Refseq accessions, cognate GenBank accession ids and GenBank BioProject accessions ids were retrieved. This metadata was used to identify a subset of identical plasmids for manual examination (see below).

Additional BioSample metadata was subsequently retrieved from NCBI BioSample using in-house software (https://github.com/AlexOrlek/getNCBImetadata) [2]. The following canonical ('harmonized') [3] BioSample attribute names were specified for retrieval:

collection_date, host, lab_host, isolation_source, substrate, tissue, host_body_habitat, host_body_product, host_description, host_disease, host_substrate, host_tissue_sampled, plant_body_site, plant_product, sample_name, strain, sample_type, geo_loc_name, lat_lon, env_broad_scale, env_local_scale, env_medium, env_package, project_name, culture_collection, biomaterial_provider, ref_biomaterial, specimen_voucher, reference_material, derived_from, description

Hierarchical taxonomic metadata (phylum to species) was derived from taxids using the ete3 package [4].

1.2 Data curation

1.2.1 Determining valid location names and latitude/longitude coordinates

Geographic location names and latitude/longitude coordinates were retrieved from the *geo_loc_name* and *lat_lon* fields, respectively. Firstly, case-insensitive matching to the following list of words was used to exclude invalid locations (*geo_loc_name* field) and latitude/longitude coordinates (*lat_lon* field):

'missing', '-', 'n/a', 'unknown', 'not collected', 'not applicable', 'na', 'none', 'not available', 'not determined', 'not recorded', 'n.a.'

In addition, the following Python regex was used to confirm that *lat_lon* coordinates were correctly formatted:

Code snippet 1. Python regex to validate *lat_lon* coordinates.

```
regexObj=re.compile(r'\d+\.?\d* [NS] \d+\.?\d* [WE]')

match=re.search(regexObj,latlon)
```

1.2.2 Geocoding methods

Note, geocoding methods were informed by an initial intention to achieve high geospatial resolution; however, BioSample geographic location names are provided at country-level resolution, so simply identifying countries, and reverse-geocoding to broader categories where necessary would have been sufficient for fitting the GAM models.

Unique valid location names were used to retrieve geocoding place predictions from the Google Place Autocomplete service, accessed via the googleway package [5] (googleway argument: place_type='geocode'); this returns up to five place predictions, ordered by relevance. Associated geocoded latitude and longitude coordinates were subsequently retrieved through a Google Place Details request. If the top hit geocoded place name description was an exact and unambiguous match to the BioSample place name (see below), the geocoded place prediction and associated coordinates were accepted; otherwise, manual geocoding was conducted.

Determining whether top hit geocoded place name predictions from Google Place Autocomplete match exactly to BioSample geographic location names:

Matching between top hit geocoded place name descriptions and BioSample place names was assessed by searching for matching substrings using the stringr R package (Code snippet 2). An exact match occurred when there were no unmatched substrings in either the geocoded place description or BioSample place name.

Code snippet 2. R code to identify exact matches between top hit geocoded place name descriptions and BioSample place names.

```
library(stringr)

##Example data

BioSamplePlace<-"Myanmar:Yangon"

GooglePlaceDescription<-"Yangon, Myanmar" #predicted top hit

##Code snippet

BioSamplePlace<-tolower(BioSamplePlace)

GooglePlaceWords<-
unlist(lapply(strsplit(GooglePlaceDescription,', ',fixed=T), function(x) x=tolower(x)))

GooglePlaceNchar<-sum(sapply(GooglePlaceWords,nchar))


#for each Word in GooglePlaceWords, check for match in BioSamplePlace; if there is a matchi
ng substring, add to GoogleNCharMatched counter and remove matching substring from BioSampl
ePlace

GooglePlaceNCharMatched<-0

for (Word in GooglePlaceWords) {

  NCharMatched<-nchar(str_extract(BioSamplePlace,Word))

  if (is.na(NCharMatched)) {

    NCharMatched<-0
```

```
  }
  GooglePlaceNCharMatched<-GooglePlaceNCharMatched+NCharMatched
  BioSamplePlace<-str_remove(BioSamplePlace,Word)
}
BioSamplePlace<-gsub("[[:punct:]]", "", BioSamplePlace)
BioSamplePlace<-gsub("[[:space:]]", "", BioSamplePlace)
NCharUnmatched_BioSamplePlace<-nchar(BioSamplePlace)
NCharUnmatched_GooglePlace<-GooglePlaceNChar-GooglePlaceNCharMatched
#In this case, there are 0 unmatched characters for both GooglePlace and BioSamplePlace...
i.e. exact match
```

Determining whether top hit place name predictions are ambiguous:

If an exact match was confirmed (see above), the place prediction was not automatically accepted unless the place name was unambiguous. Specifically, if the top hit place name from the "main_text" field [6] was identical to that of other place prediction(s), and these prediction(s) were also equal or higher in the place type hierarchy (defined in the table below), the top hit was flagged as ambiguous.

Supplementary Table 1. Hierarchy of Google Place types from country through to precise local-level locations

Place type(s)	Hierarchical index	Hierarchical category
political<br>colloquial_area<br>geocode	NA*	vague location
country	1	hierarchical location
administrative_area<br>administrative_area_level_1	2	hierarchical location
administrative_area_level_2	3	hierarchical location
administrative_area_level_3	4	hierarchical location
administrative_area_level_4	5	hierarchical location
administrative_area_level_5	6	hierarchical location
postal_town<br>locality	7	hierarchical location
sublocality<br>sublocality_level_1	8	hierarchical location
sublocality_level_2	9	hierarchical location
sublocality_level_3	10	hierarchical location
sublocality_level_4	11	hierarchical location

sublocality_level_5	12	hierarchical location
neighborhood	13	hierarchical location
premise<br>subpremise<br>postal_code<br>natural_feature<br>airport<br>park<br>point_of_interest<br>street_address<br>route<br>intersection	14	local-level location

Place types are Google Place geocoding place types:

(https://developers.google.com/maps/documentation/geocoding/overview#Types). The hierarchical ordering of these place types was determined by this author. Only the first place type of a compound place type was used to assess place type hierarchy (e.g. only "locality" for a compound place type such as "locality, political, geocode").

*If the top hit autocomplete place prediction is a "vague location" and has the same place name (main_text) as another hit, it is flagged as ambiguous, unless all other hits are local-level locations

Following geocoding, for BioSample accessions with available metadata, internal consistency between geocoded coordinates and *lat_lon* field coordinates was assessed, and discrepancies manually resolved (referring to source literature). For downstream analysis, curated latitude/longitude coordinates were reverse geocoded at the level of country and higher-level groupings: European Union (EU) countries (including the UK); and World Bank income groupings [7].

1.2.3 Curation of collection date and host/isolation source metadata

Valid collection dates were extracted from the *collection_date* field using regexes given in Code snippet 3.

Code snippet 3. Python regexes used to extract valid collection dates.

```
#Major collection date formats                        #Example matches

format1=re.compile(r'^\d\d-[A-Z|a-z]{3,9}-\d\d\d\d$')  #05-Jun-2020 or 22-April-2020

format2=re.compile(r'^[A-Z|a-z]{3,9}-\d\d\d\d$')       #Apr-2020 or June-2020

format3=re.compile(r'^\d\d\d\d-\d\d-\d\d$')            #2020-04-22

format4=re.compile(r'^\d\d\d\d-\d\d$')                 #2020-04

format5=re.compile(r'^\d\d\d\d$')                      #2020

format6=re.compile(r'^\d{1,2}-[A-Z|a-z]{3}-\d\d$')     #5-Jun-20

format7=re.compile(r'^[A-Z|a-z]{3}-\d\d$')             #Apr-20

format8=re.compile(r'^\d\d\d\d/\d\d\d\d$')             #2020/2021
```

Regexes were also used to curate host/isolation source metadata (Code snippets 4–8) from the following fields: *host*, *isolation_source*, *host_description*, *env_broad_scale*, *env_local_scale*, *env_medium*, *description*. Matches were manually curated. Code snippet 5 covers globally important livestock species (cattle, pigs, chicken, sheep, goats, ducks, turkeys) [8,9]. Matches to processed livestock products (e.g. dairy, ham) were manually excluded. Code snippet 6 covers major aquaculture species [10]. Code snippet 7 represents agricultural crop species (compiled from FAOSTAT; http://www.fao.org/faostat/en/#data/), bacterial pathogens of crops [11], and agriculture-related words (note that 'fish farm' was excluded from matches to 'farm'). Matches to wild plants or processed agricultural produce (e.g. sugar) were manually excluded. Code snippet 8 was used to identify sewage samples; as far as possible (using available metadata) matches were restricted to human sewage samples; if metadata indicated industrial wastewater or livestock/agricultural wastewater, matches were excluded.

Code snippet 4. Python regex used to identify human samples.

```
human_regex=re.compile(r'\bhomo\b|h.? sapiens|homosapiens|human|patient|clinical|hospital|\
bman\b|woman|adult|child|infant|neonate|person',re.IGNORECASE)
```

Code snippet 5. Python regexes used to identify livestock samples.

```
cow_regex=re.compile(r'\bbos\b|b.? taurus|\bcows?\b|cattle|bovid|bovine|calf|calves|calving
|bull|bullock|heifer|springer|steer|veal|udder|mastitis|beef|steak|brisket|sirloin|t-
bone',re.IGNORECASE)

pig_regex=re.compile(r'\bsus\b|s.? scrofa|\bpigs?\b|swine|\bhogs?\b|piglet|sow|barrow|\bgil
ts?\b|shoat|porcine|pork|trotter',re.IGNORECASE)

chicken_regex=re.compile(r'gallus|chicken|rooster|\bcocks?\b|\bhens?\b|pullet|broiler|chook
|chick|poultry|\beggs?\b',re.IGNORECASE)

sheep_regex=re.compile(r'\bovis\b|o.? aries|sheep|lamb|\bewes?\b|\bram\b|hogget|\bovine\b',
re.IGNORECASE)

goat_regex=re.compile(r'capra|aegagrus|\bhircus\b|goat|\bkids?\b|caprine',re.IGNORECASE)

duck_regex=re.compile(r'\banas\b|platyrhynchos|duck',re.IGNORECASE)

turkey_regex=re.compile(r'meleagris|gallopavo|turkey',re.IGNORECASE)
```

Code snippet 6. Python regex used to identify aquaculture samples.

```
aquaculture_regex=re.compile(r'fish farm|aquaculture|pisciculture|mariculture|\bcarps?\b|Ct
enopharyngodon|C.? idellus|Hypophthalmichthys|H.? molitrix|H.? nobilis|Cyprinus carpio|C.?
carpio|tilapia|Oreochromis|O.? niloticus|Carassius|\bCatla\b|\bsalmon\b|\bSalmo\b|\broho\b|
\brohu\b|\brui\b|Labeo rohita|L.? rohita|catfish|Pangasius|Milkfish|\bChanos\b|\bClarias\b|
Wuchang bream|Megalobrama amblycephala|M.? amblycephala|\btrouts?\b|Oncorhynchus mykiss|O.?
 mykiss|Mylopharyngodon piceus|M.? piceus|\bSnakehead\b|Channa argus|C.? argus|\bshrimps?\b
|Penaeus vannamei|P.? vannamei|crawfish|crayfish|Procambarus clarki|P. clarkii|Chinese mitt
en crab|Eriocheir sinensis|E.? sinensis|\bprawns?\b|Penaeus monodon|P.? monodon|Macrobrachi
```

```
um|\boysters?\b|Crassostrea|Japanese carpet shell|Ruditapes|\bScallops?\b|Pectinidae|\bmuss
els?\b|Mytilidae|Constricted tagelus|Sinonovacula constricta|S.? constricta|\bcockles?\b|An
adara granosa|A.? granosa|Chinese softshell turtle|Trionyx sinensis|T.? sinensis|sea cucumb
er|Apostichopus japonicus|A.? japonicus',re.IGNORECASE)
```

Code snippet 7. Python regex used to identify (non-livestock) agriculture samples.

```
agriculture_regex=re.compile(r'agricultur|horticultur|floricultur|viticultur|\bfarms?\b|\bc
rops?\b|\bpastures?\b|\bpaddy\b|\bpaddies\b|greenhouse|\bgrove\b|orchard|plantation|vineyar
d|\bleaf\b|leaves|\bstems?\b|leaf|leaves|phyllosphere|\broots?\b|\bseeds?\b|\bflowers?\b|\b
tubers?\b|Fruit|\bBeans?\b|Berry|Berries|Peanut|\bNuts?\b|\bSeeds?\b|\bAgave\b|Almond|\bAni
se\b|badian|fennel|coriander|\bApples?\b|Apricot|Areca|Artichoke|Asparagus|Avocado|Bambara|
Banana|Barley|Bast ?fibre|Buckwheat|Cabbage|Canary seed|\bCarobs?\b|Turnip|Carrot|Cassava|C
astor|Cauliflower|Broccoli|Cereal|Cherries|Cherry|Chestnut|Chick ?pea|Chicory|Chillies|Chil
li|\bDates?\b|Date palm|Eggplant|Aubergine|\bFigs?\b|Flax|\bFonio\b|Citrus|Garlic|Ginger|Gr
ain|Pomelos|Shaddock|Grapes?|Hemp|Hempseed|\bHops?\b|Jojoba|juniper|Jute|Kapok|Karite|\bShe
a\b|sheabutter|Kiwi|Kola|\bLeeks?\b|Lemon|\bLimes?\b|Lentil|Lettuce|Chicory|Linseed|Lupin|M
aize|Mango|mangosteen|guava|Manila fibre|\babaca\b|\bMate\b|Melon|Millet|Mustard|Nutmeg|\bm
ace\b|cardamom|\bOats?\b|Oil ?palm|Palm ?oil|Oil ?seed|Pepper|Cinnamon|Clove|Cocoa|Coconut|
Coffee|Coir|Cow ?pea|Cucumber|Gherkin|Currant|Okra|Olive|Onion|shallot|Orange|Papaya|Pawpaw
|Peach|Nectarine|\bPears?\b|\bPeas?\b|Peppermint|Persimmon|Pineapple|Pistachio|Plantain|\bP
lums?\b|sloe|Poppy|Potato|\bPulses?\b|Pumpkin|raisin|\bsquash\b|\bsquashes\bgourd|Pyrethrum
|Quince|Quinoa|Ramie|Rapeseed|canola|colza|\bRice\b|Rubber|\bRye\b|Safflower|cotton|\bSago\
b|Sesame|Sisal|Sorghum|Soybean|Spinach|Sugarbeet|Sugarcane|\bSugar\b|Sunflower|Tallowtree|T
angerine|mandarin|clementine|satsuma|\bTaro\b|cocoyam|\bTea\b|Tobacco|Tomato|Triticale|\bTu
ng\b|Vanilla|Vegetable|Vetch|Walnut|Watermelon|Wheat|Yam|Yautia|goji|Prunus|Pimpinella anis
um|Illicium verum|Foeniculum vulgare|Coriandrum sativum|Malus domestica|Areca catechu|Cynar
a cardunculus|Asparagus officinalis|Persea americana|Vigna subterranea|Musa|Hordeum vulgare
|Vaccinium corymbosum|Bertholletia excelsa|Vicia|Fagopyrum esculentum|Brassica|Phalaris can
ariensis|Ceratonia siliqua|Daucus carota|Anacardium occidentale|Manihot esculenta|Ricinus c
ommunis|Castanea|Cicer arietinum|Cichorium intybus|Capsicum |Cinnamomum verum|Syzygium arom
aticum|Theobroma cacao|Cocos nucifera|Coffea|Vigna unguiculata|Vaccinium Oxycoccus|Cucumis 
sativus|\bRibes\b|Phoenix dactylifera|Solanum melongena|\bFicus\b|Linum usitatissimum|\bAll
ium\b|Zingiber officinale|\bVitis\b|\bArachis\b|Cannabis|Humulus lupulus|Simmondsia chinens
is|Corchorus capsularis|Ceiba pentandra|Vitellaria paradoxa|Actinidia|\bCola\b|juniperus co
mmunis|Lens culinaris|Lens esculenta|Lactuca sativa|Cichorium intybus|Linum usitatissimum|L
upinus|Zea mays|Mangifera|Garcinia mangostana|Metroxylon sagu|Psidium guajava|Ilex paraguar
iensis|Benincasa|Citrullus|Cucumis|Panicum|Myristica fragrans|Elettaria cardamomum|Amomum|A
vena sativa|Elaeis|Attalea maripa|Abelmoschus esculentus|Olea|\bCarica\b|\bPyrus\b|Pisum sa
tivum|Piper|\bMentha\b|Diospyros|Cajanus cajan|Ananas|Pistacia|Papaver somniferum|Solanum t
uberosum|Cucurbita|Tanacetum cinerariaefolium|Cydonia|Boehmeria nivea|\bRubus\b|Oryza|Hevea
 brasiliensis|Secale cereale|Carthamus tinctorium|Gossypium|Sesamum|Agave sisalana|Glycine 
max|Spinacia oleracea|Fragaria|Phaseolus vulgaris|Beta vulgaris|Saccharum|Helianthus annuus
|Ipomoea batatas|Triadica sebifera|Colocasia esculenta|Camellia sinensis|Nicotiana|Solanum 
lycopersicum|Triticosecale|Vernicia fordii|Juglans|Citrullus lanatus|Triticum aestivum|Dios
corea|Xanthosoma|Lycium barbarum|Pseudomonas syringae|Ralstonia solanacearum|Agrobacterium 
tumefaciens|Xanthomonas oryzae|Xanthomonas campestris|Xanthomonas axonopodis|Erwinia amylov
```

```
ora|Xylella fastidiosa|Dickeya dadantii|Dickeya solani|Pectobacterium carotovorum|Pectobact
erium atrosepticum',re.IGNORECASE)
```

Code snippet 8. Python regex used to identify sewage samples.

```
sewage_regex=re.compile(r'activated sludge|sewage|sewer|waste-
water|waste water|water treatment|effluent',re.IGNORECASE)
```

1.2.4 Identification and exclusion of laboratory and commercial samples

All retrieved attribute fields were searched for the terms "commercial", "biocontrol", "-cide" (e.g. biocide). The *host* and *lab_host* attribute fields were searched for animal/plant model organism names (Code snippet 9). Plant and animal model laboratory organisms were compiled based on literature reports [12–15].

Code snippet 9. Python regex to identify laboratory model organisms.

```
lab_model_regex=re.compile(r'Mus musculus|M.? musculus|mouse|Rattus|R.? norvegicus|\brat\b|
Danio rerio|D.? rerio|zebrafish|Drosophila|D.? melanogaster|fruit fly|Caenorhabditis elegan
s|C.? elegans|nematode|Sepiola atlantica|S.? atlantica|Bobtail squid|Arabidopsis|A.? thalia
na|thale cress|Galleria mellonella|G.? mellonella|greater wax moth|honeycomb moth|Lotus jap
onicus|L.? japonicus|Medicago|M.? trunculata|M.? sativa|barrelclover|alfalfa|Brachypodium|B
.? distachyon|false brome|Oryza|\brice\b|Nicotiana|N.? benthamiana|Glycine|soybean|Triticum
|\bwheat\b|Zea mays|maize|Brassica napus|rapeseed|oilseed rape|Populus|P.? trichocarpa',re.
IGNORECASE)
```

Additional laboratory samples were flagged using the regex below, which was searched against the following fields:

Title (xml element: .//Description/Title)
Comment (xml element: .//Description/Comment/Paragraph)
As well as the following attribute fields: *isolation_source*, *sample_type*, *env_broad_scale*, *env_local_scale*, *env_medium*

Code snippet 10. Python regex to identify laboratory samples.

```
lab_regex=re.compile(r'\blab\b|laboratory',re.IGNORECASE)
```

Additionally, plasmid sequences were megaBLAST queried against the UniVec vector database (https://www.ncbi.nlm.nih.gov/tools/vecscreen/univec/; evalue=1e-8, max_target_seqs=10000; 95% identity and 50% query coverage). Plasmids matching the database were excluded as putative vector plasmids.

1.2.5 Identification and curation of culture collection samples

Culture collection samples were deemed to warrant enhanced curation for the following reasons. Firstly, a given culture collection sample may be sequenced by multiple researchers and shared between collections (accruing different synonymous culture collection identifiers). Secondly, culture collection metadata may be error-prone (e.g. instead of original collection date/location, submitters may provide a later acquisition date/location). To identify culture collection samples, culture collection acronyms and names were compiled from the Word Federation for Culture Collection website (http://www.wfcc.info/ccinfo/collection/by_acronym/; accessed 6th Jun 2019); additional acronyms were compiled from a list produced by the International Journal of Systematic and Evolutionary Microbiology [16]. A regex was used to identify culture collection identifiers in the *culture_collection* attribute field:

Code snippet 11. Python regex to identify culture collection identifiers.

```
#Generating a regex from a list of acronyms ("acronyms_list") #Example matches:

acronym_regex=[r'\b%s.{0,3}[0-9]*\b'%a for a in acronyms]     #ATCC43845 or ATCC : 43845

combined_acronym_regex=re.compile(r'|'.join(acronyms))
```

Where a match was not found using the above approach, other BioSample fields (specified below) were searched using regexes defined in Code snippets 11 and 12; in addition, fuzzy string matching implemented with the Python fuzz module [17] was used to search for culture collection names (e.g. "American Type Culture Collection"). All matches from this step were manually examined.

Sample name identifier (xml element: Id[@db_label="Sample name"])
Title (xml element: .//Description/Title)
Comment (xml element: .//Description/Comment/Paragraph)
As well as the following attribute fields: *isolation_source*, *sample_name*, *strain*, *geo_loc_name*, *project_name*, *ref_biomaterial*, *description*, *reference_material*, *biomaterial_provider*

Code snippet 12. Additional Python regex to identify culture collection identifiers.

```
descriptive_regex=re.compile(r'culture collection|type strain',re.IGNORECASE)
```

Culture collection samples were curated using external metadata from the Bac*Dive* database. This database contains aggregated bacterial strain metadata, derived and curated from primary culture collection databases [18,19]. Consequently, Bac*Dive* should be a reliable source of metadata, which can be used to externally validate NCBI BioSample metadata. Metadata on isolation source, geographic location, and culture collection synonyms was downloaded from Bac*Dive* (https://bacdive.dsmz.de/isolation-sources; accessed 4th Jul 2019). In addition, available collection date metadata was kindly provided by Dr Lorenz Reimer. Bac*Dive* metadata was used to guide manual curation of BioSample metadata (host, isolation source, geographic location, latitude/longitude, collection date); additions and corrections were implemented in accordance with Bac*Dive* metadata, and supported by referring back to original

source literature. Furthermore, culture collection synonym information from Bac*Dive* was used to facilitate manual deduplication of culture collection samples sharing synonymous culture collection identifiers. Deduplication of samples with identical/synonymous culture collection identifiers favoured retention of samples with higher assembly contiguity; plasmids linked to excluded BioSample accessions were excluded from the curated plasmid dataset.

1.2.6 Filtering putative replicate plasmids based on pairwise sequence similarity and metadata sharing

Pairs of plasmids with high sequence similarity were identified using mash (mash distance <0.1) [20] followed by BLASTN comparisons (>95% nucleotide identity, >50% mean coverage breadth; https://github.com/AlexOrlek/ATCG). Then, retention of links between similar plasmids for downstream filtering, was determined by metadata sharing, according to the decision tree below. An igraph network [21] was constructed from retained links (edge-weighted by nucleotide identity); the network of remaining linked plasmids was clustered using the infomap algorithm [22], and one representative plasmid per cluster was selected for inclusion in the final plasmid dataset (along with the plasmids that did not share sequence similarity and metadata with one or more other plasmids, and were therefore not subjected to the described filtering steps).

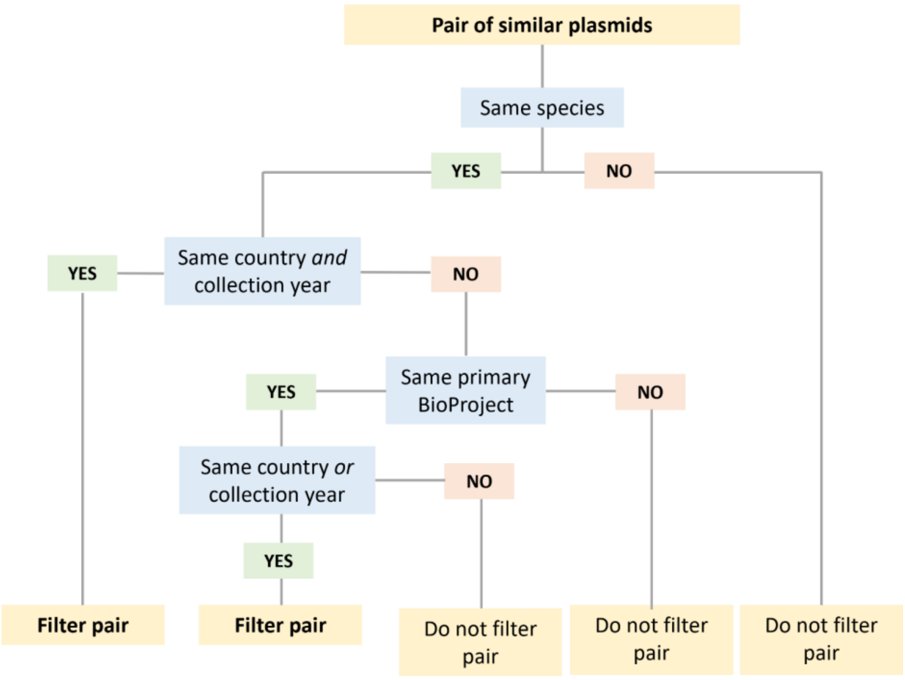

Supplementary Figure 1. Decision tree for filtering similar plasmids with shared metadata; non-independent plasmid pairs (similar plasmids sharing metadata) were filtered using a clustering approach. Note that a species/country/collection year/primary BioProject identifier field was not considered the same if data was missing for both members of the pair (i.e. discounting shared missingness).

1.3 Plasmid sequence annotation

Supplementary Table 2. Summary of plasmid sequence annotation methods.

Plasmid annotation	Annotation method	Notes / software parameters
Plasmid replicons	PlasmidFinder [23] (Enterobacteriaceae and Gram-positive databases retrieved 25th September 2019)	BLASTN (80% sequence identity and 60% replicon sequence coverage breadth thresholds).
Antibiotic resistance genes	ResFinder [24,25] (database retrieved 25th September 2019)	BLASTN (parameter: max_target_seqs=5000; 90% sequence identity and 60% coverage breadth thresholds).<br>The phenotypes.txt file was used to map detected beta-lactam resistance genes to resistance types of interest (ESBL, carbapenem).
Antibacterial biocide/metal resistance genes	BacMet [26] database of experimentally validated proteins (retrieved 30th September 2019)	BLASTX (parameters: evalue=0.001, max_target_seqs=1000; 90% sequence identity and 30 bp alignment length thresholds).
Virulence genes	Virulence Factor Database (VFDB) [27] database of experimentally validated proteins (retrieved 15th October 2019)	BLASTX (parameters: evalue=1e-5, max_target_seqs=5000; 75% identity and 50% coverage thresholds).
Conjugative systems	CONJscan module of MacSyFinder [28] (https://github.com/gem-pasteur/Macsyfinder_models/commits/master/models/Conjugation).	MacSyFinder parameter: *--replicon-topology* circular.<br>Firstly, plasmid protein-coding genes were annotated with Prodigal [29], using "anonymous mode" for smaller plasmids and "normal mode" for larger plasmids (≥100 kb). Plasmid proteomes were searched using the profiles defined previously [28], with an additional profile for the novel MOBM relaxase, downloaded from MOBfamDB [30]. Complete conjugative systems were defined according to previously described gene content and inter-gene distance stipulations [31]. MOB-relaxases and complete conjugative systems were identified by parsing the output files.
Integrons	IntegronFinder (v2.0) [32]	IntegronFinder parameters: *--local-max* (for increased sensitivity), *--circ* (to set replicon topology as circular).
Insertion sequences	ISEScan (v1.7.1) [33]	Default parameters

1.4 Exploratory analysis of categorical and continuous variables

The co-occurrence of ARG types (binary categorical outcome variables) was explored based on ARG type presence/absence in the curated plasmid dataset, using similarity metrics (Jaccard index and overlap coefficient) (Supplementary Figure 2). Regarding explanatory variables, exploratory analysis of categorical variables was used to determine factor level re-coding (Supplementary Tables 3–5; Supplementary Figure 3). Correlation between collection dates

and create dates was assessed to determine validity of imputing collection dates with create dates (Supplementary Figures 4, 5).

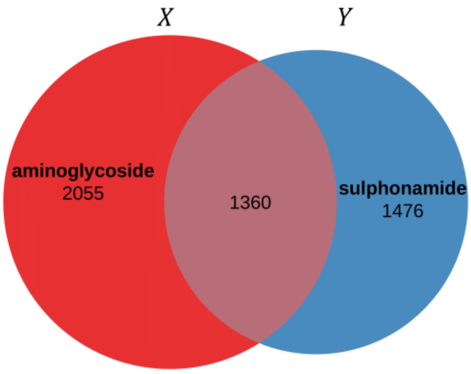

$$jaccard(X,Y) = \frac{|X \cap Y|}{|X \cup Y|} = \frac{|X \cap Y|}{|X| + |Y| - |X \cap Y|} = \frac{1360}{2171}$$

$$overlap(X,Y) = \frac{|X \cap Y|}{\min(|X|,|Y|)} = \frac{1360}{1476}$$

Supplementary Figure 2. Calculation of Jaccard index and overlap coefficient similarity metrics is illustrated using a Venn diagram (an example is shown for co-occurrence between aminoglycoside and sulphonamide ARG types).

Supplementary Table 3. Replicon carriage characteristics of the top 10 most frequent taxa (at species-level) within each factor level of the host taxonomy explanatory variable (based on the dataset of 14143 plasmids).

**Enterobacteriaceae**					
Species	Total plasmids	Replicon carriage			
		Single-replicon	Multi-replicon	Untyped	Typed
Escherichia coli	1943	150	1249	544	1793 (92.3)
Klebsiella pneumoniae	1103	92	645	366	1011 (91.7)
Salmonella enterica	596	60	325	211	536 (89.9)
Enterobacter cloacae	136	15	71	50	121 (89)
Citrobacter freundii	115	14	76	25	101 (87.8)
Shigella sonnei	88	5	83	0	83 (94.3)
Enterobacter hormaechei	85	11	37	37	74 (87.1)
Klebsiella oxytoca	64	13	38	13	51 (79.7)
Klebsiella aerogenes	41	11	24	6	30 (73.2)
Shigella flexneri	40	3	30	7	37 (92.5)
**Proteobacteria (non-Enterobacteriaceae)**					
Species	Total plasmids	Replicon carriage			
		Single-replicon	Multi-replicon	Untyped	Typed
Acinetobacter baumannii	245	245	0	0	0 (0)

Yersinia pestis	116	3	103	10	113 (97.4)
Xanthomonas citri	80	80	0	0	0 (0)
Helicobacter pylori	68	68	0	0	0 (0)
Pseudomonas aeruginosa	64	49	13	2	15 (23.4)
Phaeobacter inhibens	62	62	0	0	0 (0)
Piscirickettsia salmonis	57	57	0	0	0 (0)
Zymomonas mobilis	51	51	0	0	0 (0)
Rhizobium leguminosarum	49	49	0	0	0 (0)
Serratia marcescens	49	5	26	18	44 (89.8)
**Firmicutes**					
Species	Total plasmids	Replicon carriage			
		Single-replicon	Multi-replicon	Untyped	Typed
Staphylococcus aureus	316	18	163	135	298 (94.3)
Bacillus thuringiensis	306	275	31	0	31 (10.1)
Lactobacillus plantarum	251	168	83	0	83 (33.1)
Enterococcus faecium	159	22	105	32	137 (86.2)
Lactococcus lactis	151	94	55	2	57 (37.7)
Bacillus cereus	104	98	6	0	6 (5.8)
Bacillus anthracis	70	36	34	0	34 (48.6)
Staphylococcus epidermidis	65	9	39	17	56 (86.2)
Clostridium botulinum	60	60	0	0	0 (0)
Enterococcus faecalis	60	10	38	12	50 (83.3)
**other**					
Species	Total plasmids	Replicon carriage			
		Single-replicon	Multi-replicon	Untyped	Typed
Borreliella burgdorferi	285	285	0	0	0 (0)
uncultured bacterium	267	210	50	7	57 (21.3)
Borreliella afzelii	54	54	0	0	0 (0)
Borreliella garinii	33	33	0	0	0 (0)
Rhodococcus hoagii	29	29	0	0	0 (0)
Bifidobacterium longum	27	27	0	0	0 (0)
Chlamydia trachomatis	27	27	0	0	0 (0)

Corynebacterium glutamicum	21	21	0	0	0 (0)
Mycobacterium chimaera	21	21	0	0	0 (0)
Salinibacter ruber	20	20	0	0	0 (0)

Taxonomic metadata was present for all plasmids, although not always informative (e.g. uncultured bacterium). For replicon carriage, single- and multi-replicon categories reflect the number of unique replicon types detected (e.g. IncFIB, IncFIC type is categorised multi-replicon whereas IncFIC, IncFIC is categorised single-replicon). Untyped means no replicon loci were detected on a plasmid. Typed means one or more replicon loci were detected on a plasmid. The Typed column includes number of plasmids replicon typed and % total plasmids replicon typed.

Supplementary Table 4. The top 10 most frequent isolation source sub-categories within each factor level of the isolation source explanatory variable (based on the dataset of 14143 plasmids).

**human**		**livestock**		**other**	
Sub-category	n	Sub-category	n	Sub-category	n
human	2645	aquaculture	192	uncategorised†	5541
sewage*	309	cow	156	-	4317
		chicken	155	agriculture††	623
		pig	135		
		turkey	26		
		sheep	16		
		poultry	13		
		goat	6		
		goose	4		
		duck	3		

*The sewage sub-category was manually curated with the aim of including only human-derived wastewater.
†Uncategorised means some taxonomic metadata was present (in any of the following fields used for curating isolation sources: *host*, *isolation_source*, *host_description*, *env_broad_scale*, *env_local_scale*, *env_medium*, description), but human/livestock/agriculture isolation source was not assigned; "-" indicates no metadata was present in any of the fields. BioSample metadata for the 5541 plasmids with 'uncategorised' isolation source is shown in Supplementary Data 1h.
††Non-livestock agriculture (this sub-category was ultimately included in the "other" factor level due to a relatively small sample size, and a primary interest in human vs livestock categories).

Supplementary Table 5. The top 10 most frequent countries within each factor-level of the geographic location explanatory variable (based on the dataset of 14143 plasmids)

**high-income not elsewhere classified**		**middle-income not elsewhere classified**		**European Union and the United Kingdom**		**China**	
Country	n	Country	n	Country	n	Country	n
South Korea	552	Mexico	150	Germany	328	China	1248
Japan	355	India	147	United Kingdom	157		
Canada	207	Russia	107	Spain	129		
Australia	115	Brazil	101	France	121		
Switzerland	99	Vietnam	51	Netherlands	85		

Chile	66	Thailand	49	Denmark	79		
Argentina	62	Malaysia	37	Sweden	66		
Norway	60	Colombia	30	Italy	53		
Hong Kong	42	Nigeria	24	Finland	32		
New Zealand	32	South Africa	24	Czechia, Greece, Ireland	29		
**United States**		**other**					
Country	n	Country	n				
United States	1491	-	7284				
		Taiwan*	93				
		Antarctica*	58				
		Réunion*	10				
		Nepal†	9				
		Raas Cabaad, Somalia†	8				
		Tanzania†	7				
		Syria†	6				
		Rwanda†	6				
		The Gambia†	5				

"-" indicates geographic location information was missing.
"high-income not elsewhere classified": World Bank high-income countries (not included in other categories).
"middle-income not elsewhere classified": World Bank lower-middle income countries (n = 362) and World Bank upper-middle income countries (n = 593) combined (not included in other categories).
"other": includes plasmids with missing location data, missing World Bank income categorisation (*), or rare income category (†) (specifically, World Bank low-income countries, n = 70).

A given plasmid can encode multiple replicon types; therefore, a plasmid can be assigned a haplotype representing the combination of encoded replicon types. In total, in the dataset of 14143 plasmids, there were 555 replicon type combinations (haplotypes). Plasmid replicon types can be grouped into higher-level replicon families (e.g. IncFIA, IncFIB etc. belong to the IncF replicon family). However, even at the replicon family level, there were 231 haplotypes (Supplementary Figure 3). Hence, for downstream statistical analysis, plasmid replicon types were re-coded to produce a 3-level replicon carriage variable (untyped, single-replicon, multi-replicon). Single- vs multi-replicon factor levels reflect the number of unique replicon types detected; for example, an "IncFIB, IncFIC" plasmid would be considered a multi-replicon plasmid whereas an "IncFIC, IncFIC" plasmid would be considered a single-replicon type plasmid.

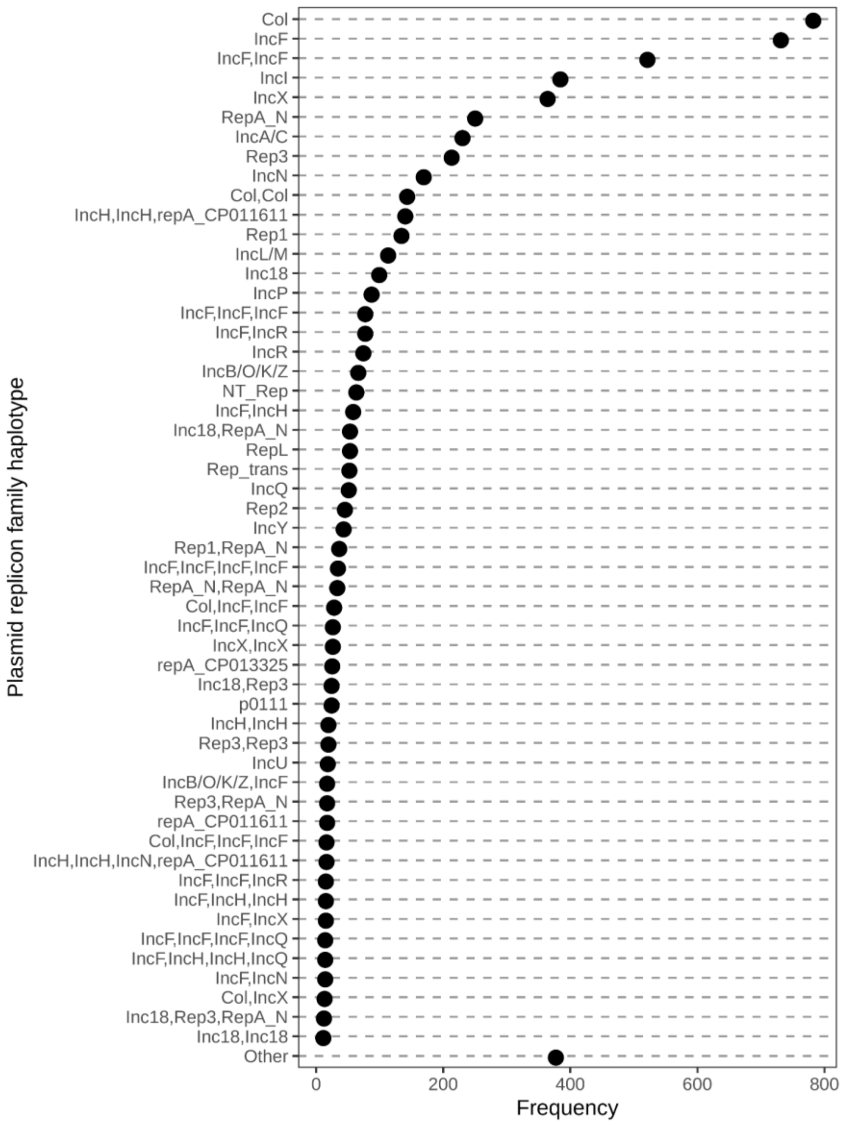

Supplementary Figure 3. Cleveland dotplot showing the frequency of plasmid replicon family haplotypes in the dataset of 14143 plasmids. In total, there were 231 replicon family haplotypes. Replicon haplotypes represented by fewer than 10 plasmids are categorised as "Other" in the dotplot. For 8230 plasmids, no replicon was detected.

Among plasmids with non-missing collection dates, there was weak correlation (Pearson's *r*=0.314) between accession create dates and collection dates (Supplementary Figure 4). The earliest create date was ~2000, whereas collection dates extended back to the early 20th century, so correlation is presumably very poor among plasmids collected pre-2000. However, even when restricting to plasmids collected since 2000, correlation remained weak (Pearson's *r*=318) (Supplementary Figure 5).

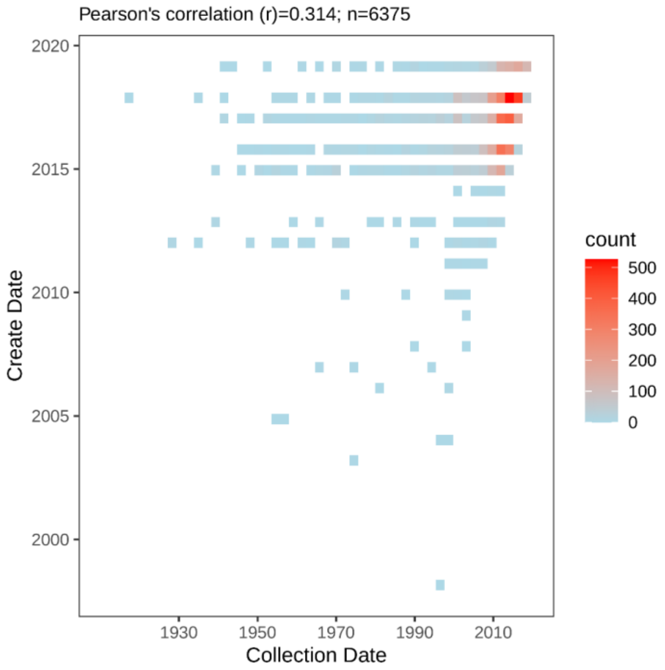

Supplementary Figure 4. Density scatter plot showing the relationship between collection date and create date, for all plasmids with non-missing collection dates. To handle overplotting, the datapoints were binned; the density of points within a bin is indicated using a blue-red colour gradient. The plot was generated using the stat_bin2d ggplot2 R function combined with custom R code available in a GitHub repository (PlasmidARGCarriage v1.0). (https://github.com/AlexOrlek/PlasmidARGCarriage/blob/v1.0/exploratory_analysis.R).

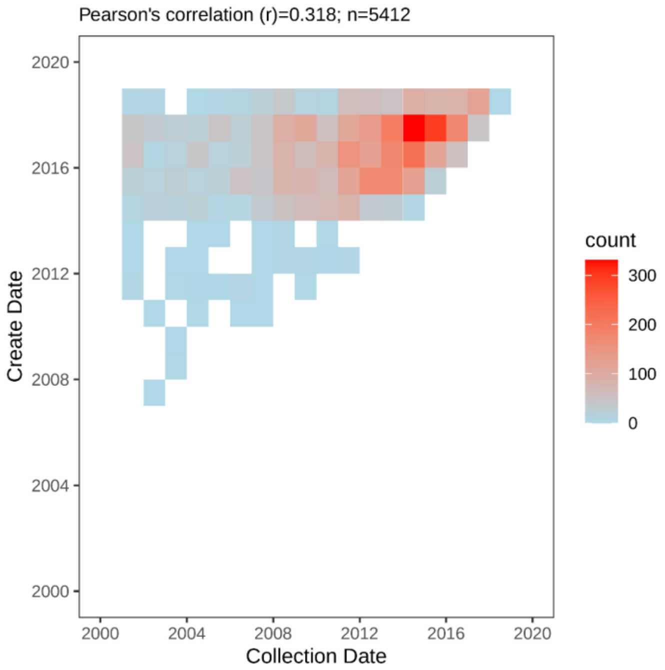

Supplementary Figure 5. Density scatter plot showing the relationship between collection date and create date, for plasmids with post-2000 collection dates. To handle overplotting, the datapoints were binned; the density of points within a bin is indicated using a blue-red colour

gradient. The plot was generated using the stat_bin2d ggplot2 R function combined with custom R code available in a GitHub repository (PlasmidARGCarriage v1.0). (https://github.com/AlexOrlek/PlasmidARGCarriage/blob/v1.0/exploratory_analysis.R).

1.5 Literature search strategy for determining dates of first recorded plasmid antibiotic resistance gene (ARG), for each ARG type

We determined dates of first recorded plasmid-mediated resistance for the 10 ARG types, by searching PubMed using the following term (substituting the name of each ARG type):

(plasmid OR transferable) AND *ARG type*

Early articles from the literature search were read, and relevant citations were followed-up until a plausible earliest article was retrieved, per ARG type. Separately, relevant review articles on the history of antibiotic resistance were retrieved by *ad hoc* searching, and relevant cited articles were read. From this process, we determined the date (year of publication) when the first article describing plasmid-mediated resistance was published, for each ARG type. In addition, where given, the collection date for the isolate described in the first article was determined.

1.6 GAM modelling methods

GAM models were constructed using the mgcv package [34], with the following structure:

```
gam(resistance ~ s(log10PlasmidSize, k = 5, pc = 0) + s(InsertionSequenceDensity, k = 5, pc = 0) + s(NumOtherResistanceTypes, k = 5, pc = 0) + s(CollectionDate, k = 5, pc = 0) + Integron + BiocideMetalResistance + Virulence + ConjugativeSystem + RepliconCarriage + HostTaxonomy + GeographicLocation + IsolationSource, family = ‘binomial’, data = FilteredPlasmids.tsv, method = ‘REML’, gamma = 1.5)
```

The gam.check() function was used to confirm model convergence, and test for non-random patterns in residuals (indicative of insufficient basis dimensionality). Smoothing parameters were selected using the maximum likelihood (ML) method initially; once model structure was confirmed, restricted maximum likelihood (REML) was used. ML/REML reduce overfitting compared with other available methods [35]. The gamma parameter was set to 1.5 to reduce overfitting [36]. For each GAM, the statistical significance of smooth and parametric (categorical) terms was tested using anova.gam() and summary.gam() functions, which implement Wald tests [34]. Smooth and parametric effect plots were visualised using mgcViz [37].

2 Results

2.1 Results of manual examination of identical plasmid accessions

To better understand redundancies, a subset of identical plasmid accessions which had been deduplicated automatically were selected for retrospective manual examination. According to NCBI documentation, when submitting to GenBank, BioSample and BioProject accessions should be used to delineate different isolates and sequencing projects, respectively [38]. However, we identified 8 clusters of identical plasmids comprising accessions with different BioSample identifiers, primary BioProject identifiers (the cognate GenBank BioProject accession identifier in the case of RefSeq accessions), as well as submitter contact name and affiliation (Supplementary Table 6). Manual examination suggested that 4/8 clusters could represent independently isolated plasmids (therefore, all plasmids belonging to these clusters were retained rather than deduplicated – that is, 4 plasmids that would otherwise have been excluded by automated deduplication of identical plasmids were instead retained). Other clusters comprised non-independent/redundant accessions (due to re-resubmission with semantically equivalent metadata, or sequencing of laboratory mutant derivatives). The same types of redundancy emerged when examining clusters of identical plasmids with different BioSample/primary BioProject identifiers but shared or missing submitter metadata (an arbitrary subsample of 8 of 121 such clusters was examined, see Supplementary Table 7).

Overall, plausible examples of independently isolated plasmids with 100% identical sequences are encountered, but very rarely. GenBank BioProject identifiers do not always delimit independent sequencing projects, but if multiple items of metadata are examined, duplicate accessions (i.e. redundant or otherwise non-independently isolated) commonly differ in one or more items (submitter metadata, BioSample/BioProject identifiers, strain names). These findings informed later methods for filtering similar plasmids (excluding replicate plasmids) to reduce bias from uneven sampling intensity, where multiple metadata items were used in conjunction with sequence similarity thresholds (Supplementary Figure 1).

Failure to sufficiently delimit independent sampling units and exclude duplicate and replicate plasmids accordingly may bias downstream inferences (e.g. of transmission links). Indeed, in a recent study, Douarre et al. identified 234 accession clusters comprising identical plasmids associated with the same species but different strain names, and concluded that these represented cases of intra-species plasmid dissemination [39]. Although the authors conducted some manual curation, duplicate clusters identified in our study through manual investigation (clusters 8, 10, 11, 13; see Supplementary Tables 6 and 7 below) appear to be spuriously included among the 234 intra-species plasmid dissemination clusters reported by Douarre et al. (see Supplementary Table 2 in Douarre et al.).

Supplementary Table 6. Manual examination of 8 clusters of identical accessions not sharing BioSample/primary BioProject identifiers or submitter name/affiliation metadata.

<table>
<tr><th>Clusters</th><th>BioSample accession id</th><th>Primary BioProject accession id</th><th>Submitter name</th><th>Affiliation name</th><th>Retained</th></tr>
<tr><td colspan="6">Cluster 1</td></tr>
<tr><td>MG710483.1</td><td>SAMN10679998</td><td>512490</td><td>Fabricio Campos</td><td>Federal University of Tocantins</td><td>✓</td></tr>
<tr><td>NZ_CP010009.1</td><td>SAMN03216682</td><td>238238</td><td>Hajnalka Daligault</td><td>Los Alamos National Laboratory</td><td>✓</td></tr>
<tr><td colspan="6">The plasmid accessions were isolated independently from different Bacillus thuringiensis serovars and from different isolation sources. According to BioSample metadata, accession MG710483.1 (strain Bti-UFT6.51; plasmid pBtiUFT6.51.2; B. thuringiensis serovar israelensis) was isolated from soil in Brazil in 2016; accession NZ_CP010009.1 (strain HD 1i; plasmid unnamed11; B. thuringiensis serovar kurstaki) was isolated from insect larvae in 2000. Consequently, both accessions were retained based on available information.
More details are provided by Campos et al. [40] who sequenced accession MG710483:
“In this work, we sequenced two plasmids found in a Brazilian Bacillus thuringiensis serovar israelensis strain which showed 100% nucleotide identities with Bacillus thuringiensis serovar kurstaki plasmids.”
(The other accessions mentioned as identical in Campos et al. (MG710485 and NZ_CP004874.1) actually show 99.9% identity and were therefore not detected as identical).
Accession MG710483.1 was Illumina sequenced and assembled using a reference-mapping approach (using accession NZ_CP010009.1 as the reference):
“DNA sequence assembly using the map to reference function in Geneious version 9.1.8 was used”.</td></tr>
<tr><td colspan="6">Cluster 2</td></tr>
<tr><td>NZ_CP013283.1</td><td>SAMN04288432</td><td>303961</td><td>Alexei Sorokin</td><td>MICALIS INRA</td><td>✗</td></tr>
<tr><td>NZ_CP009344.1</td><td>SAMN03010437</td><td>236049</td><td>Shannon Johnson</td><td>Los Alamos National Laboratory</td><td>✗</td></tr>
<tr><td colspan="6">NZ_CP013283.1 is a plasmid from a commercial strain of bioinsecticide (B. thuringiensis serovar israelensis strain AM65-52) [41]. My interest was in naturally occurring plasmids, so this accession was excluded. NZ_CP009344.1 was isolated from a sewage sample according to BioSample metadata. However, I decided to exclude this accession too in case of a transmission link with the plasmid from the commercial strain.</td></tr>
<tr><td colspan="6">Cluster 3</td></tr>
<tr><td>NZ_CP030795.1</td><td>SAMN09534371</td><td>230403</td><td>Peyton Smith</td><td>CDC</td><td>✓</td></tr>
<tr><td>NZ_CP018773.2</td><td>SAMN06159501</td><td>218110</td><td>Rebecca Lindsey</td><td>Centers for Disease Control and Prevention</td><td>✗</td></tr>
<tr><td colspan="6">Both accessions were submitted by the same institution (CDC), but indicated with different metadata text (CDC vs Centers for Disease Control and Prevention). Therefore, these accessions were deduplicated (based on a stringent criterion that non-duplicate accessions should have different submitter metadata as well as BioSample/BioProject identifiers).</td></tr>
<tr><td colspan="6">Cluster 4</td></tr>
<tr><td>NZ_CP016508.1</td><td>SAMN04334629</td><td>305824</td><td>Caroline Vincent</td><td>Laboratoire de sante publique du Quebec</td><td>✓</td></tr>
<tr><td>NZ_CP016523.1</td><td>SAMN05263513</td><td>298211</td><td>Roger Johnson</td><td>National Microbiology Laboratory at Guelph</td><td>✓</td></tr>
<tr><td colspan="6">NZ_CP016508.1 is from Salmonella enterica subsp. Enterica serovar Heidelberg, strain SH12-003, which was isolated from a Canadian (Quebec) hospital patient in 2012, according to BioSample metadata. NZ_CP016523.1 is from S. enterica subsp. Enterica serovar Heidelberg, strain SA02DT09004001, which was isolated from chicken meat from Canada (British Columbia) in 2009. S. Heidelberg is known to be a clonal serovar [42], so these accessions may represent a vertical transmission link between humans and poultry (Genevieve Labbé, pers. comm.). It was confirmed that the accessions were not redundant (i.e. did not represent re-submission of the same sequencing data) (Genevieve Labbé, pers. comm.).
NZ_CP016508.1 was Illumina sequenced and assembled using the MIRA assembler (https://sourceforge.net/p/mira-assembler/wiki/Home/), with a reference-mapping approach (the reference was NZ_CP016511.1) (Genevieve Labbé, pers. comm.).</td></tr>
<tr><td colspan="6">Cluster 5</td></tr>
</table>

NZ_CP025496.1	SAMN04966146	321117	Douglas Merrell	Uniformed Services University	✓
NZ_CP025490.1	SAMN03787328	287576	Ryan Johnson	Uniformed Services University of the Health Sciences	✓
NZ_CP025496.1 (*Staphylococcus aureus* subsp. *aureus*, strain 3020.C01) and NZ_CP025490.1 (*S. aureus* subsp. *aureus*, strain 2014.C01) are plasmids from clinical isolates. The isolates were collected from the same military battalion on 19th and 12th July 2011, respectively [43]. It was confirmed that these were non-redundant sequences from independent isolates (D. Scott Merrell, pers. comm.), so both accessions were retained.<br>Both accessions were Illumina sequenced, and it appears from LaBreck et al. [43] that contigs were assigned as plasmid/chromosomal using a reference-guided approach: “contig sequences were compared to each other, to published reference genomes, and to PCR, Sanger sequencing, and agarose gel electrophoresis results of restriction enzyme digested and non-digested DNA in order to correctly assign contigs as chromosomal or plasmid as well as to look for assembly artifacts.”					
**Cluster 6**					
NZ_CP009457.1	SAMN03078687	260989	Woori Kwak	Seoul National Univerisity	✓
NZ_CP011119.1	SAMN03434891	279015	Gnanasekaran Gopalsamy	Seoul National University	✗
Both accessions were submitted by the same institution, but indicated with different metadata text due to a typo). Therefore, these accessions were deduplicated.					
**Cluster 7**					
NZ_CP016567.1	SAMN05263514	298211	Roger Johnson	National Microbiology Laboratory at Guelph	✓
NZ_CP016509.1	SAMN04334629	305824	Caroline Vincent	Laboratoire de sante publique du Quebec	✓
NZ_CP016567.1 is from *S.* Heidelberg, strain AMR588-04-00318, which was isolated from chicken faeces from Canada (Ontario) in 2013, according to BioSample metadata. NZ_CP016509.1 is from *S.* Heidelberg, strain SH12-003, which was isolated from a hospital patient in Canada (Quebec) in 2012. It was confirmed that the accessions were not redundant (Genevieve Labbé, pers. comm.). As mentioned for cluster 4, this may represent a vertical rather than horizontal transmission link.<br>NZ_CP016509.1 was Illumina sequenced and assembled using the MIRA assembler (https://sourceforge.net/p/mira-assembler/wiki/Home/), with a reference-mapping approach (the reference was NZ_CP016583.1) (Genevieve Labbé, pers. comm.).					
**Cluster 8**					
NZ_CP013346.1	SAMN04288116	303954	Fusako Kawai	Kyoto Institute of Technology	✓
NZ_CP009431.1	SAMN03031197	260764	Yoshiyuki Ohtsubo	Tohoku university	✗
These accessions (NZ_CP013346.1, strain 203N, culture collection NBRC 111659; NZ_CP009431.1, strain 203, culture collection NBRC 15033) are not from independent strains; see Ohtsubo et al. [44,45]: “The complete genome of NBRC 15033 was determined, but the genes for PEG utilization were missing, and repeated cultivation was assumed to be the reason for the loss. From a laboratory stock, we recovered a strain, designated 203N, harboring the *pegA* gene and capable of growing on PEG” [44].					

Except for cluster 2 (see text), at least one accession per cluster was retained, while the second accession was either retained (✓) or excluded as a duplicate (✗), based on manual investigation. Accessions deemed non-duplicate fulfilled the following criteria: they did not share submitter metadata or BioSample/BioProject identifiers, and were confirmed as non-redundant/independently isolated following manual examination and submitter correspondence where necessary.

Acknowledgements: Sincere thanks to the following researchers for their correspondences regarding identical plasmid sequences: Genevieve Labbé and Roger Johnson (Cluster 4 and 7); Douglas Scott Merrell (Cluster 5).

Supplementary Table 7. Manual examination of a subset (n=8) of clusters of identical accessions not sharing BioSample/primary BioProject identifiers.

Cluster	BioSample accession id	Primary BioProject accession id	Submitter name	Affiliation name	Plausibly non-duplicate
**Cluster 9**					
MH785255.1	SAMN09846914	486725	Michal Bukowski	Jagiellonian University	✓
MH785230.1	SAMN09846907	486718	Michal Bukowski	Jagiellonian University	
Accessions MH785255.1 and MH785230.1 are from different strains (*Staphylococcus aureus* strains tu1 and ch8, respectively) [46].					
**Cluster 10**					
NZ_CP018678.1	SAMN05362953	328023	-	JCVI	✗
NZ_CP007713.1	SAMN02709859	242902	Hao Xu	California State University, Los Angeles	
The associated publication [47] states that "the LAC-4 genome consists of a circular chromosome of 3,954,354 base pairs and two circular plasmids, one with 8,006 base pairs while the other with 6,076 base pairs." Accessions NZ_CP018678.1 and NZ_CP007713.1 are both 8,006 bp and the recorded strain is "LAC4" and "LAC-4" respectively. Therefore, it appears that the accessions represent redundant re-submission of the same plasmid sequence with slightly different strain name.					
**Cluster 11**					
NZ_CP011933.1	SAMN03780437	287300	Kazuhito SATOU	Okinawa Institute of Advanced Sciences	✗
NZ_CP011936.1	SAMN03780438	287301	Kazuhito SATOU	Okinawa Institute of Advanced Sciences	
NZ_CP011933.1 and NZ_CP011936.1 (*Leptospira interrogans* serovar Manilae, strains UP-MMC-NIID LP and UP-MMC-NIID HP) are laboratory derivatives (Low and high passage [LP/HP]) of the same ancestral strain, as indicated in Satou et al. [48]: "*L. interrogans* serovar Manilae strain UP-MMC-NIID examined in this study had originally been isolated from the blood of a patient with severe leptospirosis. The virulent and avirulent variants were derived by serial subculture after 1 (low) and 67 (high) passages, respectively."					
**Cluster 12**					
NZ_CP010765.1	SAMN03294493	273605	Boyke Bunk	Leibniz Institute DSMZ	✓
NZ_CP010607.1	SAMN03294494	273606	Boyke Bunk	Leibniz Institute DSMZ	
NZ_CP010765.1 and NZ_CP010607.1 appear to be from distinct strains (*Phaeobacter inhibens* strains P80 and P83, respectively), isolated from the same location in Spain [49].					
**Cluster 13**					
NC_017720.1	SAMEA3138382	50407	-	EBI	✗
NC_016858.1	SAMN02602988	56087	-	NCBI	
These accessions represent a parental strain and its derivative, as indicated in Kröger et al. [50]: "Bacterial strain S. enterica serovar Typhimurium SL1344 [accession NC_017720.1] and its parental strain ST4/74 [accession NC_016858.1] were used throughout the study".					
**Cluster 14**					
NC_017151.1	SAMD00060949	31141	-	DDBJ	✗
NC_017135.1	SAMD00060948	31139	-	DDBJ	
NC_017147.1	SAMD00060947	31137	-	DDBJ	
NC_017127.1	SAMD00060946	31135	-	DDBJ	
NC_017110.1	SAMD00060945	31133	-	DDBJ	
NC_017119.1	SAMD00060944	31131	-	DDBJ	
NC_017114.1	SAMD00061107	32203	-	DDBJ	
NC_013212.1	SAMD00060943	31129	-	DDBJ	
These accessions are mutant derivatives from an experimental genome evolution study [51].					
**Cluster 15**					

NZ_CP010759.1	SAMN03294493	273605	Boyke Bunk	Leibniz Institute DSMZ	✓
NZ_CP010602.1	SAMN03294494	273606	Boyke Bunk	Leibniz Institute DSMZ	
NZ_CP010759.1 and NZ_CP010602.1 appear to be from distinct *Phaeobacter inhibens* strains (P80 and P83, respectively).					
**Cluster 16**					
NC_022605.1	SAMN02370325	222409	Feng-Jui Chen	National Health Research Institutes	✓
NC_017332.1	SAMEA2272282	36647	-	EBI	
Accessions NC_022605.1 and NC_017332.1 are from distinct strains of *Staphylococcus aureus* (strains Z172 and TW20, respectively) isolated from Taiwan and England, respectively [52,53].					

Following manual investigation, cluster accessions were assigned as plausibly non-duplicate (✓) or duplicate (×). In contrast to clusters 1–8, submitters were not contacted to confirm whether plausibly non-duplicate cluster accessions were indeed non-duplicates, and therefore none of these accessions were retained following automated deduplication.

2.2 Results of data retrieval, curation, and cleaning

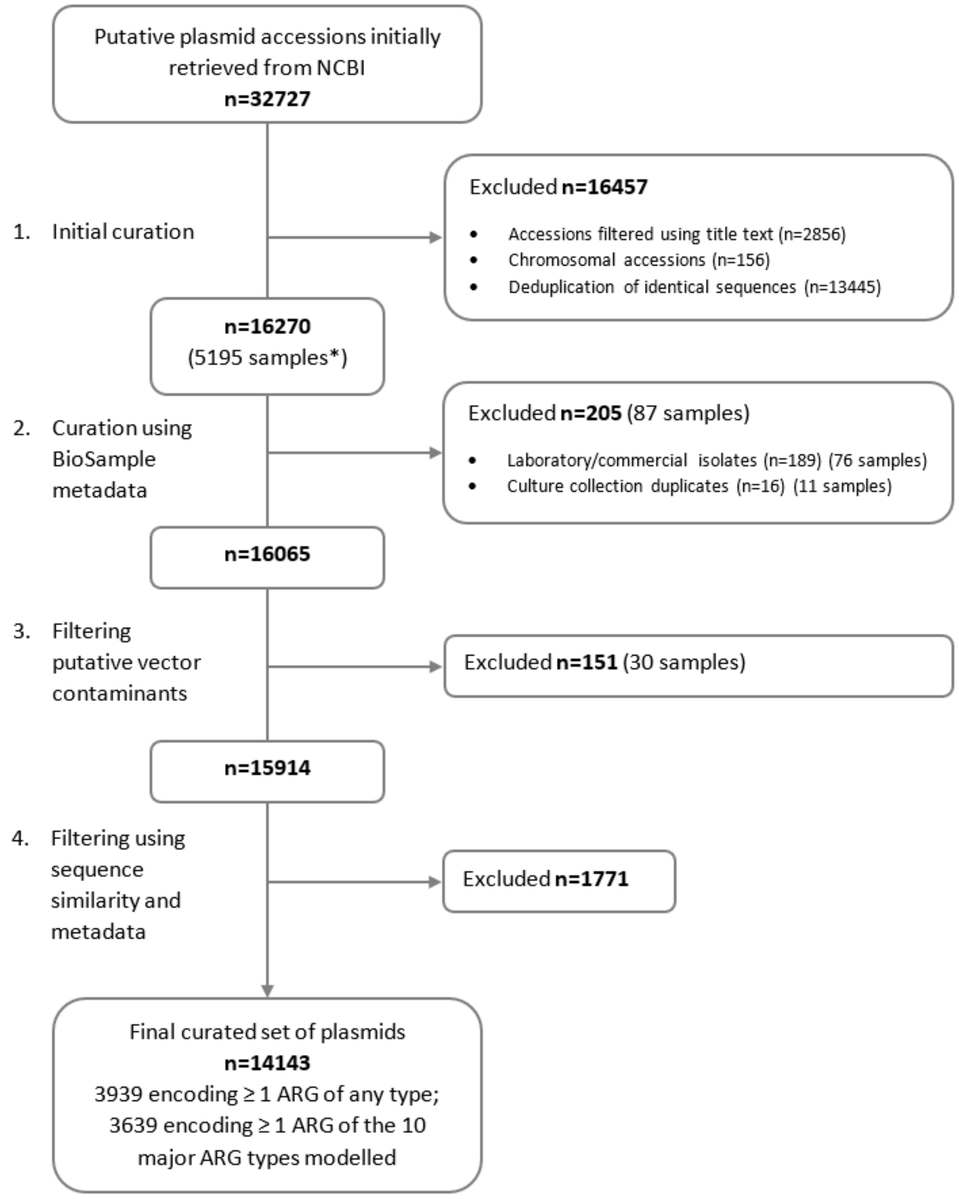

Supplementary Figure 6. Plasmid dataset curation flowchart. Flowchart indicates numbers of plasmid accessions during the curation process from initial retrieval (n=32727) to the final filtered dataset of curated plasmids (n=14143). The number of plasmids excluded at curation steps is shown on the right. Curation steps are described in text on the left. From the dataset of 14143 plasmids, a subset of 3639 encoding ≥ 1 major antibiotic resistance gene (ARG) type (of the 10 major ARG types modelled), were visualised using Microreact, to show the global distribution of antibiotic resistance plasmids in our analysis.

*Of 16270 plasmids retained after initial curation, 11848 (71%) were linked to a BioSample accession (5195 BioSample accessions).

Supplementary Data 1. Tabular data (.xlsx file, sheets A–I). The data can be accessed here: https://github.com/AlexOrlek/PlasmidAMRCarriage_paper/blob/main/data/Data_S1.xlsx

a Geocoding results for BioSample accessions for which there was a discrepancy between the geocoded latitude/longitude (derived from the *geo_loc_name* BioSample attribute) and the BioSample latitude/longitude (*lat_lon* attribute). Discrepancies were identified if inter-coordinate geodesic distance exceeded 50 km, and the *lat_lon* coordinate fell outside the geocoded Google map viewport. A discrepancy category is assigned to indicate whether discrepancies are likely to reflect *lat_lon* coordinate error, or geocoded coordinate error, or the reason is unclear (respectively labelled: biosample latlon is invalid, biosample latlon is valid, biosample latlon is discrepant).

b Culture collection samples with linked Bac*Dive* metadata are shown; the Bac*Dive*-guided curation of host, isolation source, and geographic location metadata is indicated. BioSample metadata prior to Bac*Dive*-guided curation has a pink header while post-Bac*Dive* curated metadata has a green header. Bac*Dive* metadata used for curation is given to the right. In the green headed section, yellow fill indicates addition of metadata where metadata was previously missing; orange fill indicates correction to previous metadata; blue fill indicates addition/clarification to previous metadata. The rightmost columns document the curation process and where possible explain discrepancies (in the Notes column). In the Notes column, yellow fill indicates cases where species authority date is given instead of genuine collection date.

c Samples with early (pre-1950) collection dates are shown. Two collection date fields are given (blue text): the collection_date field contains the original collection date metadata retrieved from NCBI BioSample. The collection_date_curated field contains the collection metadata after curation (after removing invalid metadata such as 'unknown' and conducting Bac*Dive*-guided curation). The rightmost columns document the manual curation of the pre-1950 dates. During manual curation, incorrect dates were removed from the collection_date_curated field, as indicated in the Notes column. In the Notes column, yellow fill indicates cases where species authority date is given instead of genuine collection date.

d–f Sheets D and E show BioSample accessions which were included and excluded (respectively) following metadata curation and vector contaminant filtering steps (117 samples were excluded comprising 356 plasmids; see Supplementary Figure 6). Associated raw and '_curated' metadata columns are provided. Sheet F lists the 356 plasmids which were excluded following the metadata curation and vector contaminant filtering steps, with reason for exclusion indicated.

g The set of 15914 plasmids (prior to final filtering step; see Supplementary Figure 6). The set of 14143 plasmids which were included in the final filtered dataset used for downstream statistical analyses are indicated (column labelled 'InFinalDataset').

h BioSample metadata for the 5541 plasmids with 'uncategorised' isolation source.

i Results of ARG annotation using the ResFinder database. Detected resistance genes for each plasmid are listed (set of 14143 plasmids used for statistical analysis). The ARGProbe column refers to the gene probe name as recorded in ResFinder. ARGName is the gene name extracted from the probe name. ARGClass is the gene class as per ResFinder database sub-division.

ARGType is a modification of ARGClass with beta-lactam sub-types (including carbapenem and ESBL) appended. ARGlabel is a label constructed from gene name and ARGType.

j The set of 14143 plasmids used for statistical analysis; transformed explanatory variables (following winsorising and re-factoring) are provided.

2.3 Results of statistical analysis (exploratory, unadjusted, and adjusted analysis)

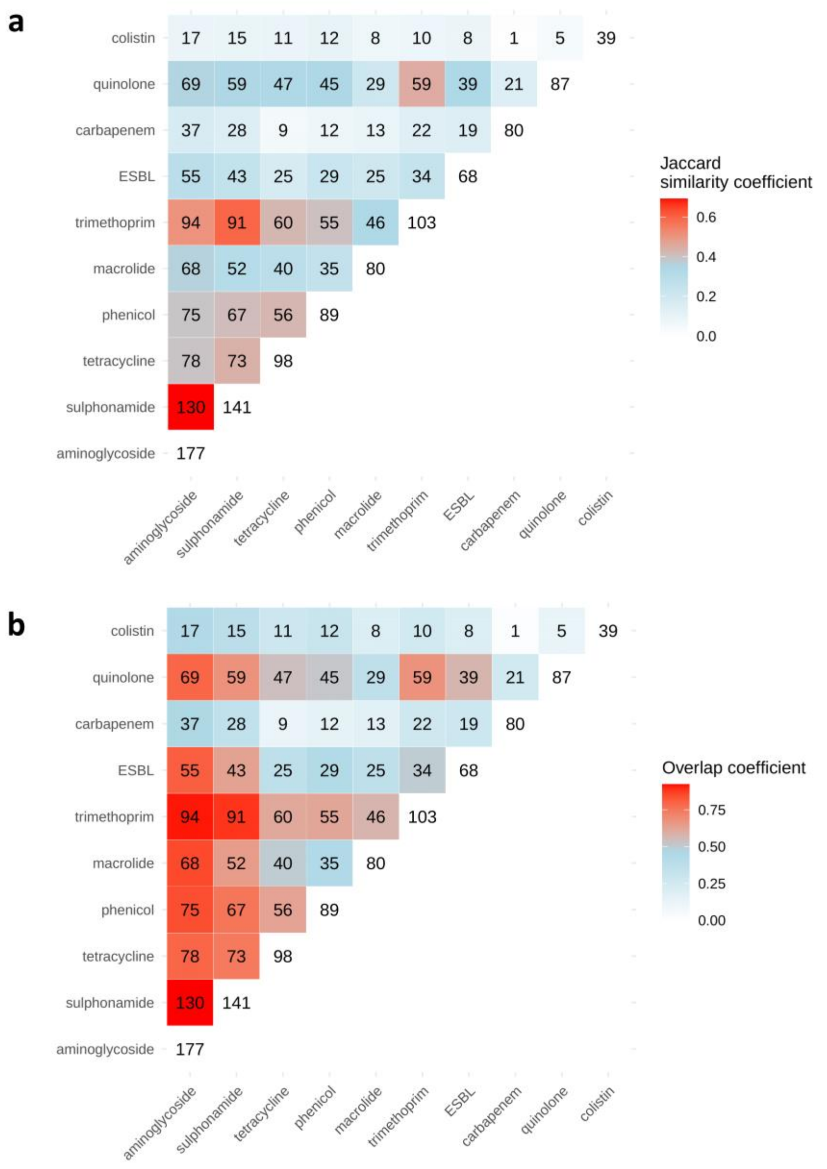

Supplementary Figure 7. Co-occurrence of antibiotic resistance gene (ARG) types is determined from their presence/absence in the dataset of 1007 plasmids with collection dates 2016–2019, and visualised using heatmaps. ARG types are ordered by the inferred timeline of known plasmid-mediated resistance acquisition (see Table 2) from earliest (aminoglycoside,

sulphonamide) to most recent (colistin). Counts along the diagonal indicate total plasmids carrying a given ARG type. Counts in the upper-left triangle indicate pairwise ARG type intersections i.e. the number of plasmids where a given pair of ARG types co-occur. Heatmaps are coloured by similarity metrics (a Jaccard index, b overlap coefficient) indicating the degree of co-occurrence between ARG types (red = more co-occurrence; light blue = less co-occurrence). Heatmaps were generated using custom R code available in a GitHub repository (PlasmidARGCarriage v1.0). (https://github.com/AlexOrlek/PlasmidARGCarriage/blob/v1.0/exploratory_analysis.R).

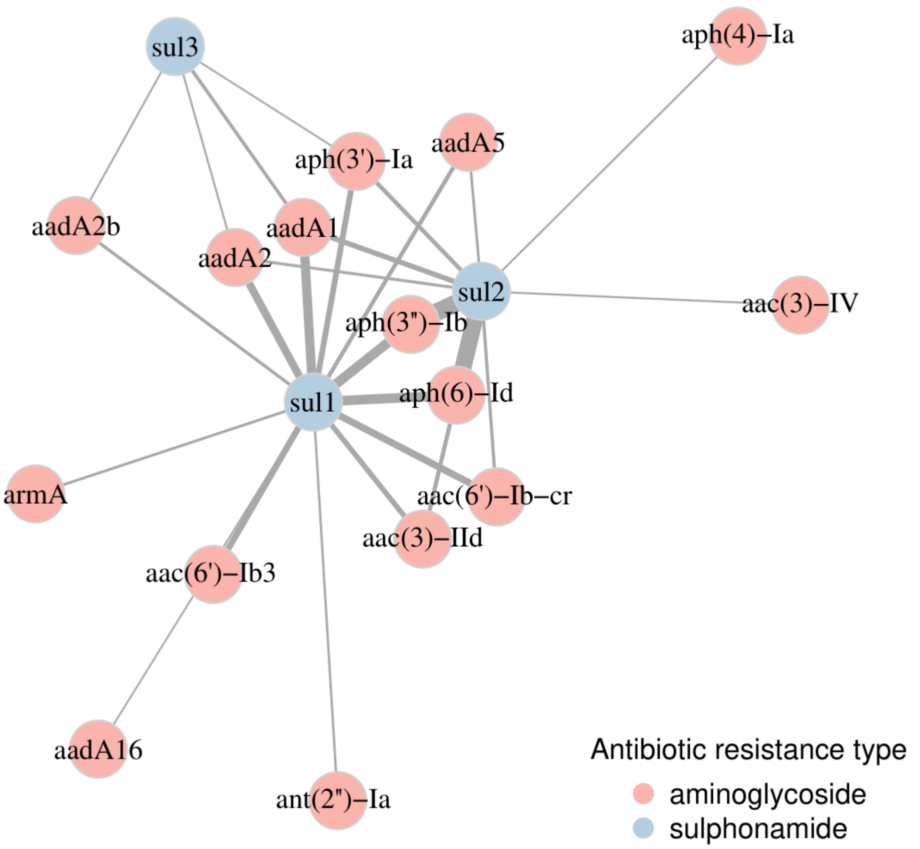

Supplementary Figure 8. Co-occurrence network of aminoglycoside and sulphonamide antibiotic resistance genes (ARGs) built using igraph, based on ARGs detected in the dataset of 14143 curated plasmids. Nodes are aminoglycoside and sulphonamide ARGs. Edges represents pairwise co-occurrence between aminoglycoside–sulphonamide gene pairs; edge thickness is scaled according to the number of plasmids where the co-occurring genes occur. The network was pruned to exclude edges represented by fewer than 50 plasmids. The most frequently co-occurring gene pairs were *aph(3'')-Ib–sul2* (n=491) and *aph(6)-Id–sul2* (n=470). Note that the gene *aac(6')-Ib-cr* confers both aminoglycoside and quinolone resistance.

Supplementary Data 2 Tabular data (.xlsx file, sheets A–C). The data can be accessed here: https://github.com/AlexOrlek/PlasmidAMRCarriage_paper/blob/main/data/Data_S2.xlsx

a Association statistics between explanatory variables. A colour scale indicates the absolute value of association statistics which range between 0 to 1 or -1 (Spearman's correlation coefficient) or between 0 to 1 (Cramer's statistic and Kruskall-Wallis eta statistic).

b Cross tabulations of categorical explanatory variables and ARG type presence/absence are provided, across all ARG type outcomes. Unadjusted odds ratios, 95% confidence intervals, and p-values are also provided. Odds ratios and 95% confidence intervals are also presented on the log-scale.

c Adjusted odds ratios, 95% confidence intervals, and p-values for each parametric term in the full GAM model (as outlined in main text Methods). Odds ratios and 95% confidence intervals are also presented on the log-scale. The difference between unadjusted and adjusted coefficients is also given; grey shading indicates the magnitude of the difference (darker indicates a larger difference between unadjusted and adjusted coefficients); positive/negative differences (relative to unadjusted coefficients) are indicated using red/green font, respectively.

2.4 Model checking

All GAM models converged. Basis dimensionality checking indicated non-random patterns in the residuals for $\log_{10}$ plasmid size smooths across all models, and for insertion sequence density smooths in 5/10 models. This was not resolved by increasing the basis dimensionality. The terms were retained, but the smooths should be interpreted cautiously. Model R^2 values ranged from 0.26 (ESBL) to 0.77 (sulphonamide).

2.5 Categorical explanatory variable effect plots

All multivariable-adjusted plots shown in this section are from the full model.

a **Unadjusted** **b** **Adjusted**

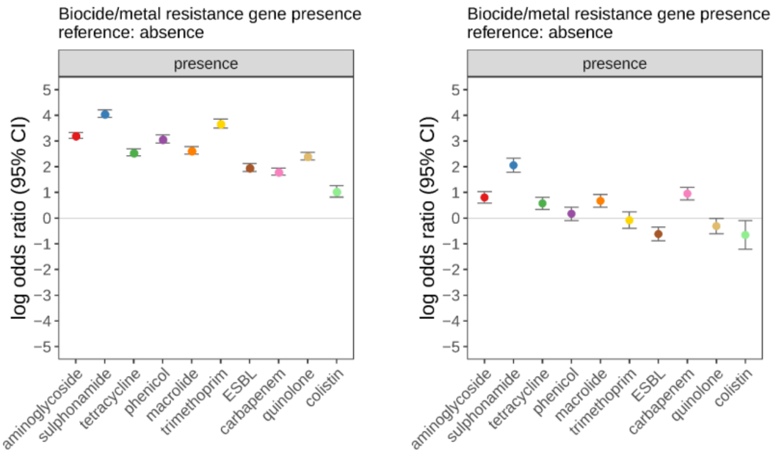

Supplementary Figure 9. The association between biocide/metal resistance gene presence (vs absence) and the log-odds of antibiotic resistance carriage (y-axis), across 10 ARG types. **a** log-odds ratios from the unadjusted analysis. **b** log-odds ratios from the adjusted analysis. Log-odds ratios indicate the effect of biocide/metal resistance gene presence, relative to reference (absence), and error bars show 95% confidence intervals.

a Unadjusted

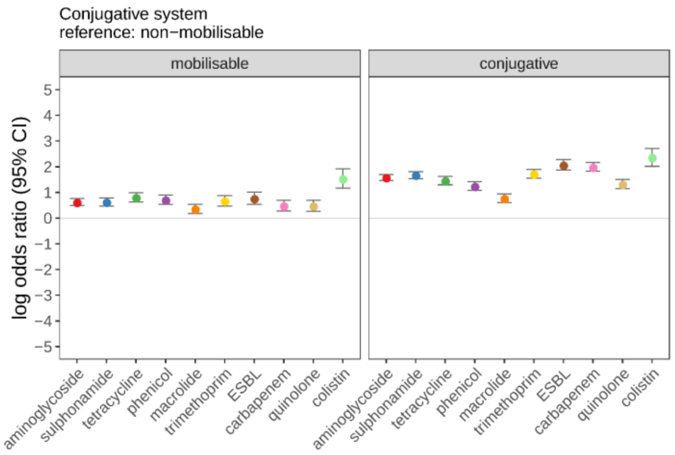

b Adjusted

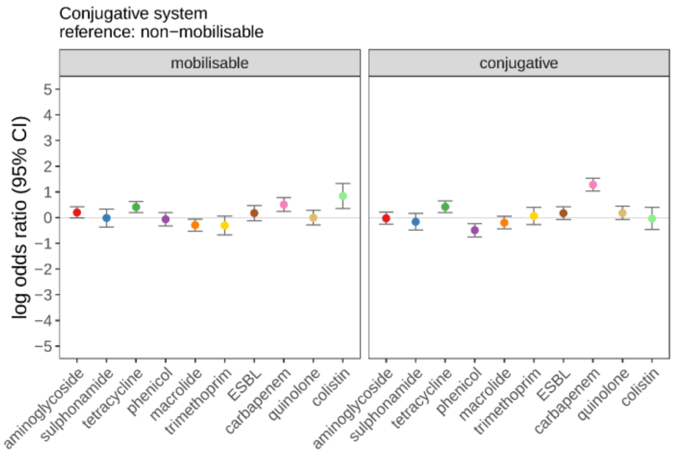

Supplementary Figure 10. The association between conjugative system (non-mobilisable [reference], mobilisable, conjugative) and the log-odds of antibiotic resistance carriage (y-axis), across 10 ARG types. **a** log-odds ratios from the unadjusted analysis. **b** log-odds ratios from the adjusted analysis. Log-odds ratios indicate the effect of a given factor level, relative to reference (non-mobilisable), and error bars show 95% confidence intervals.

a Unadjusted

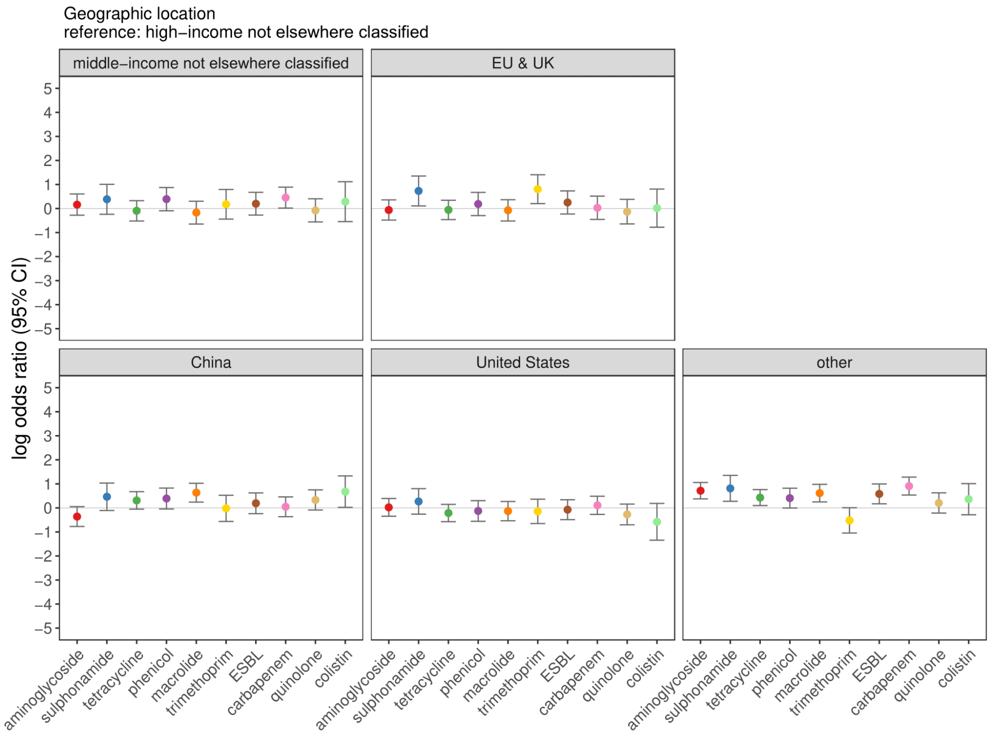

b Adjusted

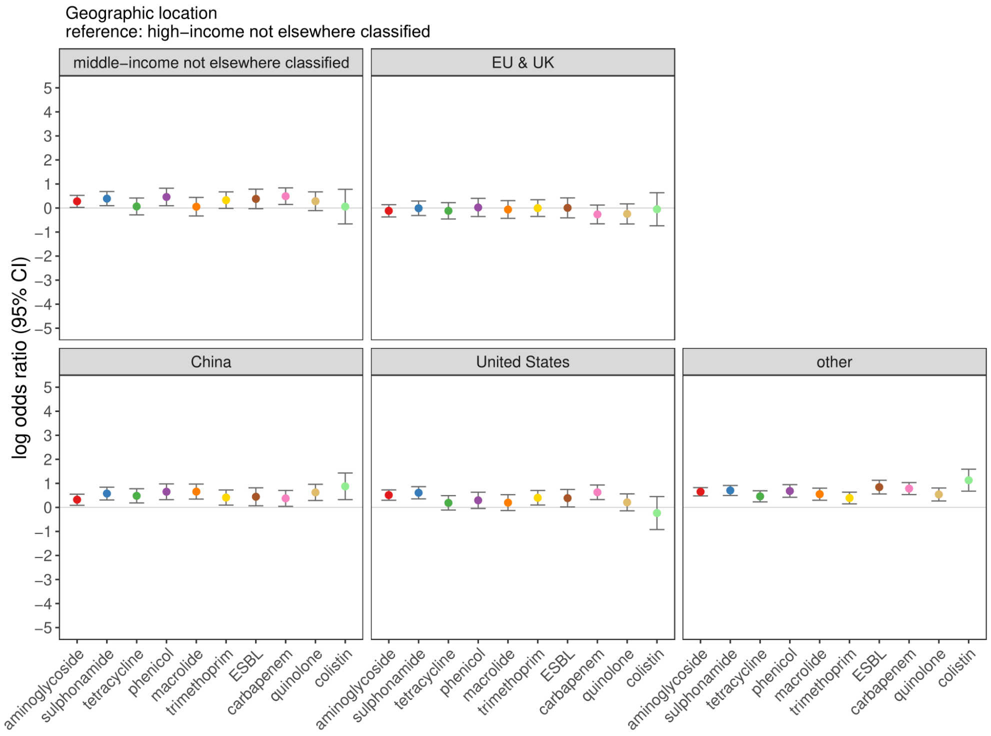

Supplementary Figure 11. The association between geographic location and the log-odds of antibiotic resistance carriage (y-axis), across 10 ARG types. **a** log-odds ratios from the unadjusted analysis. **b** log-odds ratios from the adjusted analysis. Log-odds ratios indicate the effect of a given factor level, relative to reference (high-income not elsewhere classified), and error bars show 95% confidence intervals.

a Unadjusted

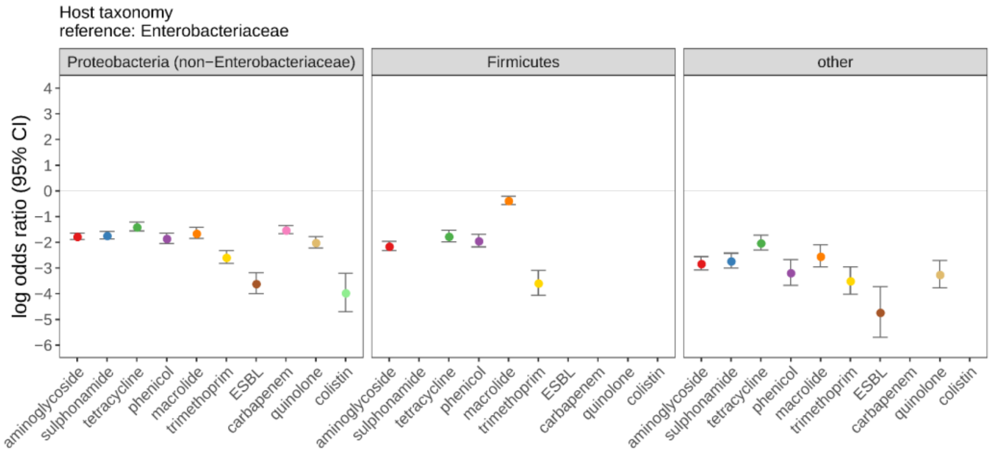

b Adjusted

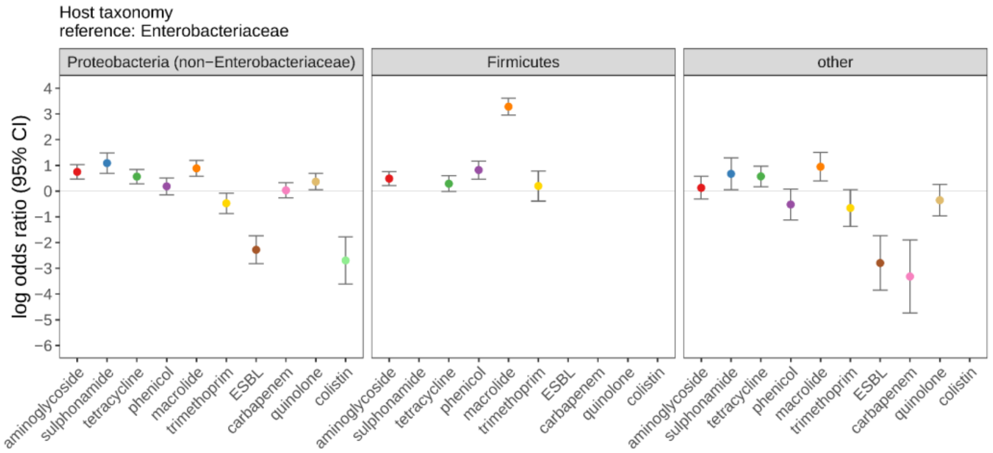

Supplementary Figure 12. The association between host taxonomy (Enterobacteriaceae [reference], Proteobacteria (non-Enterobacteriaceae), Firmicutes, other) and the log-odds of antibiotic resistance carriage (y-axis), across 10 ARG types. **a** log-odds ratios from the unadjusted analysis. **b** log-odds ratios from the adjusted analysis. Log-odds ratios indicate the effect of a given factor level, relative to reference (Enterobacteriaceae), and error bars show 95% confidence intervals.

a **Unadjusted** **b** **Adjusted**

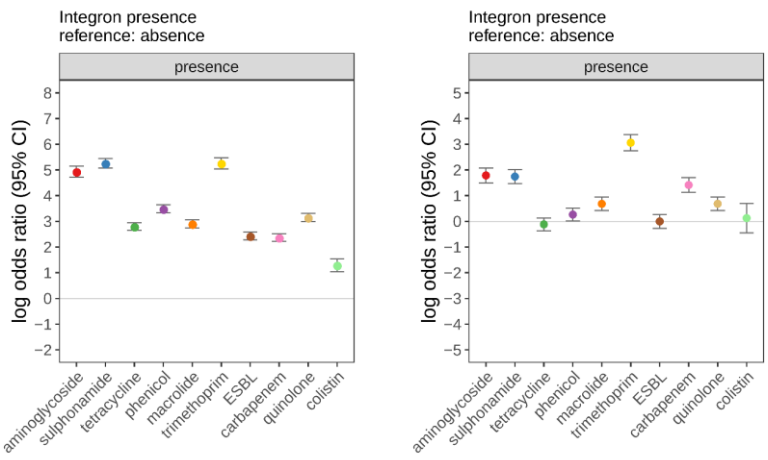

Supplementary Figure 13. The association between integron presence (vs absence) and the log-odds of antibiotic resistance carriage (y-axis), across 10 ARG types. **a** log-odds ratios from the unadjusted analysis. **b** log-odds ratios from the adjusted analysis. Log-odds ratios indicate the effect of integron presence, relative to reference (absence), and error bars show 95% confidence intervals.

a Unadjusted

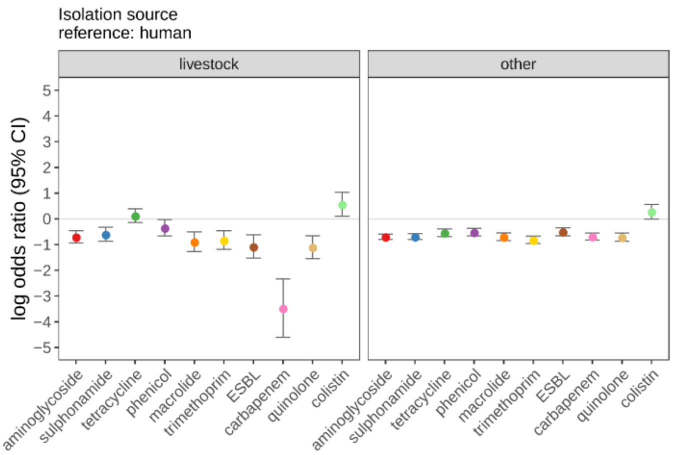

b Adjusted

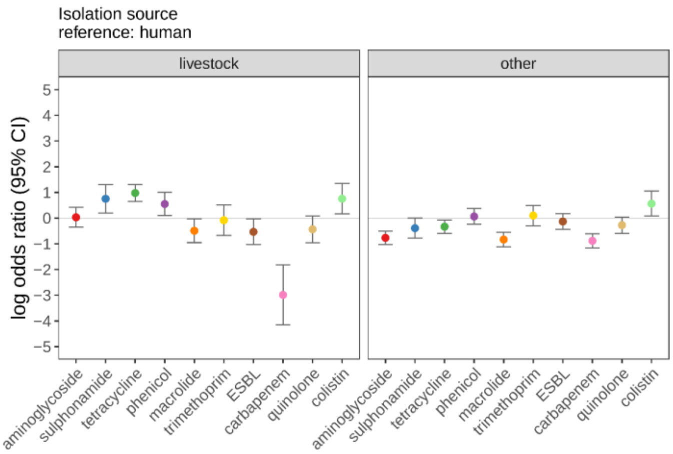

Supplementary Figure 14. The association between isolation source (human [reference], livestock, other) and the log-odds of antibiotic resistance carriage (y-axis), across 10 ARG types. **a** log-odds ratios from the unadjusted analysis. **b** log-odds ratios from the adjusted analysis. Log-odds ratios indicate the effect of a given factor level, relative to reference (human), and error bars show 95% confidence intervals.

a **Unadjusted**

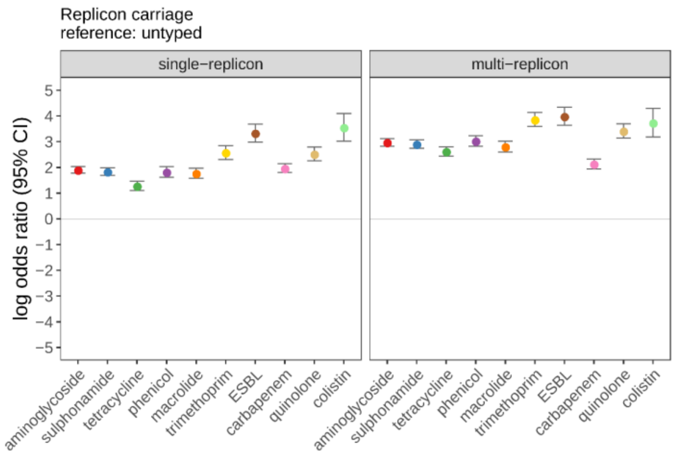

b **Adjusted**

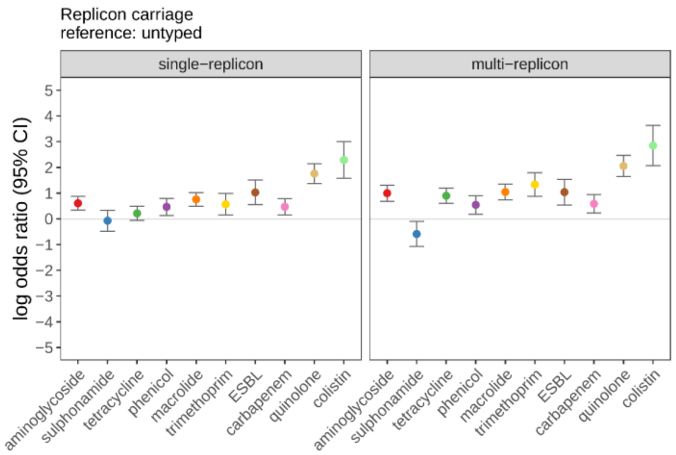

Supplementary Figure 15. The association between replicon carriage (untyped [reference], single-replicon, multi-replicon) and the log-odds of antibiotic resistance carriage (y-axis), across 10 ARG types. **a** log-odds ratios from the unadjusted analysis. **b** log-odds ratios from the adjusted analysis. Log-odds ratios indicate the effect of a given factor level, relative to reference (untyped), and error bars show 95% confidence intervals.

a **Unadjusted** **b** **Adjusted**

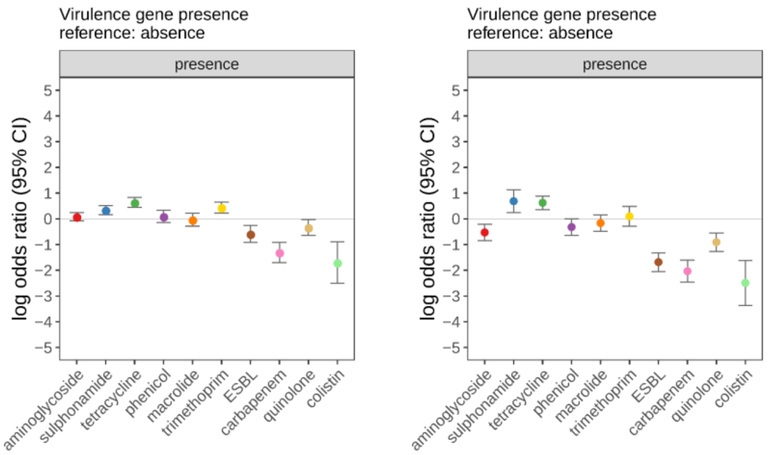

Supplementary Figure 16. The association between virulence gene presence (vs absence) and the log-odds of antibiotic resistance carriage (y-axis), across 10 ARG types. **a** log-odds ratios from the unadjusted analysis. **b** log-odds ratios from the adjusted analysis. Log-odds ratios indicate the effect of virulence gene presence, relative to reference (absence), and error bars show 95% confidence intervals.

2.6 Continuous explanatory variable effect plots

All plots shown in this section are from the full model.

a Unadjusted

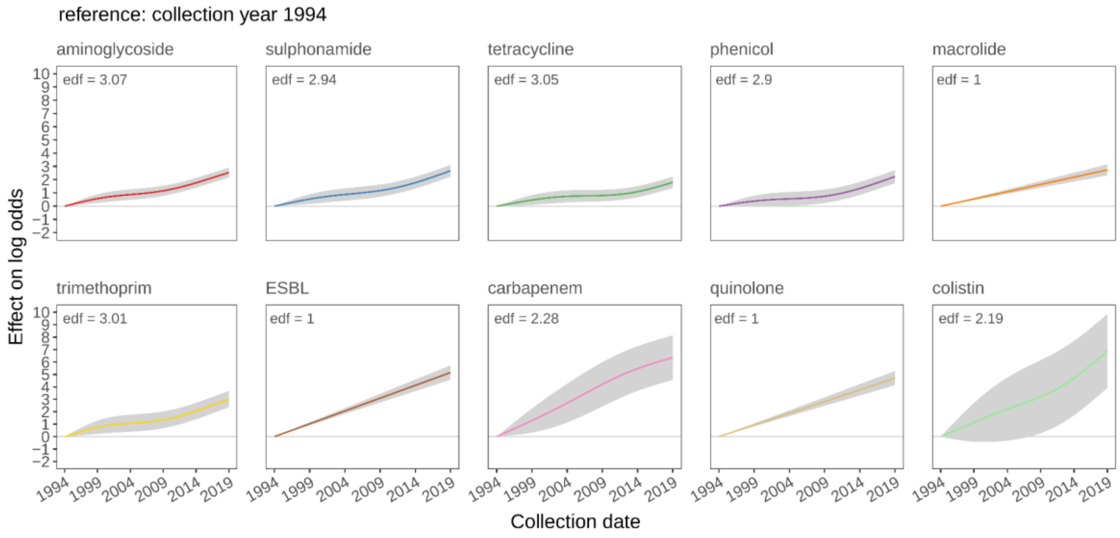

b Adjusted

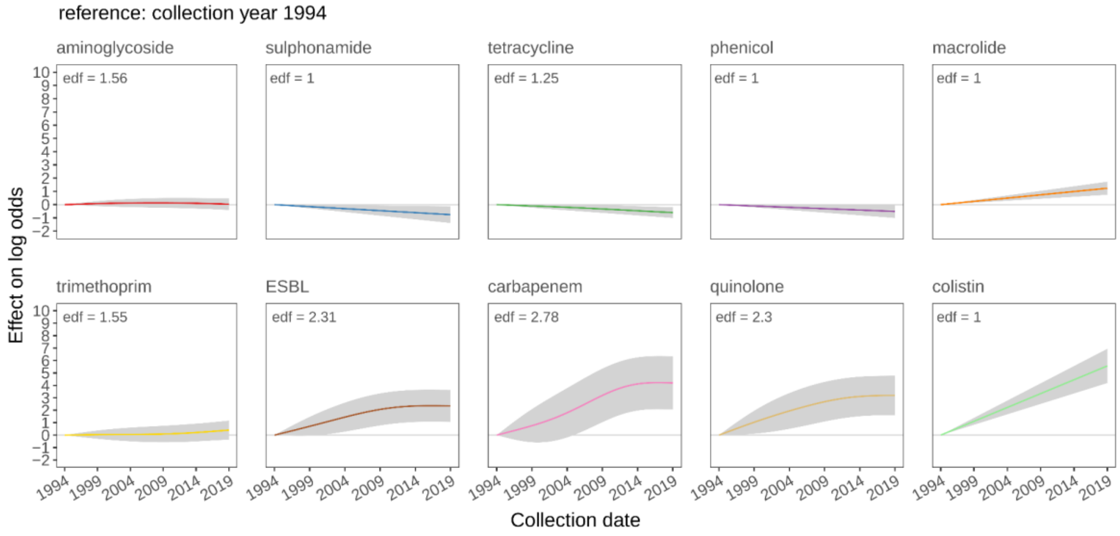

Supplementary Figure 17. The association between collection date and the log-odds of antibiotic resistance carriage (y-axis), across 10 ARG types. **a** effect on log-odds from the unadjusted analysis **b** effect on log-odds from the adjusted analysis. The grey shading around estimated smooth lines indicates 95% confidence limits.

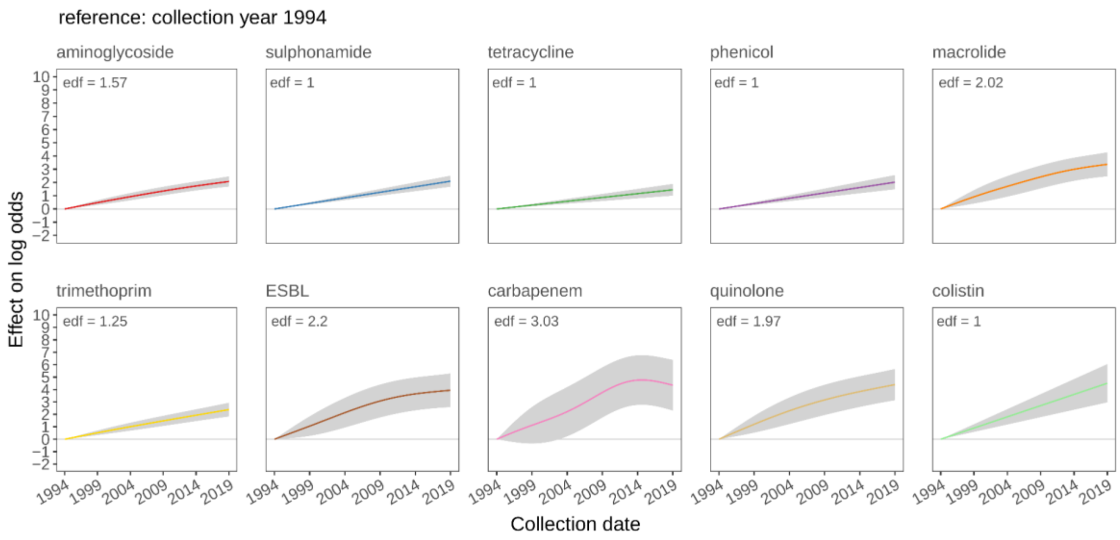

Supplementary Figure 18. Unadjusted analysis of the association between collection date and the log-odds of antibiotic resistance carriage (y-axis), across 10 ARG types, based on a subset of plasmids (n=6375) with non-imputed collection dates only. The grey shading around estimated smooth lines indicates 95% confidence limits.

a Unadjusted

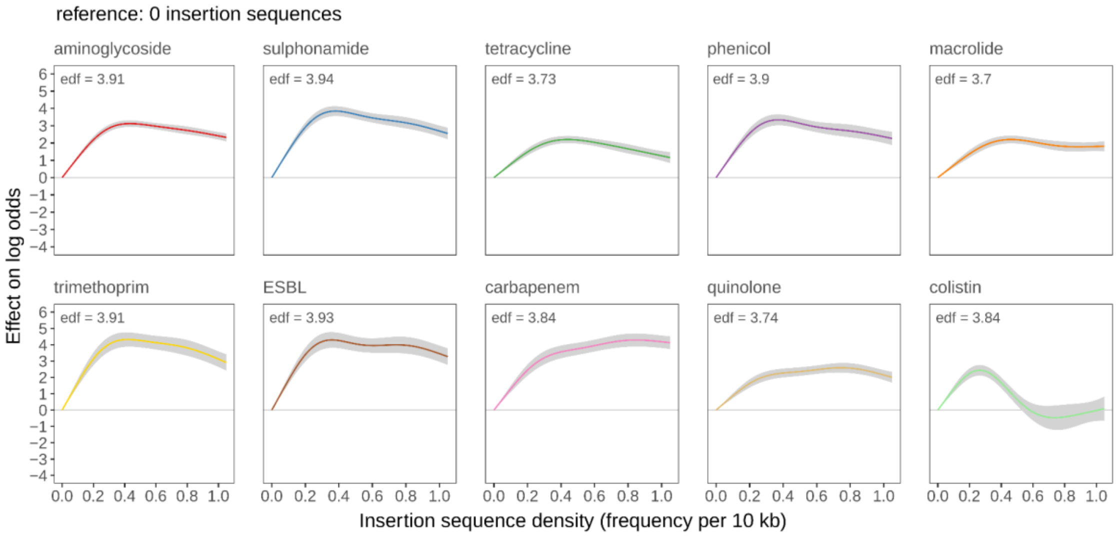

b Adjusted

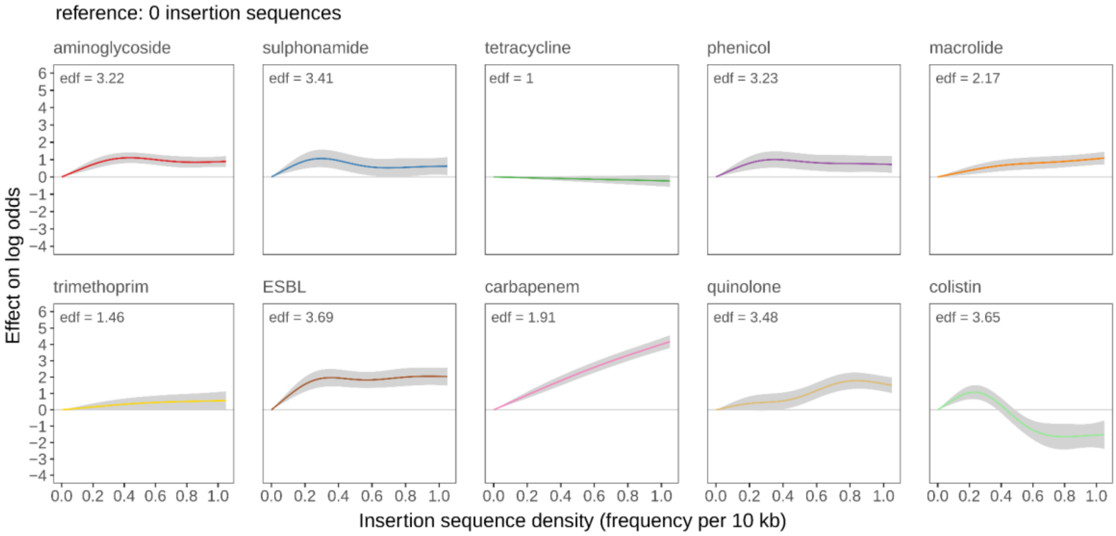

Supplementary Figure 19. The association between the insertion sequence density and the log-odds of antibiotic resistance carriage (y-axis), across 10 ARG types. **a** effect on log-odds from the unadjusted analysis **b** effect on log-odds from the adjusted analysis. The grey shading around estimated smooth lines indicates 95% confidence limits.

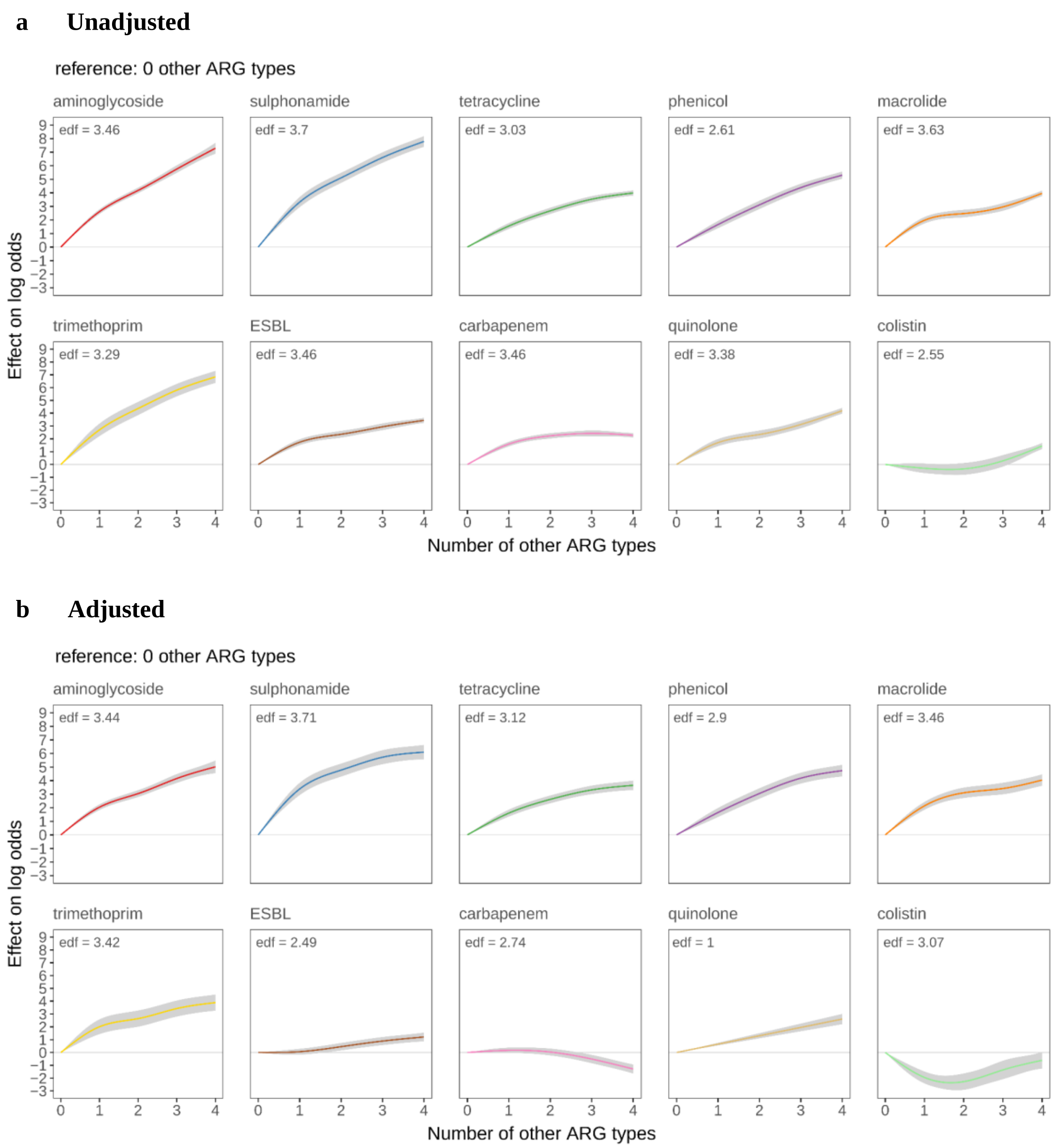

Supplementary Figure 20. The association between the number of other ARG types and the log-odds of antibiotic resistance carriage (y-axis), across 10 ARG types. **a** effect on log-odds from the unadjusted analysis **b** effect on log-odds from the adjusted analysis. The grey shading around estimated smooth lines indicates 95% confidence limits.

a Unadjusted

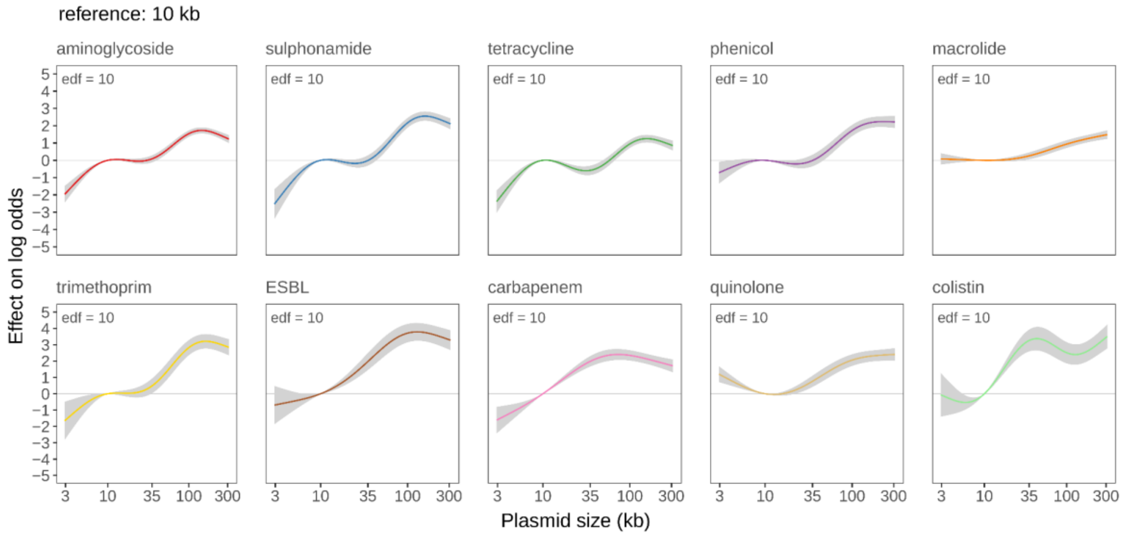

b Adjusted

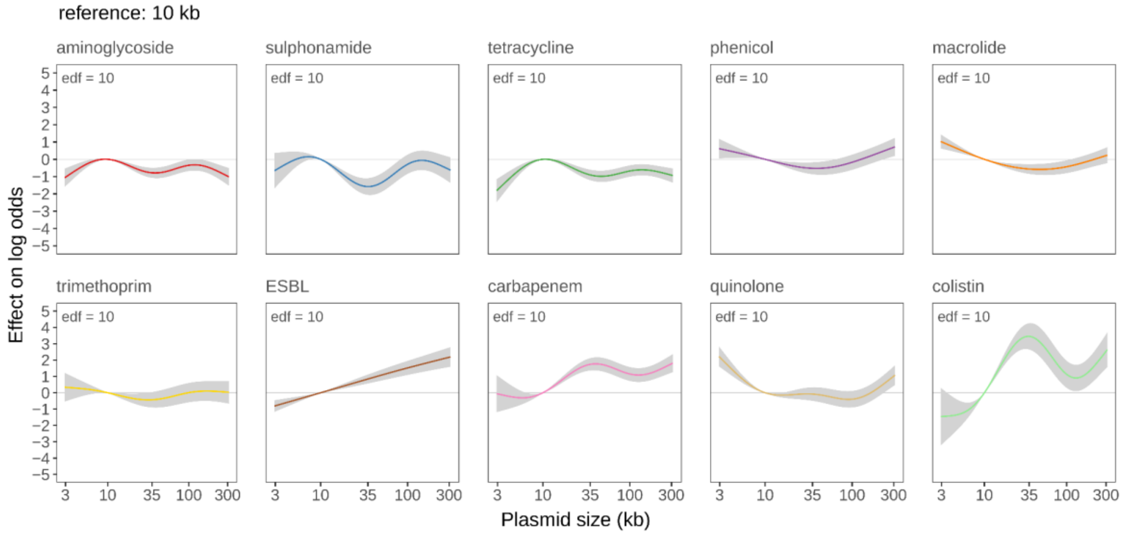

Supplementary Figure 21. The association between plasmid size (log10-transformed and centred on 10 kb) and the log-odds of antibiotic resistance carriage (y-axis), across 10 ARG types. **a** effect on log-odds from the unadjusted analysis **b** effect on log-odds from the adjusted analysis. The grey shading around estimated smooth lines indicates 95% confidence limits.

Supplementary Table 8. Frequency table of quinolone resistance genes encoded by small plasmids (<10kb).

Quinolone gene name	n
qnrD1	32
qnrS2	19
qnrB19	16
aac(6')-Ib-cr	2
qepA3	2
qnrD2	2
qnrVC5	2
qnrS6	1

Table shows quinolone genes encoded by small plasmids (<10kb) and the number of plasmids encoding each gene; a gene was counted no more than once per plasmid. The replicon type combinations ("haplotypes") of the plasmids encoding the top 3 genes are as follows: *qnrD*: Col3M (30); Col3M,Col3M (2). *qnrS2*: IncQ2 (15); IncQ1 (2); untyped (2). *qnrB19*: Col4401 (15); untyped (1).
Note, *aac(6')-Ib-cr* confers both quinolone and aminoglycoside resistance.

2.7 Investigation of confounding

Differences between unadjusted and adjusted odds ratios were noted in the main text (see also Sections 2.5 and 2.6 above). These differences can be explained by confounding interrelationships between explanatory variables, as described below. To investigate confounding, alternative adjusted models were fitted, with various terms removed relative to the main model. The terms selected for removal were guided by the exploratory association statistics (see Supplementary Data 2a – for a given explanatory variable where differences between adjusted and unadjusted effects were observed, more highly associated explanatory variables were preferentially removed).

2.7.1 Host taxonomy

For host taxonomy, the unadjusted analysis showed negative associations with resistance carriage for Proteobacteria (non-Enterobacteriaceae), Firmicutes, and other bacteria, relative to Enterobacteriaceae (the reference factor level), across all ARG type outcomes. However, in the adjusted analysis, negative associations were attenuated or reversed (Supplementary Figure 12). For example, multivariable-adjusted analysis suggested that Firmicutes plasmids were more likely to carry macrolide ARGs compared with Enterobacterial plasmids, whereas unadjusted analysis indicated the reverse.

The association statistics indicated that host taxonomy was most strongly associated with replicon carriage and the number of other ARG types (Supplementary Data 2a). Moreover, when these two factors were removed from the multivariable model, the host taxonomy adjusted log-odds ratios shifted to around halfway between adjusted and unadjusted log-odds ratios (Supplementary Figure 22). The GAM modelling results showed that replicon carriage and number of other ARG types were both positively associated with resistance carriage (see Supplementary Figures 15, 20). Hence, the difference between unadjusted/adjusted effects may

at least partly result from confounding with the effects of replicon carriage and number of other ARG types.

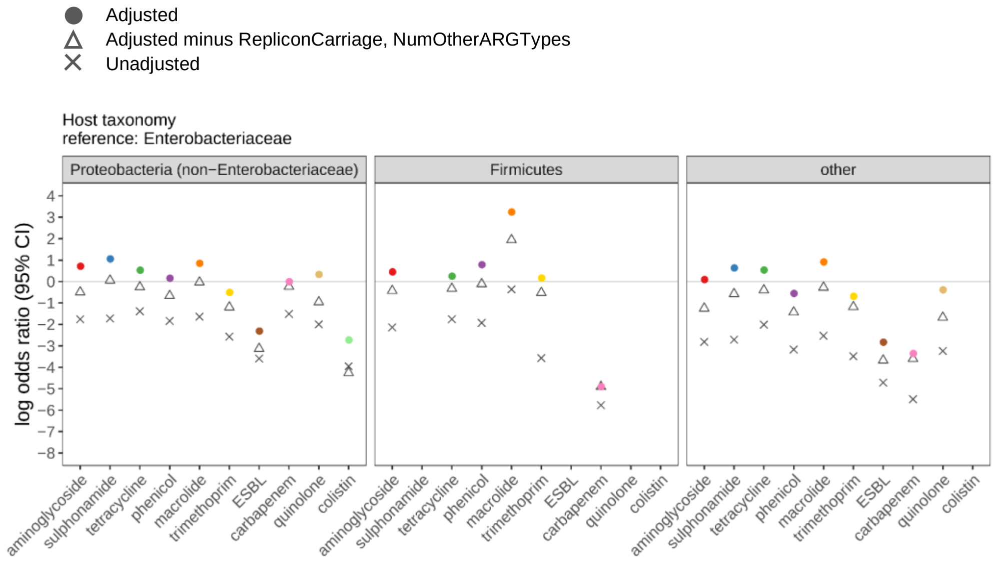

Supplementary Figure 22. The association between host taxonomy (with Enterobacteriaceae as the reference factor level) and the log-odds of antibiotic resistance carriage (y-axis), compared across 3 different analyses: log-odds ratios from the main multivariate-adjusted model (the full model, as presented in the main text) (coloured circles); log-odds ratios from a multivariable-adjusted model which omitted the explanatory variables replicon carriage, and number of other ARG types (triangles); log-odds ratios from the unadjusted analysis (crosses).

Enterobacteriaceae plasmids were predominantly from clinically-relevant source species (Supplementary Table 3); overall, compared with plasmids from other host taxonomy categories, they were more likely to encode one or more replicon types, and for a given ARG type outcome, they were more likely to encode other resistance gene types (Supplementary Figure 23). Therefore, adjusting for replicon carriage and number of other ARG types at least partially accounts for the attenuation/reversal of negative unadjusted log-odds ratios observed in non-Enterobacteriaceae taxa.

Regarding Firmicutes plasmids, in contrast to Enterobacteriaceae plasmids, they were from a mixture of clinically relevant species (e.g. *Staphylococcus aureus*, *Enterococcus faecium*) and less clinically relevant species (e.g. *Bacillus thuringiensis*) (Supplementary Table 3). Known replicon carriage appeared to be a proxy for clinical relevance with 94% and 86% of *Staphylococcus aureus* and *Enterococcus faecium*, respectively carrying detected replicons vs 10% *Bacillus thuringiensis* plasmids (Supplementary Table 3) (presumably because the PlasmidFinder replicon typing scheme has so far been developed using plasmids from clinically relevant taxa [54]). When unadjusted analysis was conducted at the species-level, *Staphylococcus aureus* and *Enterococcus faecium* were found to be more likely to carry macrolide resistance than Enterobacteriaceae plasmids, whereas plasmids from other major Firmicutes species were less likely to encode macrolide resistance (Supplementary Figure 24). Therefore, when analysing Firmicutes plasmids overall, adjustment for factors such as replicon carriage led to the reversal of the unadjusted odds ratio.

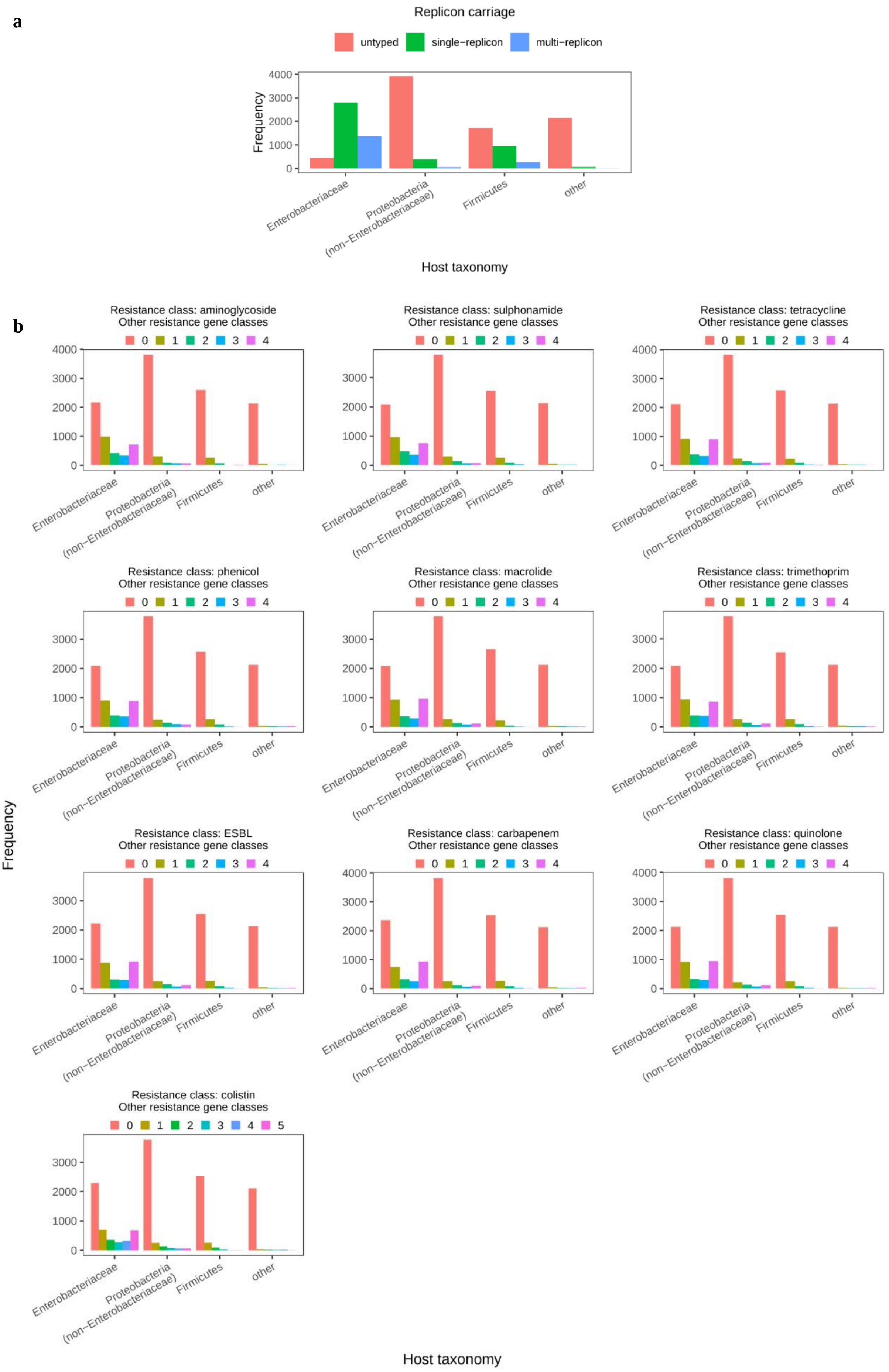
a
Replicon carriage
untyped
single−replicon
multi−replicon
Frequency
Enterobacteriaceae
Proteobacteria (non−Enterobacteriaceae)
Firmicutes
other
Host taxonomy
b
Resistance class: aminoglycoside
Other resistance gene classes
Resistance class: sulphonamide
Other resistance gene classes
Resistance class: tetracycline
Other resistance gene classes
Resistance class: phenicol
Other resistance gene classes
Resistance class: macrolide
Other resistance gene classes
Resistance class: trimethoprim
Other resistance gene classes
Resistance class: ESBL
Other resistance gene classes
Resistance class: carbapenem
Other resistance gene classes
Resistance class: quinolone
Other resistance gene classes
Resistance class: colistin
Other resistance gene classes
Frequency
Host taxonomy

Supplementary Figure 23. The association between host taxonomy and **a** replicon carriage (untyped, single-replicon, multi-replicon); **b** for a given ARG type outcome, the number of other ARG types encoded on the same plasmid (across the 10 ARG type outcomes). Enterobacteriaceae plasmids tend to encode one or more replicon loci (single/multi replicon carriage) whereas other taxa are most frequently untyped. Enterobacteriaceae plasmids encoding a resistance gene from a given type more frequently encode one or more resistance genes from other types, in comparison with plasmids from other taxa.

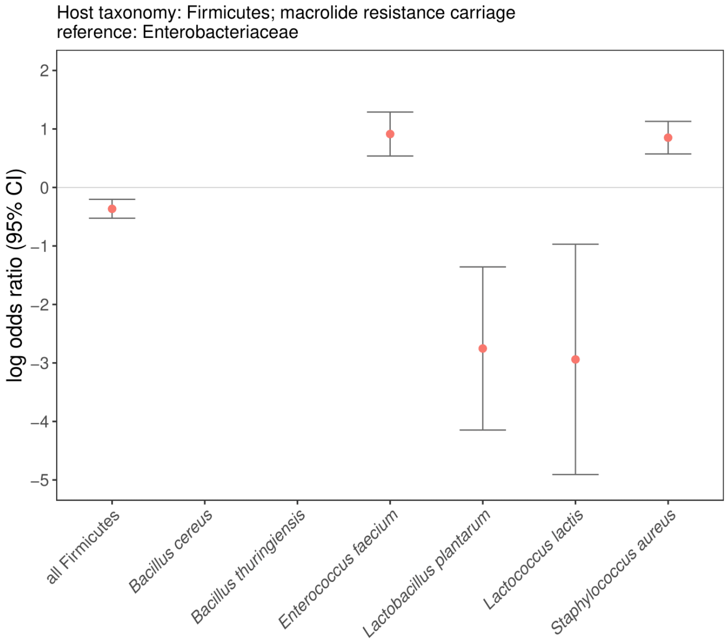

Supplementary Figure 24. Unadjusted analysis of the association between host taxonomy, broken down by Firmicutes species, and the log-odds of antibiotic resistance carriage (y-axis), across 10 ARG types. Enterobacteriaceae was the reference factor level. Firmicutes factor levels were as follows: all Firmicutes, and the most frequent Firmicutes species: *Bacillus cereus*, *Bacillus thuringiensis*, *Enterococcus faecium*, *Lactobacillus plantarum*, *Lactococcus lactis*, *Staphylococcus aureus*. Log-odds ratios indicate the effect of a given factor level, relative to reference (Enterobacteriaceae), and error bars show 95% confidence intervals. Log-odds ratios for *B. cereus* plasmids (n=104) and *B. thuringiensis* plasmids (n=306) are not shown since no plasmids from these species encoded known macrolide resistance genes.

2.7.2 Biocide/metal resistance gene presence, integron presence, number of other ARG types

Biocide/metal resistance gene presence, integron presence, and number of other ARG types, were generally positively associated with antibiotic resistance carriage. In the unadjusted analysis, strong positive associations were found across all ARG type outcomes; adjusted log-odds remained positive across most ARG type outcomes, but there was attenuation (Supplementary Figures 9, 13, 20). The three explanatory variables were positively co-associated (Supplementary Figure 25). Removing two of the three co-associated explanatory variables from the model reduced attenuation of the effects of the remaining variable in each case (Supplementary Figures 26–28), consistent with confounding bias.

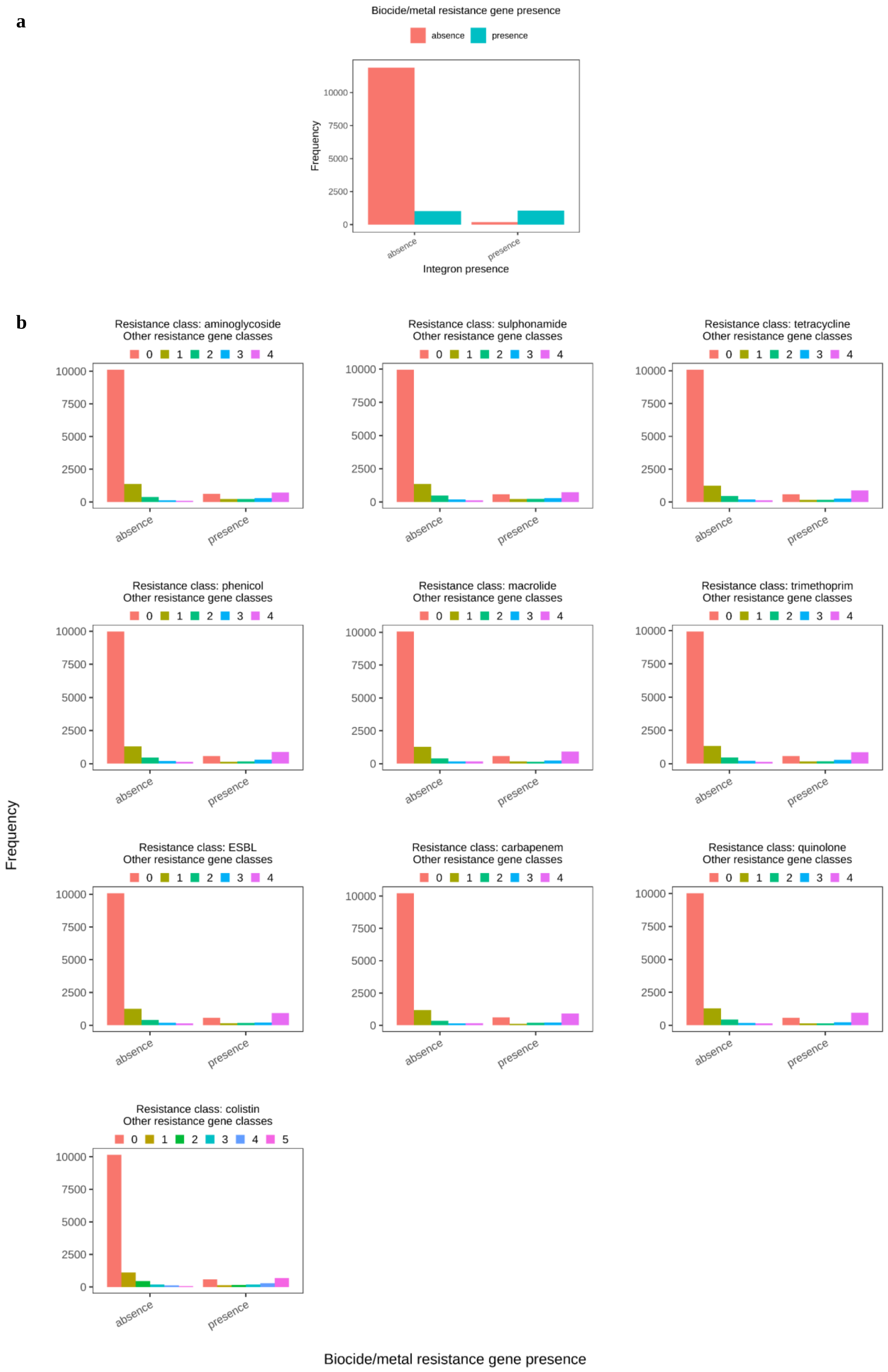
a
Biocide/metal resistance gene presence
absence
presence
Frequency
10000
7500
5000
2500
0
absence
presence
Integron presence
b
Resistance class: aminoglycoside
Other resistance gene classes
Resistance class: sulphonamide
Other resistance gene classes
Resistance class: tetracycline
Other resistance gene classes
Resistance class: phenicol
Other resistance gene classes
Resistance class: macrolide
Other resistance gene classes
Resistance class: trimethoprim
Other resistance gene classes
Resistance class: ESBL
Other resistance gene classes
Resistance class: carbapenem
Other resistance gene classes
Resistance class: quinolone
Other resistance gene classes
Resistance class: colistin
Other resistance gene classes
0 1 2 3 4 5
Frequency
Biocide/metal resistance gene presence

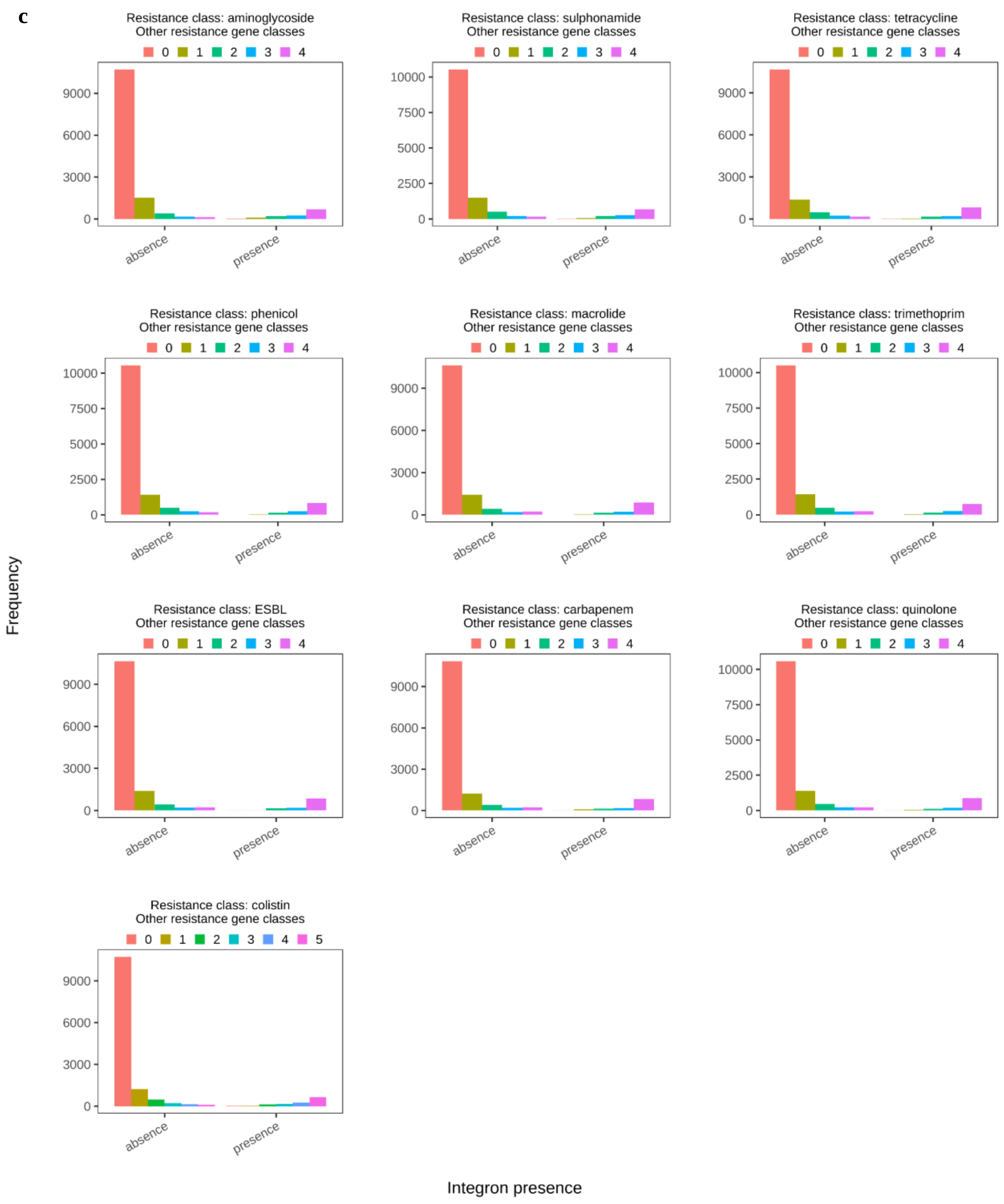

Supplementary Figure 25. The association between **a** presence of integrons and presence of biocide/metal resistance genes; **b** for a given ARG type outcome, the presence of biocide/metal resistance genes and the number of other resistance gene types encoded on the same plasmid (across 10 ARG type outcomes); **c** for a given ARG type outcome, the presence of integrons and the number of other resistance gene types encoded on the same plasmid (across 10 ARG type outcomes). Positive co-associations are found between all three explanatory variables (integron presence, biocide/metal resistance gene presence, the number of other resistance gene types).

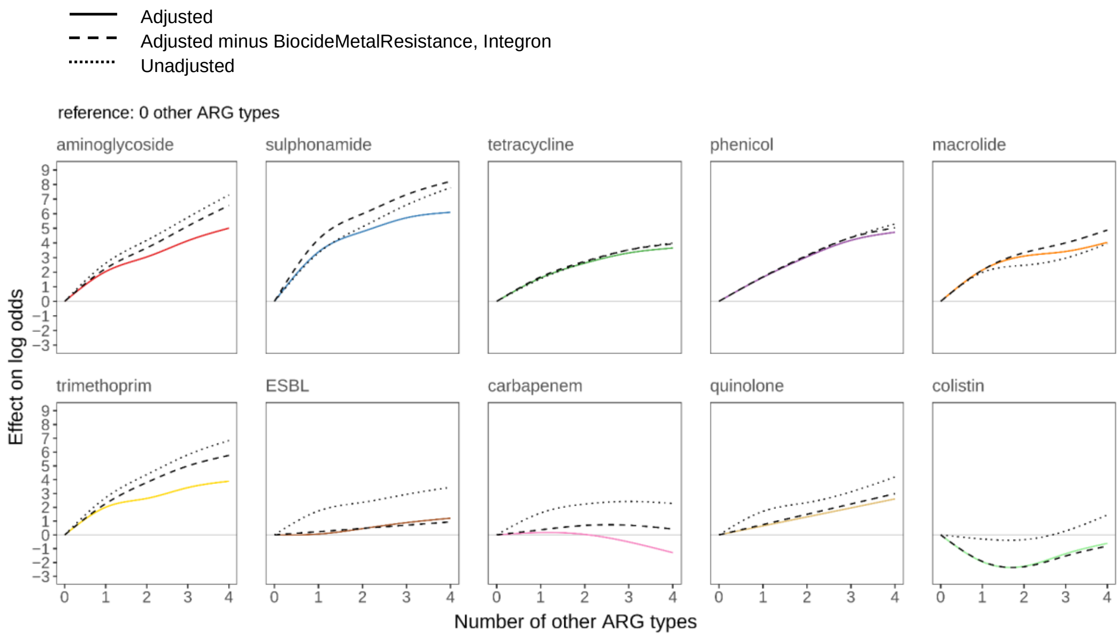

Supplementary Figure 26. The association between the number of other ARG types and the log-odds of ARG carriage (y-axis), across 10 ARG types. Smooths displayed with solid, coloured lines are from the main adjusted model (the full model, as presented in the main text). Smooths displayed with dashed, black lines are from an adjusted model which omitted the explanatory variables integron presence, and biocide/metal resistance gene presence. Smooths displayed with dotted, black lines are from an unadjusted model.

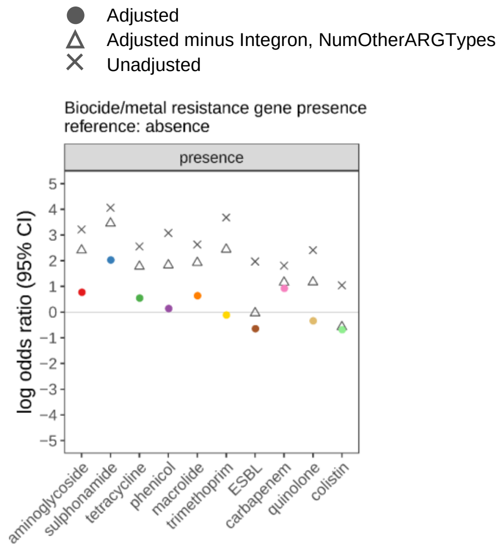

Supplementary Figure 27. The association between biocide/metal resistance gene presence (vs absence) and the log-odds of antibiotic resistance carriage (y-axis), compared across 3 different analyses: log-odds ratios from the main adjusted model (the full model, as presented in the main text) (coloured circles); log-odds ratios from an adjusted model which omitted the explanatory variables integron presence, and number of other resistance gene types (triangles); log-odds ratios from the unadjusted analysis (crosses).

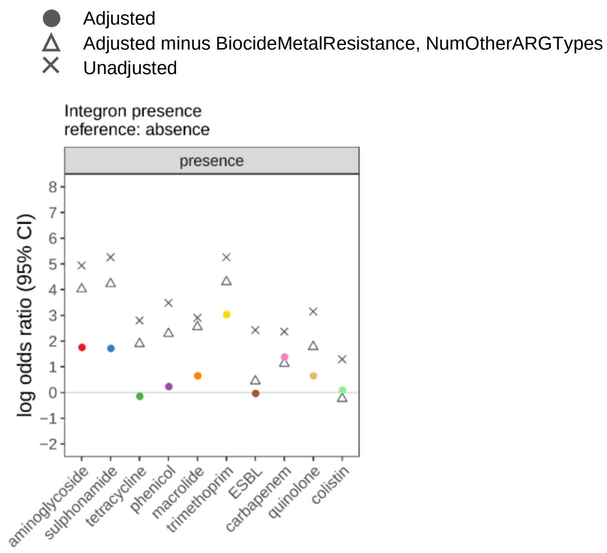

Supplementary Figure 28. The association between integron presence (vs absence) and the log-odds of antibiotic resistance carriage (y-axis), compared across 3 different analyses: log-odds ratios from the main adjusted model (the full model, as presented in the main text) (coloured circles); log-odds ratios from an adjusted model which omitted the explanatory variables biocide/metal resistance gene presence, and number of other resistance gene types (triangles); log-odds ratios from the unadjusted analysis (crosses).

2.7.3 Conjugative system

Unadjusted log-odds indicated a link between plasmid transmissibility (especially, conjugative plasmids) and resistance carriage across all ARG type outcome, relative to non-mobilisable plasmids; positive associations were attenuated in the adjusted analysis, although a positive association between conjugative plasmids and carbapenem resistance carriage remained (Supplementary Figure 10).

The association statistics indicated that conjugative system was most strongly associated with $\log_{10}$ plasmid size (moderately strong association; Spearman's $\rho = 0.74$). Mean plasmid sizes for conjugative, mobilisable, and non-mobilisable plasmids were 111 kb, 69 kb, and 67 kb, respectively, and visualising the distribution of plasmid sizes by conjugative system showed conjugative plasmids are at least ~15kb and their size distribution peaks around 100kb, whereas mobilisable and non-mobilisable plasmids do not show a bias towards being larger (Supplementary Figure 29).

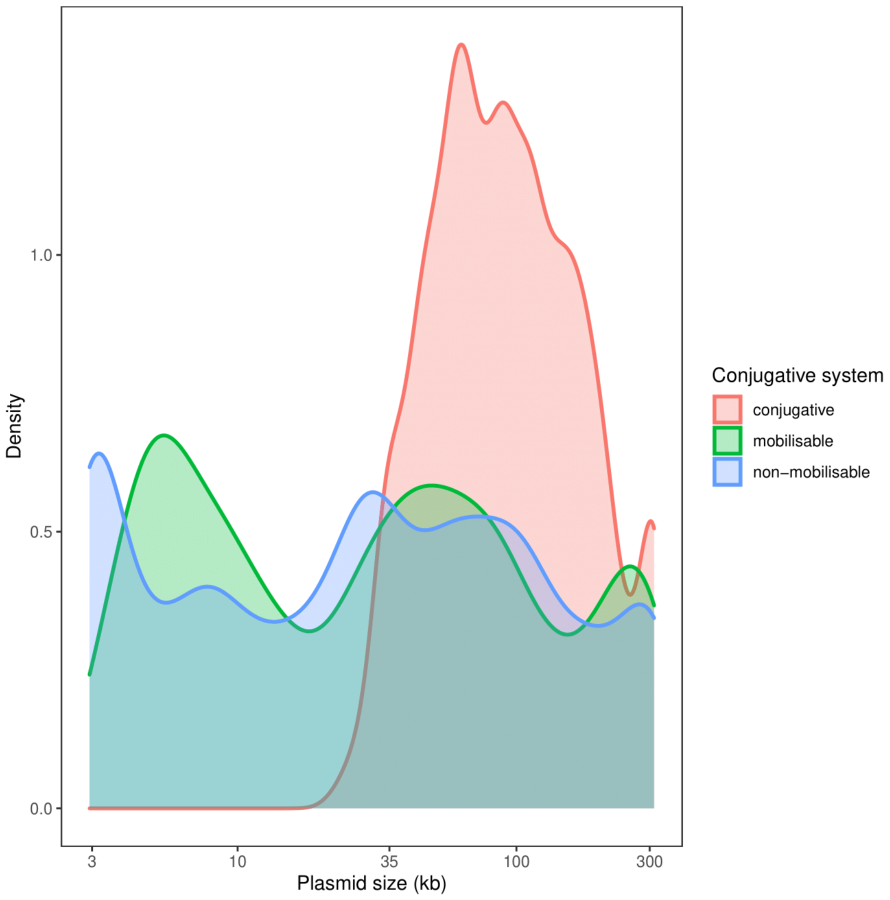

Supplementary Figure 29. Density curves showing the relationship between $\log_{10}$ plasmid size (centred on 10kb) and plasmid conjugative system (conjugative, mobilisable, non-mobilisable).

However, removing $\log_{10}$ plasmid size from the model did not substantially reduce attenuation effects (Supplementary Figure 30). Further exploration (models with additional terms removed) indicated complex confounding interrelationships; only when $\log_{10}$ plasmid size, insertion sequence density, number of other resistance gene types, integron presence, replicon carriage, and host taxonomy were removed (i.e. retaining only conjugative system, collection date, biocide/metal resistance, geographic location, isolation source, and virulence gene presence), did the log-odds ratios resemble unadjusted log-odds ratios more closely (Supplementary Figures 31, 32).

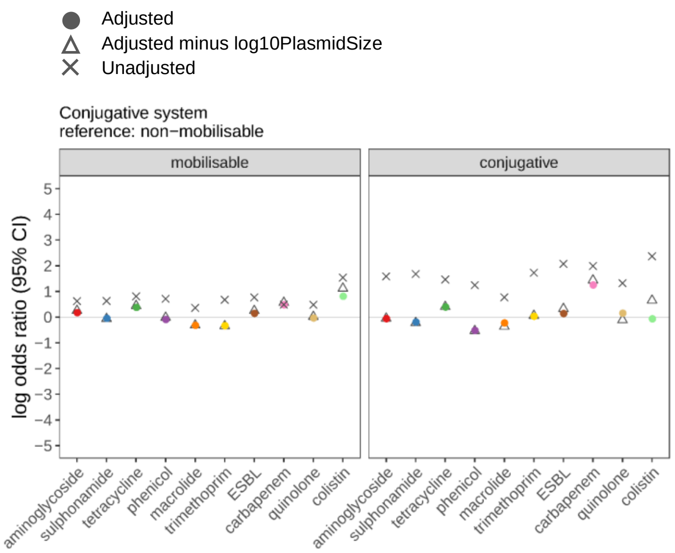

Supplementary Figure 30. The association between conjugative system (non-mobilisable [reference], mobilisable, conjugative) and the log-odds of antibiotic resistance carriage (y-axis), compared across 3 different analyses: log-odds ratios from the main adjusted model (the full model, as presented in the main text) (coloured circles); log-odds ratios from an adjusted model which omitted the explanatory variable $\log_{10}$ plasmid size (triangles); log-odds ratios from the unadjusted analysis (crosses).

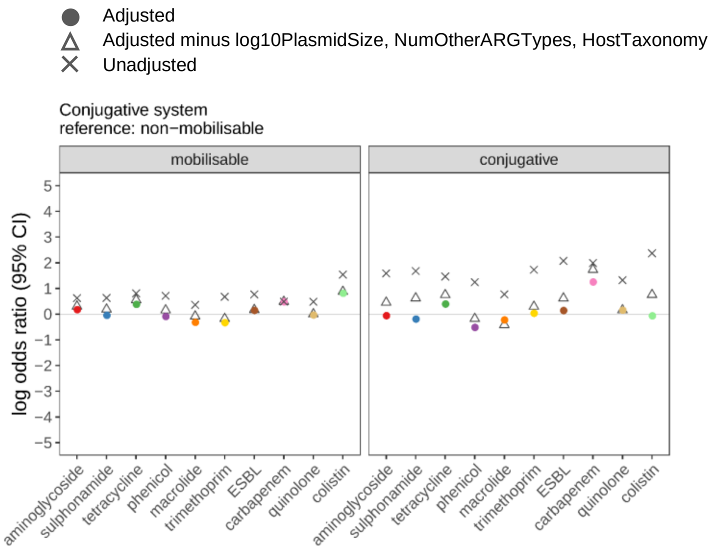

Supplementary Figure 31. The association between conjugative system (non-mobilisable [reference], mobilisable, conjugative) and the log-odds of antibiotic resistance carriage (y-axis), compared across 3 different analyses: log-odds ratios from the main adjusted model (the full model, as presented in the main text) (coloured circles); log-odds ratios from an adjusted model which omitted the explanatory variables $\log_{10}$ plasmid size, number of other ARG types, host taxonomy (triangles); log-odds ratios from the unadjusted analysis (crosses).

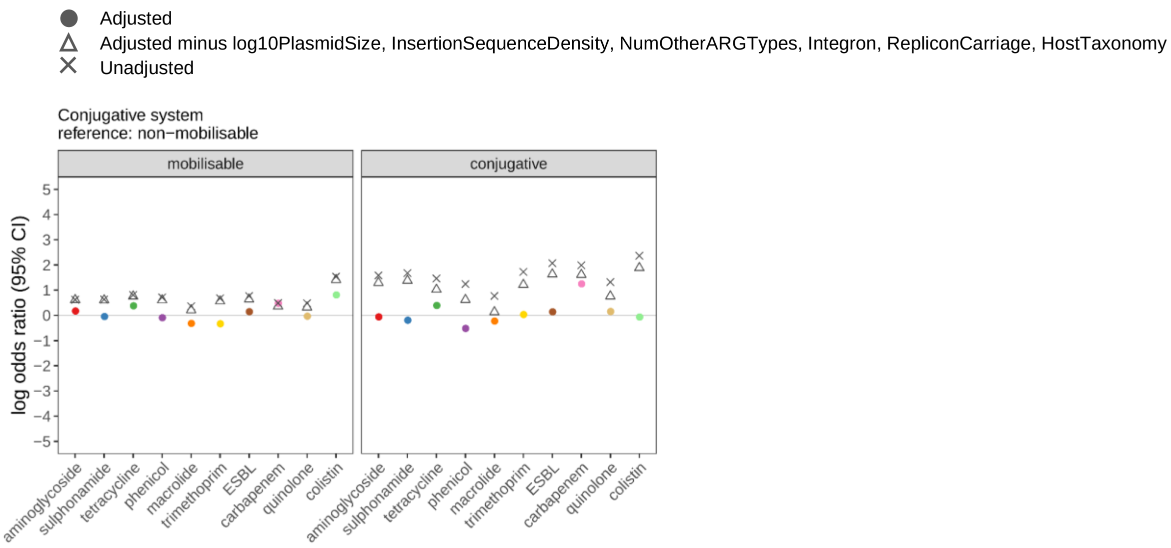

Supplementary Figure 32. The association between conjugative system (non-mobilisable [reference], mobilisable, conjugative) and the log-odds of antibiotic resistance carriage (y-axis), compared across 3 different analyses: log-odds ratios from the main adjusted model (the full model, as presented in the main text) (coloured circles); log-odds ratios from an adjusted model which omitted the explanatory variables $\log_{10}$ plasmid size, insertion sequence density, number of other resistance gene types, integron presence, replicon carriage, host taxonomy (triangles); log-odds ratios from the unadjusted analysis (crosses).

3 References

1. Orlek, A. bacterialBercow v0.1.0. Zenodo. at https://doi.org/http://doi.org/10.5281/zenodo.5076032 (2021).
2. Orlek, A. getNCBImetadata v0.1.0. Zenodo. at https://doi.org/https://doi.org/10.5281/zenodo.5076032 (2021).
3. NCBI. BioSample Attributes. https://www.ncbi.nlm.nih.gov/biosample/docs/attributes/ (2020).
4. Huerta-Cepas, J., Serra, F. & Bork, P. ETE 3: Reconstruction, Analysis, and Visualization of Phylogenomic Data. *Mol. Biol. Evol.* (2016) doi:10.1093/molbev/msw046.
5. Cooley, D. googleway: Accesses Google Maps APIs to Retrieve Data and Plot Maps. at https://github.com/SymbolixAU/googleway (2018).
6. Google Maps Platform. Places Autocomplete Service: StructuredFormatting interface. https://developers.google.com/maps/documentation/javascript/reference/places-autocomplete-service#StructuredFormatting (2020).
7. The World Bank. World Bank Country and Lending Groups. *World Bank Country and Lending Groups* https://datahelpdesk.worldbank.org/knowledgebase/articles/906519-world-bank-country-and-lending-groups (2018).
8. Alvarez-Kalverkamp, M. *et al. Meat atlas, Facts and figures about the animals we eat*. *Heinrich Böll Stiftung and Friends of the Earth Europe* (2014).
9. Gilbert, M. *et al.* Global distribution data for cattle, buffaloes, horses, sheep, goats, pigs, chickens and ducks in 2010. *Sci. Data* (2018) doi:10.1038/sdata.2018.227.
10. FAO. *The State of World Fisheries and Aquaculture 2018 - Meeting the sustainable development goals*. *FAO* (2018).
11. Mansfield, J. *et al.* Top 10 plant pathogenic bacteria in molecular plant pathology. *Molecular Plant Pathology* at https://doi.org/10.1111/j.1364-3703.2012.00804.x (2012).
12. Dietrich, M. R., Ankeny, R. A. & Chen, P. M. Publication trends in model organism research. *Genetics* (2014) doi:10.1534/genetics.114.169714.
13. Kostic, A. D., Howitt, M. R. & Garrett, W. S. Exploring host-microbiota interactions in animal models and humans. *Genes Dev.* (2013) doi:10.1101/gad.212522.112.
14. Pitzschke, A. From Bench to Barn: Plant Model Research and its Applications in Agriculture. *Adv. Genet. Eng.* (2013) doi:10.4172/2169-0111.1000110.
15. Tsai, C. J. Y., Loh, J. M. S. & Proft, T. Galleria mellonella infection models for the study of bacterial diseases and for antimicrobial drug testing. *Virulence* at https://doi.org/10.1080/21505594.2015.1135289 (2016).
16. IJSEM. IJSEM Culture Collection Abbreviations: Abbreviations of culture collections cited in the Validation Lists. https://www.microbiologyresearch.org/marketing/editorial/IJSEM_Culture_Collection_Abbreviation_14082015.pdf (2015).
17. Kazil, J. & Jarmul, K. Chapter 7: Data Cleanup: Investigation, Matching, and Formatting. in *Data Wrangling with Python: Tips and Tools to Make Your Life Easier* 178–181 (O'Reilly Media, 2016).
18. Reimer, L. C., Söhngen, C., Vetcininova, A. & Overmann, J. Mobilization and integration of bacterial phenotypic data—Enabling next generation biodiversity analysis through the BacDive metadatabase. *Journal of Biotechnology* at https://doi.org/10.1016/j.jbiotec.2017.05.004 (2017).
19. Reimer, L. C. *et al.* BacDive in 2019: Bacterial phenotypic data for High-throughput

biodiversity analysis. *Nucleic Acids Res.* (2019) doi:10.1093/nar/gky879.
20. Ondov, B. D. *et al.* Mash: Fast genome and metagenome distance estimation using MinHash. *Genome Biol.* (2016) doi:10.1186/s13059-016-0997-x.
21. Kolaczyk, E. D. & Csardi, G. *Statistical Analysis of Network Data with R*. (Springer, 2014).
22. Fortunato, S. & Hric, D. Community detection in networks: A user guide. *Physics Reports* at https://doi.org/10.1016/j.physrep.2016.09.002 (2016).
23. Carattoli, A. & Hasman, H. PlasmidFinder and In Silico pMLST: Identification and Typing of Plasmid Replicons in Whole-Genome Sequencing (WGS). in *Methods in Molecular Biology* (2020). doi:10.1007/978-1-4939-9877-7_20.
24. Zankari, E. *et al.* Identification of acquired antimicrobial resistance genes. *J. Antimicrob. Chemother.* **67**, 2640–2644 (2012).
25. Bortolaia, V. *et al.* ResFinder 4.0 for predictions of phenotypes from genotypes. *J. Antimicrob. Chemother.* **75**, (2020).
26. Pal, C., Bengtsson-Palme, J., Rensing, C., Kristiansson, E. & Larsson, D. G. J. BacMet: Antibacterial biocide and metal resistance genes database. *Nucleic Acids Research* at https://doi.org/10.1093/nar/gkt1252 (2014).
27. Chen, L., Zheng, D., Liu, B., Yang, J. & Jin, Q. VFDB 2016: Hierarchical and refined dataset for big data analysis - 10 years on. *Nucleic Acids Res.* (2016) doi:10.1093/nar/gkv1239.
28. Cury, J., Abby, S. S., Doppelt-Azeroual, O., Neron, B. & Rocha, E. P. C. Chapter 19: Identifying Conjugative Plasmids and Integrative Conjugative Elements with CONJscan. in *Horizontal Gene Transfer: Methods and Protocols* (ed. de la Cruz, F.) 265–283 (Humana Press, 2020).
29. Hyatt, D. *et al.* Prodigal: Prokaryotic gene recognition and translation initiation site identification. *BMC Bioinformatics* (2010) doi:10.1186/1471-2105-11-119.
30. Garcillan-Barcia, M. P., Redondo-Salvo, S., Vielva, L. & de la Cruz, F. Chapter 21: MOBscan: Automated Annotation of MOB Relaxases. in *Horizontal Gene Transfer: Methods and Protocols* (ed. de la Cruz, F.) 295–308 (2020).
31. Cury, J., Touchon, M. & Rocha, E. P. C. Integrative and conjugative elements and their hosts: Composition, distribution and organization. *Nucleic Acids Res.* (2017) doi:10.1093/nar/gkx607.
32. Cury, J., Jové, T., Touchon, M., Néron, B. & Rocha, E. P. Identification and analysis of integrons and cassette arrays in bacterial genomes. *Nucleic Acids Res.* (2016) doi:10.1093/nar/gkw319.
33. Xie, Z. & Tang, H. ISEScan: automated identification of insertion sequence elements in prokaryotic genomes. *Bioinformatics* (2017) doi:10.1093/bioinformatics/btx433.
34. Wood, S. N. *Generalized additive models: An introduction with R, second edition*. *Generalized Additive Models: An Introduction with R, Second Edition* (2017). doi:10.1201/9781315370279.
35. Wood, S. N. Fast stable restricted maximum likelihood and marginal likelihood estimation of semiparametric generalized linear models. *J. R. Stat. Soc. Ser. B Stat. Methodol.* (2011) doi:10.1111/j.1467-9868.2010.00749.x.
36. Wood, S. N. Chapter 4: Introducing GAMs. in *Generalized Additive Models: An Introduction with R, Second Edition* 185 (2017).
37. Fasiolo, M., Nedellec, R., Goude, Y. & Wood, S. N. Scalable Visualization Methods for Modern Generalized Additive Models. *J. Comput. Graph. Stat.* (2020) doi:10.1080/10618600.2019.1629942.
38. Barrett, T. *et al.* BioProject and BioSample databases at NCBI: Facilitating capture and organization of metadata. *Nucleic Acids Res.* **40**, (2012).

39. Douarre, P. E., Mallet, L., Radomski, N., Felten, A. & Mistou, M. Y. Analysis of COMPASS, a New Comprehensive Plasmid Database Revealed Prevalence of Multireplicon and Extensive Diversity of IncF Plasmids. *Front. Microbiol.* (2020) doi:10.3389/fmicb.2020.00483.
40. Campos, F. S. *et al.* Complete Sequences of Two Plasmids Found in a Brazilian Bacillus thuringiensis Serovar israelensis Strain . *Microbiol. Resour. Announc.* (2019) doi:10.1128/mra.00051-19.
41. Bolotin, A. *et al.* Comparative genomics of extrachromosomal elements in Bacillus thuringiensis subsp. israelensis. *Res. Microbiol.* (2017) doi:10.1016/j.resmic.2016.10.008.
42. Labbé, G. *et al.* Complete genome sequences of 17 Canadian isolates of Salmonella enterica subsp. enterica serovar Heidelberg from human, animal, and food sources. *Genome Announc.* (2016) doi:10.1128/genomeA.00990-16.
43. LaBreck, P. T. *et al.* Conjugative transfer of a novel staphylococcal plasmid encoding the biocide resistance gene, QacA. *Front. Microbiol.* (2018) doi:10.3389/fmicb.2018.02664.
44. Ohtsubo, Y. *et al.* Complete genome sequence of Sphingopyxis macrogoltabida strain 203N (NBRC 111659), a polyethylene glycol degrader. *Genome Announc.* (2016) doi:10.1128/genomeA.00529-16.
45. Ohtsubo, Y. *et al.* Complete genome sequence of Sphingopyxis macrogoltabida type strain NBRC 15033, originally isolated as a polyethylene glycol degrader. *Genome Announc.* (2015) doi:10.1128/genomeA.01401-15.
46. Bukowski, M. *et al.* Prevalence of Antibiotic and Heavy Metal Resistance Determinants and Virulence-Related Genetic Elements in Plasmids of Staphylococcus aureus. *Front. Microbiol.* (2019) doi:10.3389/fmicb.2019.00805.
47. Ou, H. Y. *et al.* Complete genome sequence of hypervirulent and outbreak-associated Acinetobacter baumannii strain LAC-4: Epidemiology, resistance genetic determinants and potential virulence factors. *Sci. Rep.* (2015) doi:10.1038/srep08643.
48. Satou, K. *et al.* Complete genome sequences of low-passage virulent and high-passage avirulent variants of pathogenic Leptospira interrogans serovar Manilae strain UP-MMCNIID, originally isolated from a patient with severe leptospirosis, determined using PacBio single-mole. *Genome Announc.* (2015) doi:10.1128/genomeA.00882-15.
49. Freese, H. M. *et al.* Trajectories and Drivers of Genome Evolution in Surface-Associated Marine Phaeobacter. *Genome Biol. Evol.* (2017) doi:10.1093/gbe/evx249.
50. Kröger, C. *et al.* The transcriptional landscape and small RNAs of Salmonella enterica serovar Typhimurium. *Proc. Natl. Acad. Sci. U. S. A.* (2012) doi:10.1073/pnas.1201061109.
51. Azuma, Y. *et al.* Whole-genome analyses reveal genetic instability of Acetobacter pasteurianus. *Nucleic Acids Res.* (2009) doi:10.1093/nar/gkp612.
52. Chen, F. J., Lauderdale, T. L., Wang, L. S. & Huang, I. W. Complete genome sequence of Staphylococcus aureus Z172, a vancomycin-intermediate and daptomycinnonsusceptible methicillin-resistant strain isolated in Taiwan. *Genome Announc.* (2013) doi:10.1128/genomeA.01011-13.
53. Holden, M. T. G. *et al.* Genome sequence of a recently emerged, highly transmissible, multi-antibiotic- and antiseptic-resistant variant of methicillin-resistant Staphylococcus aureus, sequence type 239 (TW). *J. Bacteriol.* (2010) doi:10.1128/JB.01255-09.
54. Carattoli, A. *et al.* In Silico detection and typing of plasmids using plasmidfinder and plasmid multilocus sequence typing. *Antimicrob. Agents Chemother.* **58**, 3895–3903 (2014).
